# Structure‐Guided Engineering of a Promiscuous *O*‐Methyltransferase for a SAM Regeneration Biocatalysis Platform of Methylated Pharmaceuticals

**DOI:** 10.1002/advs.202517794

**Published:** 2025-12-19

**Authors:** Xiran Xiong, Jun Song, Shihan Li, Lu Jin, Qi He, Baohui Zhang, Yan Cao, Shanyong Yi, Yanfang Yang, Xiang Li, Juan Li, Wei Huang

**Affiliations:** ^1^ School of Pharmacy Hubei University of Chinese Medicine Wuhan China; ^2^ Hubei Shizhen Laboratory Hubei University of Chinese Medicine Wuhan China; ^3^ Traditional Chinese Medicine Institute of Anhui Dabie Mountain West Anhui University Luan China; ^4^ Department of Neurosurgery Zhongnan Hospital of Wuhan University Wuhan China

**Keywords:** fusion enzyme, plant O‐methyltransferase, SAM regeneration, structure, substrate promiscuity

## Abstract

*O*‐Methylation catalyzed by plant *O*‐methyltransferase plays a crucial role in both drug design and biosynthesis of natural products. However, their practical applications are often restricted by strict substrate specificity and a strong dependence on the expensive methyl donor S‐adenosyl‐L‐methionine (SAM). Herein, an *O*‐methyltransferase, SmOMT, is identified from the medicinal plant *Selaginella moellendorffii*, exhibiting substrate promiscuity and regioselectivity. SmOMT catalyzed the methylation of 25 structurally diverse substrates and demonstrated detectable *N*‐methylation activity. Combined ternary complex structure and molecular dynamics studies of SmOMT elucidate its catalytic and regioselectivity mechanisms. A double mutant, SmOMT^M2^, with enhanced catalytic activity is obtained based on structural analysis. To overcome SAM dependence, a cascade system for SAM regeneration is successfully constructed by coupling SmOMT^M2^ with a mutant halide methyltransferase, AtHMT^V140T^. Employing the iMARS platform, a highly active fusion enzyme, AtHMT^V140T^‐L_95_‐SmOMT^M2^, is designed. This fusion enzyme outperforms the free‐enzyme cascade system and facilitates the gram‐scale synthesis of a series of methylated compounds with enhanced anti‐inflammatory activity. This work provides a versatile methylating biocatalyst and establishes an efficient SAM regeneration methylation platform, overcoming limitations in enzymatic methylation and enabling the sustainable production of high‐value pharmaceuticals.

## Graphical abstract

A substrate promiscuous and regioselective *O*‐methyltransferase, SmOMT, is functionally and structurally characterized. A double mutant, SmOMT^E152A/I306A^, exhibited enhanced catalytic activity. By coupling this mutant with a mutant halide methyltransferase, AtHMT^V140T^, for SAM regeneration, a superior artificial fusion enzyme, AtHMT^V140T^‐L_95_‐SmOMT^E152A/I306A^, is constructed. This platform enables efficient biosynthesis of bioactive methylated compounds.

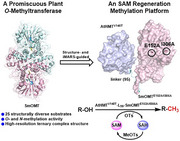

## 1 Introduction

The methyl group is one of the most prevalent substituents in drug molecules, surpassed only by hydrogen and nitrogen [1]. According to statistical information from the Njardarson group, more than half of the top 200 best‐selling small‐molecule drugs in 2023 contained at least one methyl group [2]. Despite its small size, the methyl group exerts a profound impact on the molecular properties of the drugs. Strategic methyl incorporation can enhance biological activity, modulate metabolic stability, alter solubility, improve target selectivity, and, in some cases, convert an agonist into an antagonist [3, 4, 5]. Medicinal chemists have termed this phenomenon the “magic methyl effect” [6, 7, 8]. For example, methylation of the abandoned compound mevastatin yielded the noncarcinogenic drug lovastatin. Further methylation produced simvastatin, which has double the half‐life of that of lovastatin, as well as boosted efficacy and achieved annual sales of $5.6 billion [9]. Similarly, α‐methylation of the proline residue in Glypromate yielded trofinetide, which exhibits a prolonged plasma half‐life and improved oral bioavailability, overcoming the rapid metabolism and poor delivery of the prototype, thereby enabling its approval by the United States Food and Drug Administration (US FDA) for the treatment of Rett syndrome [10].

Furthermore, methylation is ubiquitous in the biosynthesis of natural products (NPs), contributing to their structural diversity and bioactivity [11, 12, 13]. In plants, methylation is catalyzed by S‐adenosyl‐L‐methionine (SAM)‐dependent methyltransferases (MTs; EC 2.1.1.) [14]. MTs are classified into C–, O–, N–, and S–MTs based on the atom targeted for methylation [15]. Among these, *O*‐methyltransferases (OMTs) constitute the largest and most functionally diverse group and are consequently the most extensively studied [16, 17]. Relative to chemical synthesis, enzymatic *O*‐methylation offers distinct advantages. It enables precise regio‐ and stereoselective modifications—even within complex substrate mixtures—thereby circumventing the need for costly multistep protection–deprotection strategies and eliminating reliance on toxic methylating reagents [18]. Moreover, enzymatic *O*‐methylation is a green catalytic approach that proceeds under mild conditions and delivers high yields, making it an ideal platform for synthesizing methylated bioactive molecules [19].

In recent years, an increasing number of functionally characterized plant OMTs have been identified and used for the enzymatic synthesis of NPs [20, 21, 22]. Although significant progress has been made in identifying and characterizing plant OMTs, research has primarily focused on the methylation of flavonoids, alkaloids, and coumarins [23]. Most reported plant OMTs exhibit strict substrate selectivity and narrow substrate scopes, limiting their utility in the biocatalytic synthesis of other structurally diverse NPs [24]. OMTs with substrate promiscuity are generally considered promising catalysts for small‐molecule methylation [25]. However, only a few OMTs with moderate substrate promiscuity have been identified. For instance, AdOMT1 from *Angelica decursiva* methylates nine substrates, including coumarins, caffeic acid, and flavonoids [26]. Similarly, AsOMT1−4 from *Aquilaria sinensis* demonstrates catalytic activity toward 2‐(2‐phenylethyl) chromones, flavonoids, and coumarins [27], while COMT‐S from *Peucedanum praeruptorum* exhibits broad substrate preference, catalyzing the methylation of structurally diverse coumarins [28].

Structural studies have revealed that plant OMTs catalyze MT‐mediated methylation via an S_N_2‐like nucleophilic substitution mechanism [29, 30, 31]. However, the structural basis underlying the observed substrate promiscuity and regioselectivity remains poorly understood [32]. The large‐scale application of OMTs is also hindered by two major limitations: the high cost and limited availability of SAM, and potent product inhibition caused by S‐adenosyl‐L‐homocysteine (SAH), which accumulates after methyl transfer [33]. Although both in vivo and in vitro SAM regeneration systems have been developed, the significant decrease in methylation efficiency due to interference between multiple free enzymes in the system remains a persistent challenge to be addressed [34].

**FIGURE 1:**
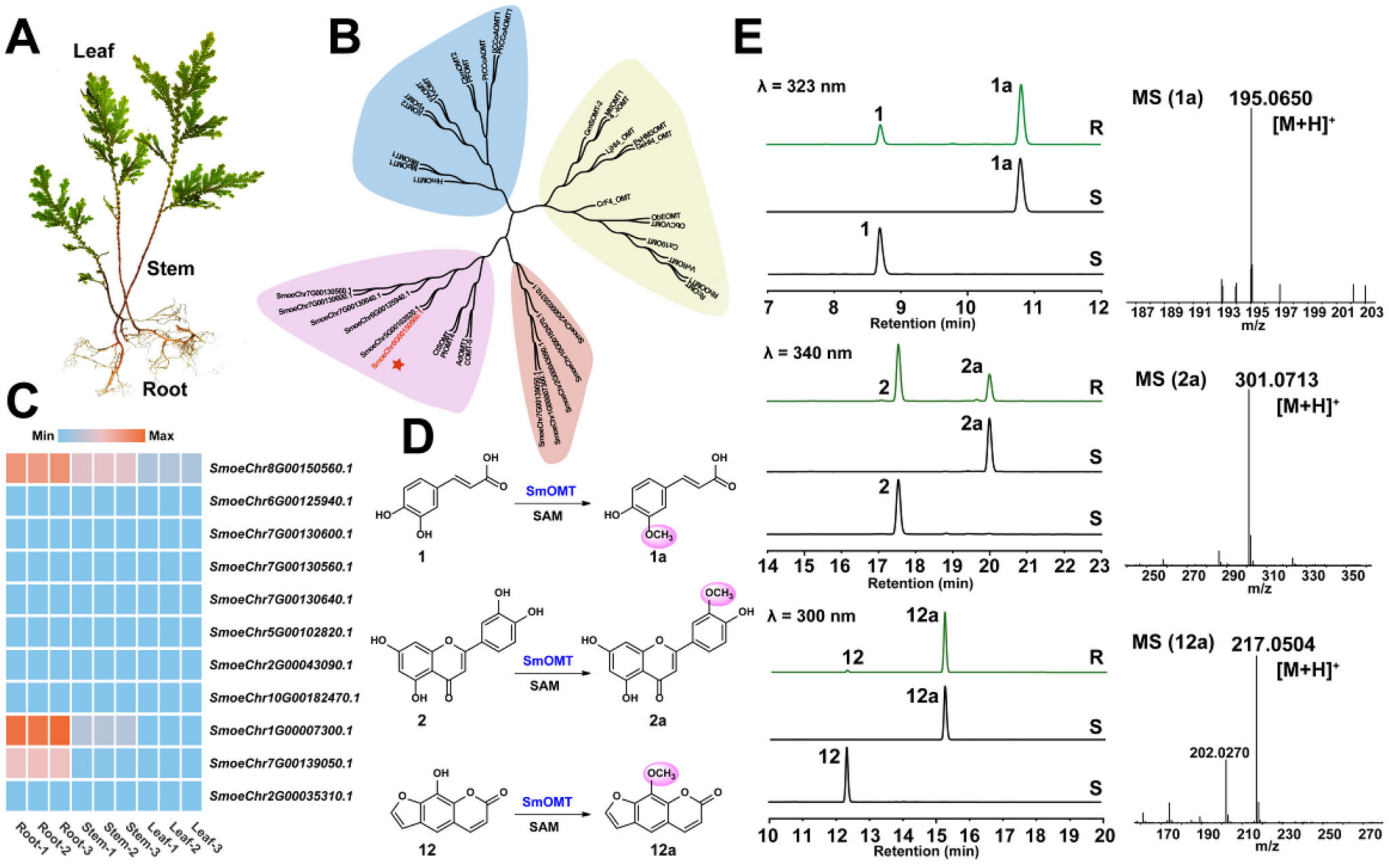
Screening and Functional Characterization of Candidate OMT Genes. (A) Plant morphology of *S. moellendorffii*. (B) Phylogenetic tree of candidate *S. moellendorffii* OMT proteins and their homologs from other plants. (C) Expression levels of candidate *S. moellendorffii* OMT genes in root, stem, and leaf tissues. Values represent z‐score‐transformed log (TPM + 1). (D) SmOMT catalyzed *O*‐methylation of compounds **1**, **2,** and **12**. Reaction conditions: Reactions were performed at 37°C for 12 h. (E) HPLC profiles of enzymatic reactions for substrates **1**, **2**, and **12**, and mass spectrometric characterization of the methylated products **1a**, **2a**, and **12a**. (R): Reaction group; (S): Reference standards. MS data were acquired in positive ion mode.

*Selaginella moellendorffii* is an ancient medicinal fern and the primary component of the traditional Chinese medicine “Jiangnan Juanbai tablets”, which are clinically used to treat blood‐heat–related conditions such as subcutaneous hemorrhage and purpura (Figure 1A) [35, 36]. This species is rich in diverse *O*‐methylated NPs, including flavonoids, phenylpropanoids, phenolic acids, and alkaloids, exhibiting methylation at various positions [37, 38, 39]. Such diversity suggests the potential presence of OMTs with both substrate promiscuity and catalytic flexibility. Herein, a plant OMT (SmOMT) was identified in *S. moellendorffii* that is capable of regioselectively methylating a broad spectrum of molecular scaffolds. Structural analysis of SmOMT complexes revealed the molecular basis of its regioselectivity. Building upon these insights, a rational fusion–protein design strategy was used to create an efficient biocatalytic system for an economical *O*‐methylation system. This system successfully overcomes the limitations of SAM dependency and mitigates the inefficiencies associated with conventional multi‐enzyme systems. Finally, employing this engineered fusion enzyme, the gram‐scale synthesis of several methylated NPs was achieved with a marked enhancement in anti‐inflammatory activity.

## 2 Results and Discussion

### 2.1 Screening and Functional Characterization of Candidate OMT Genes

To identify plant OMTs with substrate promiscuity, BLAST analysis was performed against the *S. moellendorffii* transcriptome database using two previously reported OMTs (AdOMT1 and COMT‐S) possessing substrate diversity as probes [26, 28]. The top 10 homologous genes from each search were pooled, yielding 11 candidate genes for further analysis. Phylogenetic tree analysis classified these genes into two distinct clades, with five genes clustering closely with AdOMT1 and COMT‐S (Figure 1B). Tissue‐specific expression profiling revealed that genes displayed low transcript levels for most genes in roots, stems, and leaves. Only three genes, *SmoeChr8G00150560.1*, *SmoeChr1G00007300.1*, and *SmoeChr7G00139050.1*, exhibited relatively higher expression in roots, with *SmoeChr8G00150560.1* showing the highest expression in stems and leaves (Figure 1C). Three candidate genes were cloned into a pET28a vector and heterologously expressed in *E. coli* Rosetta (DE3). Their catalytic activities were initially screened using cell lysates and subsequently confirmed with Ni‐NTA‐purified proteins (Figure S1). Assays were performed with three structurally distinct natural product substrates—caffeic acid (1), luteolin (2), and xanthotoxol (12)—using SAM as the methyl donor. High‐performance liquid chromatography (HPLC) analysis revealed that only *SmoeChr8G00150560.1* generated a new methylated product across all three substrates (Table S1). This gene was designated as *SmOMT* (GenBank: XP_002963872.1). *SmOMT* contains a 1062 bp open reading frame encoding a 354 amino acid protein and shares the highest sequence similarity (51.8%) with COMT‐S [28].

To validate the catalytic function, SmOMT was purified using size exclusion chromatography (SEC) (Figure S2). Enzymatic reactions (100 µL) were performed using a 0.5 mm substrate, 2 mm SAM, 50 mm Tris‐HCl (pH 7.5), and 100 µg SmOMT and were incubated 12 h at 37°C (Figure 1D). Liquid chromatography–mass spectrometry (LC–MS) analysis revealed that compound **1** was converted to product **1a** with an 85% conversion. Product **1a** exhibited identical retention time to the ferulic acid, with an [M+H]⁺ ion at *m/z* 195.0652, matching the exact mass calculated for ferulic acid (Figure 1E). Compound **2** yielded product **2a** with a conversion of 35%. Product **2a** displayed identical retention time to chrysoeriol, with an [M+H]⁺ peak at *m/z* 301.0707, consistent with the molecular mass of chrysoeriol (Figure 1E). Compound **12** yielded product **12a** with a 98% conversion. Product **12a** had an identical retention time to xanthotoxin, with an [M+H]⁺ ion at *m/z* 217.0504, corresponding to the molecular mass of xanthotoxin (Figure 1E). These results demonstrate that SmOMT specifically catalyzes mono‐*O*‐methylation at the 3‐OH of caffeic acid, 3′‐OH of luteolin, and 8‐OH of xanthotoxol.

Biochemical characterizations using compound **1** as the substrate showed that SmOMT exhibited maximum activity at 37°C after 4 h incubation at pH 8.0 (50 mm Tris‐HCl) or pH 7.0 (50 mm Na_2_HPO_4_/NaH_2_PO_4_). Enzymatic activity was independent of divalent metal ions (Figure S3). Kinetic analysis established a Michaelis constant (*K*_m_) of 243.5 µm for compound **1** and a catalytic efficiency (*K*_m_/*k*_cat_) of 0.068 s^−1^ mm ^−1^ (Figure S4).

### 2.2 Investigation of Substrate Promiscuity of SmOMT

To investigate the substrate promiscuity of SmOMT, a library of 35 structurally diverse compounds was screened, including flavonoids (**2–11**), coumarins (**12–17**), acetophenones (**18–32**), resveratrol (**33**), and anthraquinones (**34–35**). Compounds **17** and **21** contained ─NH_2_ groups to evaluate potential *N*‐methylation activity (Figure 2B). LC–MS analysis revealed that SmOMT exhibited substrate promiscuity, catalyzing methylation of 25 substrates (Figure 2A; Figures S5–S25).

**FIGURE 2:**
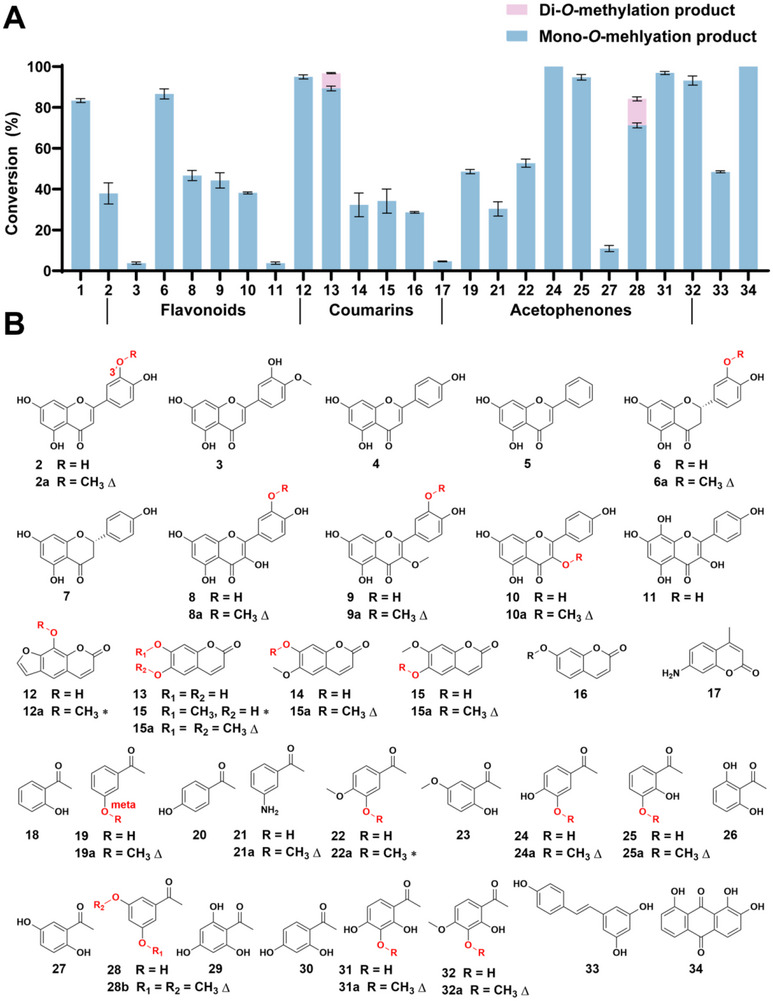
Investigation of Substrate Promiscuity of SmOMT. (A) Conversion rates of methylation reactions catalyzed by SmOMT. Blue and purple bars represent products with one and two methyl moieties added, respectively. Reactions were performed at 37°C for 12 h. (B) Chemical structures of substrates **1–34**. Δ, products purified and characterized by NMR in this study. ∗, products identified by comparison with reference standards. Data are presented as mean ± SD (*n* = 3).

SmOMT methylated seven flavonoids (**2**, **3**, **6**, and **8–11**) with conversion ranging from 5% to 80%, producing exclusively monomethylated products. This finding indicates strict regioselectivity toward flavonoids (Figure 2A). To determine the methylation sites, products **2a**, **6a**, **8a**, **9a,** and **10a** were purified and structurally characterized using nuclear magnetic resonance (NMR) analyses, including ^1^H NMR, ^13^C NMR, and heteronuclear multiple bond correlation (HMBC) (Figures S26–S40). The results identified products **2a**, **6a**, **8a**, and **9a** as 3′‐methoxyflavones, and product **10a** as 3‐methoxykaempferol. These results demonstrate the SmOMT preference for 3′‐OH methylation in flavonoids. SmOMT methylated all six tested coumarins (**12–17**) with conversion of 5%–95%. With the exception of esculetin (**13**), which generated dimethylated products, all other coumarin substrates yielded exclusively mono‐methylated derivatives (Figure 2A; Figures S41–S46). For esculetin (**13**), two methylated products were detected: the major product, which displayed identical retention time to isoscopoletin (**15**); a minor product, corresponding to scopoletin (**15a**). These results suggest that SmOMT exhibits limited regioselectivity toward coumarins. Notably, methylation of 7‐amino‐4‐methylcoumarin (**17**) was detected, confirming weak *N*‐methylation activity. SmOMT catalyzed the methylation of nine acetophenone derivatives (**19**, **21**, **22**, **24**, **25**, **27**, **28**, **31**, and **32**) with conversion ranging from 15% to 100% (Figure 2A; Figures S47–S67). Nearly all substrates yielded single monomethylated derivatives, indicating strict regioselectivity toward acetophenones. Structural elucidation of products **19a**, **21a, 24a**, **25a**, **28b**, **31a**, and **32a** through NMR and comparison with authentic standards confirmed that methylation occurred at meta‐hydroxyl positions (3′‐OH/5′‐OH), demonstrating a strong preference of SmOMT for meta‐hydroxyl groups in acetophenones. The methylation of the amino group‐containing substrate **21** afforded product **21a**, whose structure was determined by NMR analysis as 1‐[3‐(methylamino)phenyl]ethanone, further confirming the dual *O*‐/*N*‐methylation capability of SmOMT (Figures S50–S52). Additionally, SmOMT effectively methylated resveratrol (**33**) and anthraquinone (**34**; containing meta‐hydroxyl groups). These results demonstrate that SmOMT possesses substrate promiscuity, surpassing that of previously reported plant OMTs [26, 28]. This enzyme represents a powerful biocatalyst for regioselective methylation of NPs with great application potential for drug discovery.

### 2.3 Overall Structure of SmOMT and Its Complex With SAH

**FIGURE 3:**
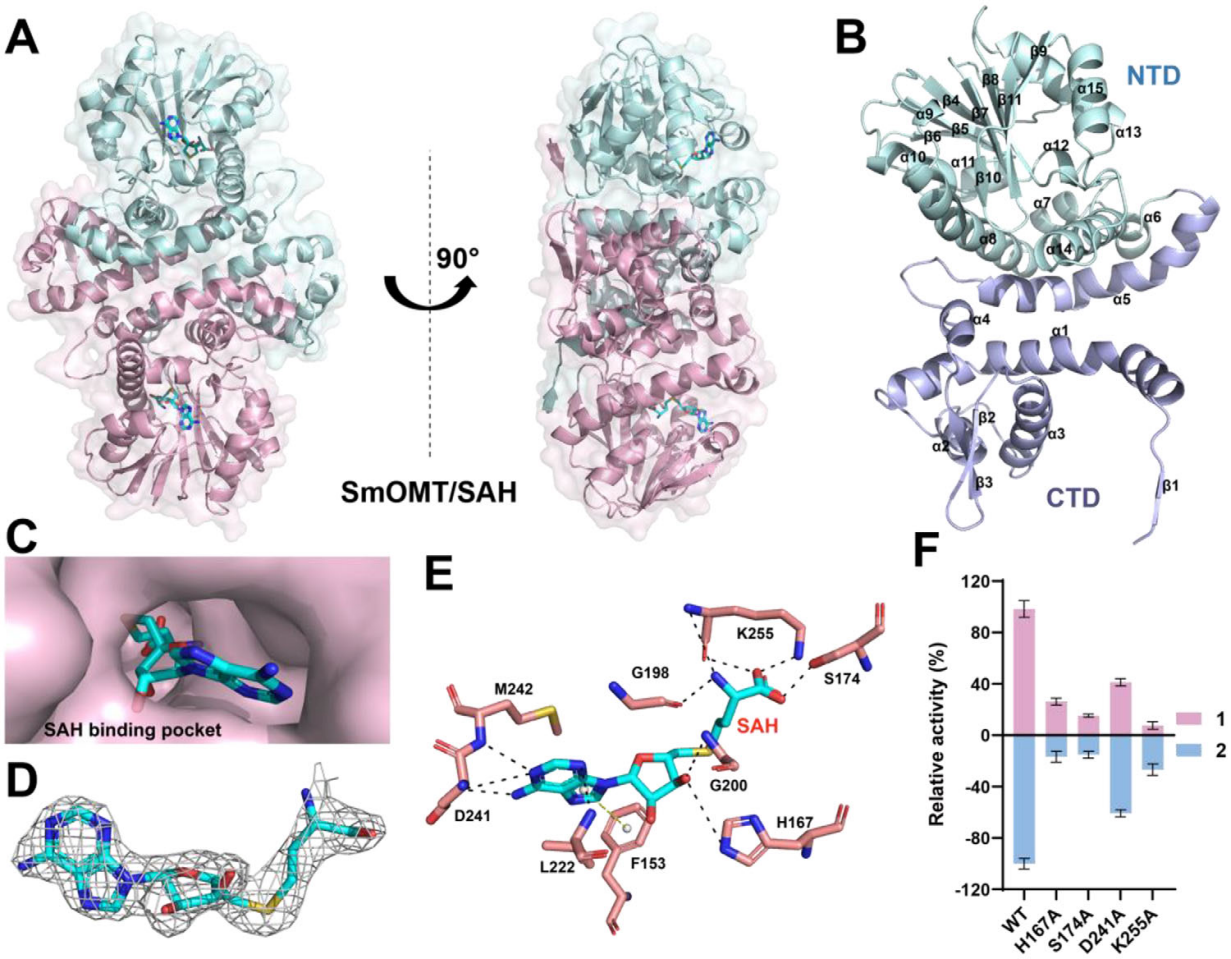
Overall Structure of SmOMT and Its Complex with SAH. (A) Ribbon diagram of the SmOMT homodimer in complex with SAH. The structure was determined at 2.23 Å resolution. Each monomer is colored in light blue and light pink, respectively. (B) Secondary structure of one monomer of the SmOMT–SAH complex, with N‐terminal domain (NTD, light blue) and C‐terminal domain (CTD, light cyan) highlighted. (C) Surface representation of the SAH binding pocket. (D) mFo–DFc omit maps (contoured at 2.0σ) around SAH are shown as a gray mesh. (E) Interactions between SmOMT and SAH. Hydrogen bonds are depicted as black dashed lines; the psi–psi interaction is shown as a gray dashed line. (F) The relative catalytic activities of SmOMT and its mutants were obtained using **1** (purple) and **2** (blue) as the substrates. Data are presented as mean ± SD (n = 3).

To elucidate the structural basis underlying the catalytic mechanism and regioselectivity of SmOMT, the crystal structure of the SmOMT/SAH binary complex (PDB ID: 9WAM) was determined at a resolution of 2.23 Å (Table S2). The overall structure of SmOMT is consistent with that of previously reported plant class I OMTs [40], showing the highest similarity to LpOMT1 from *Lolium perenne* (PDB ID: 3P9I) [32] with 47% sequence identity, a root mean square deviation (RMSD) of 2.2 Å, and a Z‐score of 44.7. SmOMT forms a symmetric homodimer composed of an N‐terminal dimerization and C‐terminal Rossmann‐like fold domains, separated by a layer of α‐helices (Figure 3A). Each monomeric subunit consists of 16 α‐helices and 9 β‐strands, with its overall architecture organized into a smaller N‐terminal dimerization and larger C‐terminal catalytic domains responsible for SAM or SAH and substrate binding (Figure 3A). The dimerization domain is primarily formed by helix α1 (Residues 13–26) and helix α5 (Residues 72–85). Helix α1 inserts into a hydrophobic cleft of the adjacent subunit and forms hydrogen bonds with residues S100, N121, Q122, Q131, R301, S305, and D344 (Figure S68 and Table S3). Helix α5 packs against the adjacent subunit, forming the back wall of the catalytic domain. The dimerization interface exhibits a substantial total buried surface area of 8914.55 Å^2^, indicating strong intersubunit interactions. The presence of this dimerization interface is a common feature among plant class I OMTs and is essential for catalytic activity [41, 42].

In the SmOMT/SAH structure, well‐defined electron density clearly revealed SAH bound within a narrow pocket (Figure 3C,D). This binding pocket was formed by conserved residues (F153, H167, S174, G198, G200, L222, D241, M242, and K255), all located within the characteristic Rossmann‐fold motif (Figure 3E). This motif exhibited the canonical β3‐α12‐β4‐α13‐β5 topology and contained the DXVGXG signature sequence essential for SAM binding [40]. Sequence alignment confirmed high conservation of these residues among plant OMTs (Figure S69). The carboxylate group of SAH formed hydrogen bonds with the Nε atom of Lys255 and the side chain hydroxyl of Ser174 and additional hydrogen bonds with the backbone carbonyl of Lys255 and the main chain amide of Gly206. 2′‐Hydroxyl group of ribose formed a hydrogen bond with the imidazole ring of His167. The adenine ring of SAH is enveloped by Leu222 and Met242 via hydrophobic interactions. A *π*–*π* stacking interaction occurred between the phenyl ring of Phe153 and the adenine ring, while the exocyclic 6‐amino group of adenine formed a hydrogen bond with the carboxylate group of Asp241 (Figure 3E). To validate the functional roles, these residues, H167, S174, D241, and K255, were selected for mutational analysis, followed by enzymatic activity assay using compounds **1** and **2** as substrates. The result demonstrated a consistent and significant reduction in catalytic activity toward all mutants, establishing the critical role of these residues for SmOMT (Figure 3F).

### 2.4 Structural Basis of Substrate Recognition and Catalytic Mechanism in SmOMT

**FIGURE 4:**
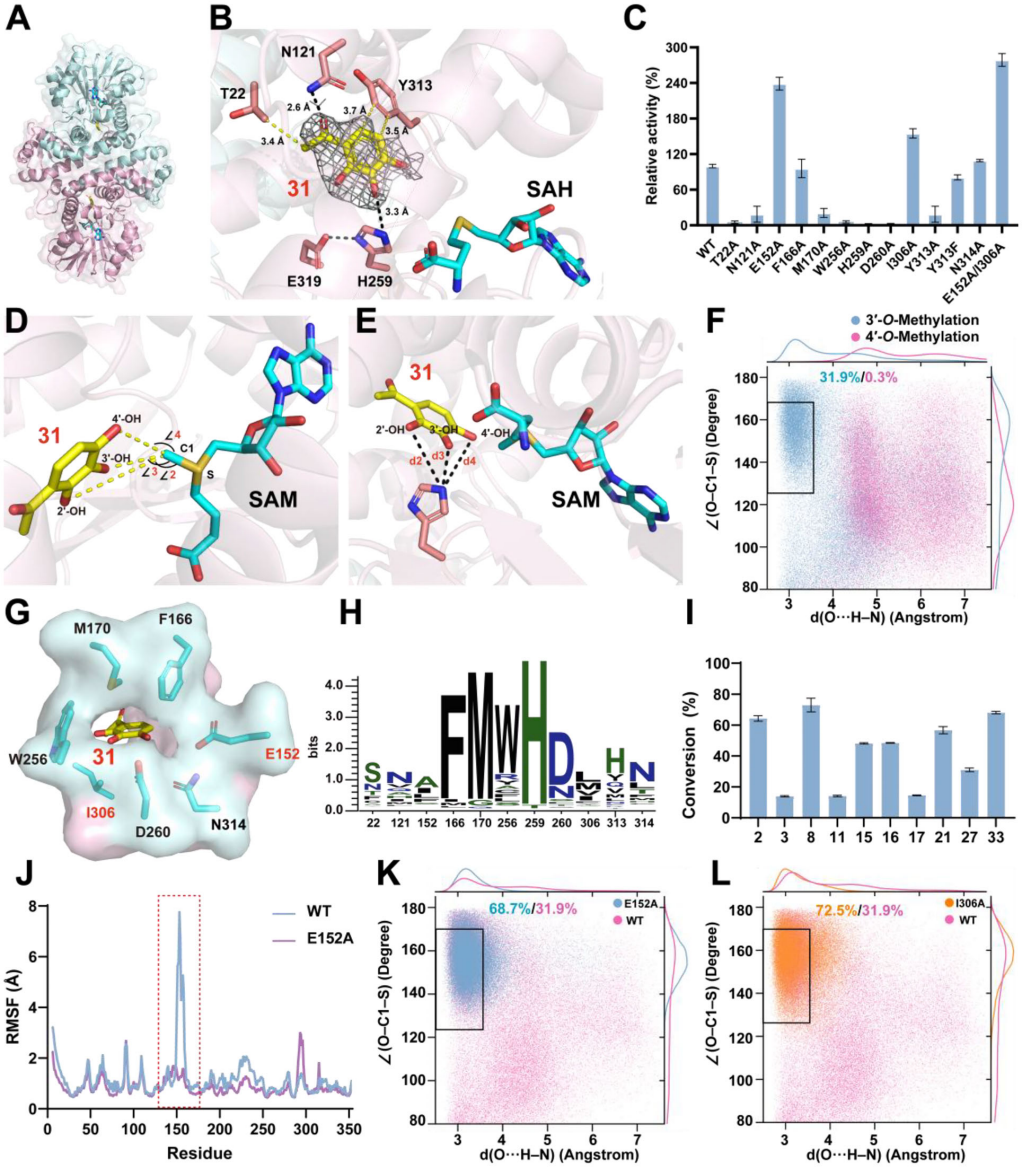
Deciphering the Structural Basis of Substrate Recognition and Regiospecific Catalysis in SmOMT to Engineer Enhanced Activity. (A) Ribbon diagram of the SmOMT homodimer in complex with SAH and gallacetophenone (**31**). The structure was determined at 2.08 Å resolution. (B) Interactions between SmOMT and **31**. mFo–DFc omit maps (contoured at 1.5σ) around **31** are shown as a gray mesh. Hydrogen bonds and hydrophobic interactions are depicted as black and yellow dashed lines, respectively. (C) Relative catalytic activities of the SmOMT mutants using **31** as the substrate. (D) Bond angles ∠(O–C1–S) between hydroxyl oxygen atoms (O2/O3/O4) of **31**, the methyl carbon atom (C1) of SAM, and the sulfur atom (S) of SAH in the SmOMT/SAM/**31** docking model. (E) Distances d(O···H–N) from hydroxyl oxygen atoms (O2/O3/O4) of substrate **31** to the imidazole N–H groups of catalytic residue His259 in the SmOMT/SAM/**31** docking model. (F) Reaction coordinate analysis: Kernel density estimation (KDE) map of catalytic distance [d(O···H–N)] and angle [∠(O–C1–S)] distributions for regioselective methylation (3′ *vs* 4′) of **31**. The proposed methylation‐reactive region (distance < 3.5 Å; angle between 125° and 170°) is outlined by a black square. (G) Top‐view surface representation of SmOMT in complex with **31**. Key residues gating the entrance are shown as sticks. (H) Residue conservation analysis over 150 SmOMT homologous sequences. (I) The conversion rate of SmOMT^M2^ toward other substrates. (J) Per‐residue RMSF analysis of backbone dynamics for wild‐type (WT, blue) and E152A mutant (purple) from MD trajectories. The region of maximal differential flexibility (highlighted by a red dashed box) localizes to the E152 mutation site. (K) Comparative scatter plot of catalytic geometric parameters for WT *vs*. E152A mutant from MD simulations. (L) Comparative scatter plot of catalytic geometric parameters for WT *vs*. I306A mutant from MD simulations. Reactions were performed at 37°C for 12 h. Data are presented as mean ± SD (*n* = 3).

To elucidate the molecular basis of substrate recognition in SmOMT, **1**, **2**, **12**, and **31** (gallacetophenone) were selected as representative ligands for ternary complex structures via co‐crystallization or soaking. Among these ligands, only the SmOMT/SAH/**31** ternary complex (PDB ID: 9WAN) was successfully resolved at a resolution of 2.08 Å (Figure 4A; Table S2), whereas complexes with the other substrates could not be captured. Kinetic analysis revealed that SmOMT exhibits differential substrate affinities, with *K*_m_ values of 176.1 µm (**1**), 585.1 µm (**8**), 225.4 µm (**12**), and 51.7 µm (**31**) (Figure S4). The higher affinity for compound **31** might have facilitated stable complex formation, whereas the low affinity for compounds **1**, **8**, and **12** likely hindered the formation of stable complexes suitable for crystallization. Superimposition of SmOMT/SAH and SmOMT/SAH/**31** structures showed high overall similarity (RMSD = 0.29 Å), with slight changes in several substrate‐binding loops (Figure S70). In the SmOMT/SAH/**31** complex, these loops shifted toward the pocket center, narrowing the entrance and adopting a “closed” state. In contrast, the SmOMT/SAH structure displayed an “open” state with a relatively wider pocket entrance.

Structural analysis revealed that **31** binds near the dimer interface. Hydrophobic interactions occur between the C‐5′ and C‐6′ atoms of compound **31** and Y313. The carbonyl carbon of compound **31** interacts hydrophobically with the side chain of T22. The carbonyl oxygen of compound **31** forms a hydrogen bond with the side chain of N121; the 3′‐OH group forms a hydrogen bond with the side chain of H259 (Figure 4B). Mutagenesis analysis confirmed the functional importance of these residues: Y313A, T22A, N121A, and H259A mutants showed significantly reduced activity (Figure 4C). Additionally, SmOMT was observed to contain a highly conserved His–Glu catalytic dyad, analogous to those reported in plant Class I OMTs, which facilitated proton transfer and charge neutralization [43]. In SmOMT, this dyad was formed by H259 and E319. H259 forms a hydrogen bond not only with the 3′‐OH of compound **31** but also with E319 (Figure 4B). Both H259A and E319A mutants completely lost activity (Figure 4C). These findings underscore the essential role of the H259–E319 dyad in catalysis and suggest that SmOMT likely employs the S_N_2‐like methyl transfer mechanism, consistent with class I OMTs (Figure S71) [29, 30, 31].

### 2.5 Mechanism of Substrate Regiospecificity in SmOMT and Protein Engineering for Enhanced Activity

The regiospecificity of OMTs is primarily governed by two geometric factors: the distance between the substrate nucleophile and the catalytic residue, and the angle formed for nucleophilic attack on the SAM methyl group [43, 44]. To investigate these determinants in SmOMT, a ternary structural model of SmOMT/SAM/**31** was constructed to mimic the reactive state based on the structure of SmOMT/SAH/**31** (Figure S72). Structural analysis revealed distances d(O···H–N of 5.8, 3.3, and 4.0 Å between the 2′‐OH, 3′‐OH, and 4′‐OH groups of **31** and H259, respectively (Figure 3D). The corresponding angles ∠(O–C1–S) formed for attack on the SAM methyl group were 110°, 155°, and 160° (Figure 3E). These values suggested the 3′‐OH was optimally positioned for methylation. To further investigate the dynamic catalytic process, molecular dynamics (MD) simulations were performed. From 20 000 sampled conformations, 31.9% satisfied the catalytic geometry criteria for 3′‐*O*‐methylation (distance <3.5 Å; angle 125°–170°) (Figure 4F) [43, 44]. In contrast, only 0.3% of conformations supported 4′‐*O*‐methylation, and 2′‐*O*‐methylation geometries were exceptionally rare (Figure S73). Furthermore, structural analysis indicated that hydrophobic interactions between residue Y313 and the substrate's C‐5′ and C‐6′ positions are also critical for regioselective 3′‐OH methylation by SmOMT. This finding is strongly supported by the structural features of SmOMT recognizing a series of acetophenone substrates (such as **22**, **24**, **25**, and **32**). In contrast, the presence of substituents at the C‐5′ and C‐6′ positions of the substrate (as in compounds **23**, **26**, **29**, and **30**) significantly impedes effective binding to the SmOMT, leading to a substantial reduction or complete loss of catalytic activity. Mutagenesis experiments showed that the Y313F mutant retained approximately 80% of the relative activity, indicating that the stability of hydrophobic interactions at this site is closely associated with catalytic efficiency (Figure 4C). These results are consistent with both structural analysis and experimental observations, confirming the regiospecificity of SmOMT for 3′‐OH methylation in acetophenone.

To elucidate the regioselectivity of SmOMT toward flavonoids, structural models of the SmOMT/SAH/**2**, SmOMT/SAH/**8**, and SmOMT/SAH/**10** were constructed using docking based on the crystal structure of SmOMT/SAH/**31**, followed by molecular dynamics simulations. The results revealed that for compound **2**, the 3′‐OH and 4′‐OH groups are positioned near the catalytic residue H259 at distances of 2.8 and 5.0 Å, respectively. Conformational sampling analysis from the MD simulations indicated that the 3′‐OH group adopted a methylation‐competent conformation significantly more frequently than the 4′‐OH group (Figure S74A,B). Similarly, for compound **8**, the 3′‐OH and 4′‐OH groups were located at distances of 3.7 and 5.9 Å from H259, respectively. Sampling results showed that the 3′‐OH group exhibited a methylation‐competent conformation in 5.25% of cases, far exceeding the frequency observed for the 4′‐OH group (Figure S74C,D). In contrast, the optimal binding conformation of compound **10** within the active site differed markedly from those of compounds **2** and **8**, with only the 3′‐OH group positioned near H259 at a distance of 2.9 Å. Throughout the MD simulation, the 3‐OH group consistently maintained the most favorable conformation for methylation (Figure S74E,F). These findings provide a structural basis for the preferential methylation of the 3′‐position of flavonoids by SmOMT, as well as its ability to recognize and catalyze methylation at the 3‐OH group in the absence of a 3′‐OH.

To enhance catalytic efficiency, SmOMT was subjected to protein engineering. Alanine‐scanning mutagenesis targeted residues within 5 Å of bound compound **31**, including T22, N121, E152, F166, M170, W256, H259, D260, I306, Y313, and N314 (Figure 4G). Catalytic activity assays revealed that M170A, W256A, and D260A mutants were nearly inactive, and conservation analysis indicated that these residues are highly conserved among plant OMTs, suggesting their essential roles in catalytic activity (Figure 4H). E152A and I306A single mutants exhibited ∼2.3‐ and 1.5‐fold enhancements in activity, respectively, relative to the wild‐type (WT) (Figure 4C). Combining these mutations yielded the double‐mutant SmOMT^E152A/I306A^ (SmOMT^M2^), which demonstrated a 2.6‐fold increase in catalytic activity compared to those in the WT (Figure 4I). Furthermore, the double‐mutant SmOMT^M2^ exhibited enhanced catalytic activity toward other substrates compared to those in the WT. Structural analysis mapped E152 at the entrance of the substrate‐binding pocket, while MD simulations revealed substantial fluctuations of E152 during the trajectory (Figure 4G). The spatial location and flexibility of E152 indicated its potential role in gating substrate entry and product exit. In contrast to WT, the E152A mutant displayed a widened substrate‐binding pocket with reduced conformational fluctuations in MD simulations, indicating that its activity enhancement likely stems from improved efficiency in substrate/product channeling (Figure 4J). Additionally, MD simulations revealed that both the I306A and E152A mutants exhibited increased populations of catalytically competent conformations, suggesting that this conformational shift contributes to their enhanced enzymatic activity (Figure 4K,L).

### 2.6 Coupled Construction of a SAM Regeneration System using SmOMT^M2^ and AtHMT^V140T^

**FIGURE 5:**
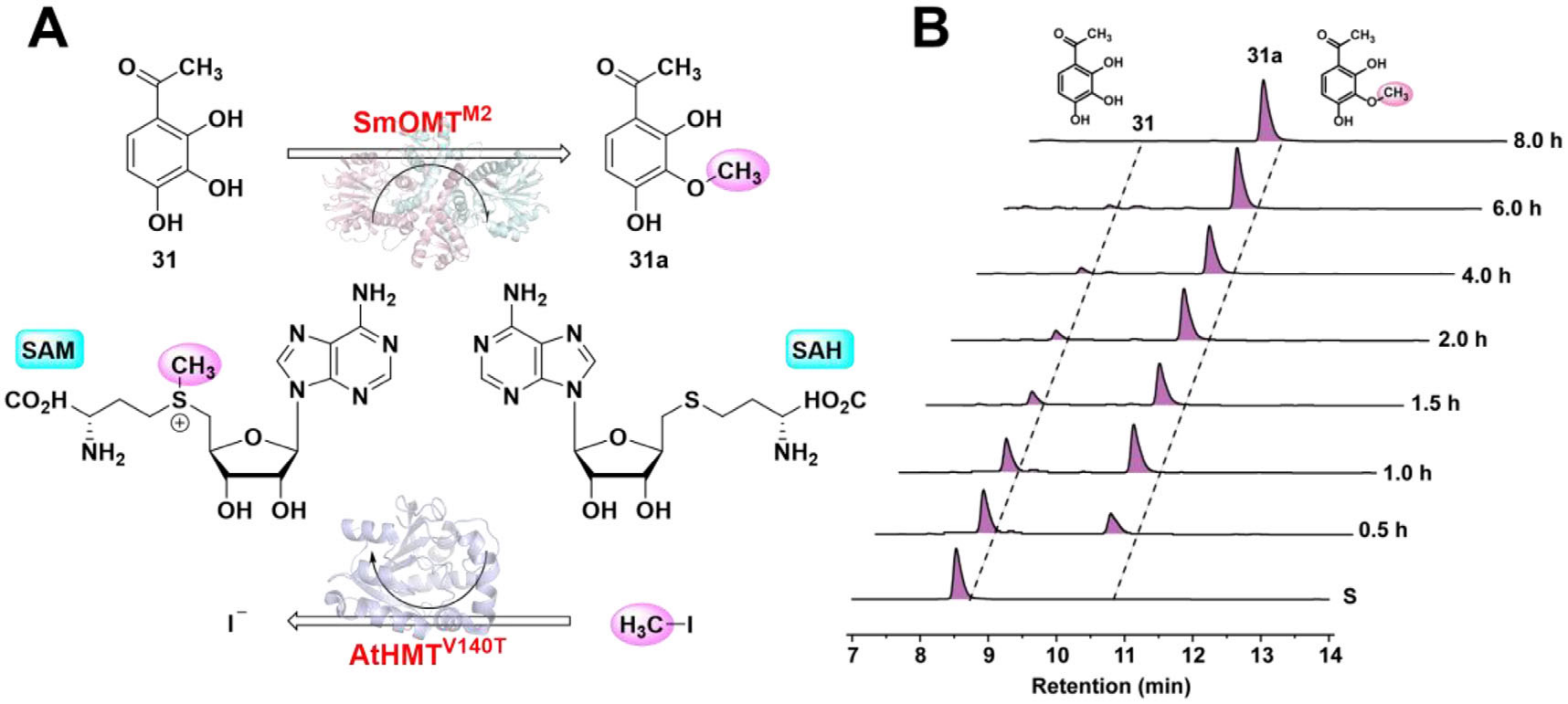
Coupled Construction of a SAM Regeneration System using SmOMT^M2^ and AtHMT^V140T^. (A) Biocatalytic methylation of **31** with SmOMT^M2^ coupled with the SAM recycling system. Reactions were performed at 37°C for 12 h. (B) HPLC chromatograms showing the time course (0.5, 1.0, 1.5, 2.0, 4.0, 6.0, 8.0 h) of the conversion of **31** to methylated product **31a**. Data are presented as mean ± SD (*n* = 3).

Although SmOMT^M2^ with enhanced activity was obtained through mutagenesis, its practical application in biocatalysis remains limited due to its dependence on SAM as a methyl donor. SAM is chemically complex, unstable, and costly, and its reaction byproduct, SAH, is a potent inhibitor of OMTs, posing a great challenge for the in vitro preparation of methylated NPs. *Liao* and *Seebeck* reported the use of a halide methyltransferase, CtHMT from *Chloracidobacterium thermophilum*, to synthesize SAM from SAH and methyl iodide (CH_3_I) [45, 46]. Subsequently, *Tang* obtained the mutant AtHMT^V140T^, derived from *Arabidopsis thaliana* HMT, via directed evolution [47]. This variant demonstrated enhanced catalytic efficiency for synthesizing various SAM analogs. These breakthrough advances laid the foundation for the scalable and specific in vitro methylation of NPs using OMTs [48, 49, 50]. To fully exploit the application potential of SmOMT^M2^, a coupled reaction system integrating AtHMT^V140T^, SmOMT^M2^, SAH, and CH_3_I was implemented. Using **31** as a substrate, AtHMT^V140T^ mediated continuous methylation of SAH (i.e., SAM regeneration) was tested to determine whether it could fulfill the SAM demand of SmOMT^M2^ for methylating **31** (Figure 5A).

To verify whether SAH indeed acts as an inhibitor in the SmOMT^M2^/AtHMT^V140T^ coupled system, different concentration ratios of CH_3_I to SAH (2:1, 1:1, 1:3, 1:5, 1:7, 1:9) were tested to evaluate their impact on the conversion of substrate **31**. The results showed that as the proportion of SAH gradually increased, the conversion of **31** significantly decreased, indicating that excessive SAH exerts an inhibitory effect on the reaction (Figure S75). To further elucidate the inhibitory effect of SAH, the kinetic parameters of substrate **31** were determined under three distinct reaction conditions: 1) the SmOMT + SAM system; 2) the SmOMT + SAM system with an additional 1.5 mm SAH; and 3) the SmOMT^M2^/AtHMT^V140T^ coupled system with an additional 1.5 mm SAH. In the system containing only SmOMT and SAM, a *K*_m_ value of 51.7 µm was obtained (Figure S4). However, the addition of 1.5 mm SAH drastically reduced the enzyme's affinity for the substrate, as reflected by an increase in the *K*_m_ value to 2794 µm. This substantial increase in *K*_m_ confirms the inhibitory effect of SAH, which is consistent with our gradient experiments. In the SmOMT^M2^/AtHMT^V140T^ coupled system with added 1.5 mm SAH, the *K*_m_ value was 499.6 µm (Figure S76). This value is higher than that of the single‐enzyme system without SAH but significantly lower than that of the single‐enzyme system with SAH. This result demonstrates that SAM regeneration alleviates the inhibitory effect of SAH.

Time‐course experiments revealed the progressive methylation of **31**, reaching completion after 8 h (Figure 5B). The reaction time was notably longer than that observed reaction time using SmOMT^M2^ alone with direct SAM supplementation (2 h), potentially due to the time‐consuming process of SAM regeneration. When applied to other substrates, the coupled system yielded lower efficiencies, particularly for low‐conversion substrates (Figure S77). These findings indicate that while SAM regeneration enables sustained methylation, further optimization of regeneration kinetics or process integration is required to achieve scalable biocatalytic methylation of NPs.

### 2.7 Enhancing Methylation Activity of the SAM Regeneration System via an iMARS‐Guided Artificial Fusion Enzyme Strategy

Although the constructed coupled SAM regeneration system overcame the dependency limitation on SAM for SmOMT^M2^, its low catalytic efficiency remained a major bottleneck. The diminished catalytic efficiency of the cascade reaction is primarily attributable to excessive distances between free enzymes, which severely compromise substrate channeling efficiency [51]. Protein fusion represents an effective strategy for constructing artificial multi‐enzyme complexes, as it provides a coordinated spatial organization of enzymes directly, thereby enhancing cascade catalytic activity [52]. Recently, Wang et al. developed iMARS, the first rational design platform for artificial fusion enzymes (FEs) [53]. Based on iMARS, artificial FEs were designed for the SmOMT^M2^/AtHMT^V140T^‐mediated SAM regeneration system to improve methylation efficiency.

**FIGURE 6:**
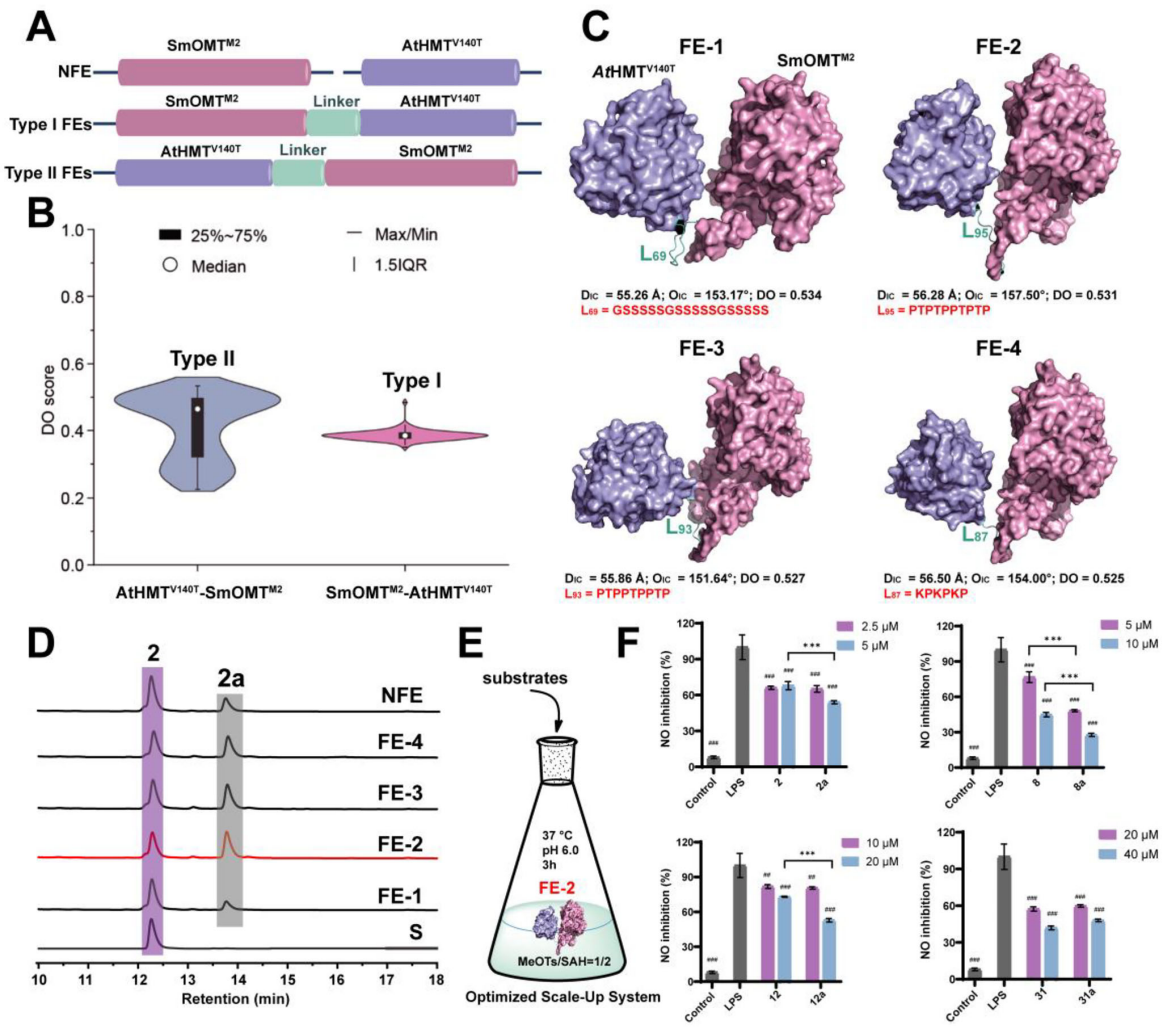
Enhancing Methylation Activity of the SAM Regeneration System via an iMARS‐Guided Artificial Fusion Enzyme Strategy. (A) Architectures of engineered FEs constructed with two fusion orientations and variable linkers. Type I FEs: C‐terminus of SmOMT^M2^ fused to N‐terminus of AtHMT^V140T^; Type II FEs: C‐terminus of AtHMT^V140T^ fused to N‐terminus of SmOMT^M2^. All constructs were expressed in *E. coli* and purified for characterization. (B) Distribution of DO scores across the 1025‐linker library for SmOMT^M2^/AtHMT^V140T^ fusion enzymes, generated using the iMARS strategy. (C) Structures and corresponding DO scores for engineered FEs: AtHMT^V140T^‐(GSSSSS×3)‐SmOMT^M2^, AtHMT^V140T^‐(PTPTP×2)‐SmOMT^M2^, AtHMT^V140T^‐(PTP×3)‐SmOMT^M2^, and AtHMT^V140T^‐(KP×3)‐SmOMT^M2^ FEs. These linkers were selected based on their high DO scores. (D) HPLC profiles of apigenin (**2**) conversion by NFE *vs*. engineered fusion enzymes (FE1–FE4). Reactions were performed at 37°C for 12 h. (E) Schematic of the preparative‐scale methylation reaction catalyzed by engineered FE2 under optimized conditions. (F) Inhibitory effects of compounds **2**/**2a**, **8**/**8a**, **12**/**12a**, and **31**/**31a** on LPS‐induced NO production in RAW 264.7 cells after 48 h treatment. Data are mean ± SD (n ≥ 3). Statistical significance: ^#^ *p* < 0.1, ^##^ *p* < 0.01, ^###^ *p* < 0.001 *vs* LPS‐treated control group; ^*^ *p* < 0.1, ^**^ *p* < 0.01, ^***^ *p* < 0.001 vs respective parent substrate or its methylated product.

Among the factors influencing FEs activity, fusion order and linker design are the most critical. Two fusion configurations for SmOMT^M2^/AtHMT^V140T^ were considered: Type I FEs, with SmOMT^M2^ at the N‐terminal, and Type II FEs, with AtHMT^V140T^ at the N‐terminal (Figure 6A). iMARS predictions indicated that Type II FEs were generally superior (Figure 6B), with the top four ranked FEs (FE‐1, FE‐2, FE‐3, and FE‐4) featuring the linkers: L_69_ (GSSSS×3), L_95_ (PTPTP×2), L_93_ (PTP×3), and L_87_ (KP×3), respectively. The structures of the four FEs were built using AlphaFold3 [54], and two key geometric parameters were measured: D_IC_ (distance between tunnels A and B) and O_IC_ (angle between line segments X and Y) (Figure 6C) [53]. FE‐1 exhibited the smallest D_IC_ (55.26 Å), while FE‐2 exhibited the largest O_IC_ (157.50°). All four FEs demonstrated comparable soluble expression (Figure S78), and catalytic activity profiling using **2** revealed that FE‐1 showed lower activity than the non‐fused enzyme (NFE) control. It also revealed that FE‐2, FE‐3, and FE‐4 exhibited markedly enhanced catalytic activities exceeding those of NFE, with FE‐2 being the most active (Figure 6D). These results demonstrate that L_95_ is the optimal linker for SmOMT^M2^/AtHMT^V140T^ fusion and that higher O_IC_ values positively correlate with catalytic activity. Notably, FE‐2 also demonstrated enhanced activity toward other substrates (Figure S77).

Given the volatility of CH_3_I at 40°C, it was supplied in excess to ensure sustained SAM regeneration. However, CH_3_I is both toxic and capable of alkylating cysteine residues in SmOMT^M2^ and AtHMT^V140T^, thereby impairing catalytic activity [45]. To address this challenge, five less toxic methyl donors—dimethyl sulfate (DMSO), methyl methanesulfonate (MMSa), methyl 4‐nitrobenzenesulfonate (MeONs), methyl benzenesulfonate (MeOBs), and methyl p‐toluenesulfonate (MeOTs)—were systematically evaluated as potential replacements for CH_3_I in the FE‐2 fusion system. Evaluation with substrate **31** indicated that MeOTs, MeOBs, and MeONs were all capable of driving the methylation reaction. Among them, MeOBs achieved a conversion of approximately 50%, while MeOTs and MeONs yielded conversions comparable to that of CH_3_I. Notably, MeOTs, being the least toxic and a solid, presented significantly reduced volatility and associated handling risks compared to liquid donors like CH_3_I (Figure S79). To verify the generality of this methyl donor, a series of substrates (**2**, **12**, **24**, **28**) was tested. In all cases, the target methylated products were successfully detected (Figure S80), confirming that MeOTs can serve as an effective and low‐toxicity alternative to CH_3_I. The FE‐2 system using MeOTs as methyl donors was further optimized, achieving the highest conversion under the following conditions: 37°C, pH 6.0 (50 mm Na_2_HPO_4_/NaH_2_PO_4_), a MeOTs/SAH molar ratio of 2, and a reaction time of 3 h (Figure 6E; Figure S81). To evaluate the potential of the optimized system, scale‐up experiments were conducted. These experiments successfully achieved gram‐scale production of Products **2a**, **8a**, **12a**, and **31a** without the requirement for expensive SAM. Anti‐inflammatory assays demonstrated that the methylated products exhibited stronger NO inhibition compared to their parent compounds (Figure 6F). Notably, product **8a** exhibited enhanced activity with an IC_50_ value of 4.38 µm, showing a 50% reduction relative to compound **8** (IC_50_ = 9.39 µm). These results demonstrate that the artificial FE‐2 not only enables efficient SAM regeneration methylation but also facilitates the synthesis of bioactive derivatives with enhanced therapeutic potential.

## 3 Conclusion

In summary, a plant OMT, SmOMT, was identified from the ancient medicinal plant *S. moellendorffii*. SmOMT exhibits broad substrate promiscuity, catalyzing the methylation of 25 structurally diverse substrates, including flavonoids, coumarins, acetophenones, and anthraquinones, and also demonstrates *N*‐methylation activity. Structural analysis and site‐directed mutagenesis revealed that the H259/E319 catalytic dyad is crucial for SmOMT activity, consistent with an S_N_2‐like methyl transfer mechanism. Alanine scanning revealed that the E152A and I306A mutants exhibit enhanced catalytic activity, while MD simulations indicated that E152 facilitates substrate/product entry and exit from the active site pocket and I306A increases the occurrence frequency of catalytically competent conformations. The double‐mutant SmOMT^M2^ exhibited improved methylation activity and was successfully coupled with the halide methyltransferase variant AtHMT^V140T^ to establish an efficient SAM regeneration cycle system. A high‐activity fusion protein, FE‐2, designed via the iMARS strategy, enabled efficient methylation of multiple substrates without the need for exogenous SAM supplementation. This system facilitated the production of several methylated NPs with markedly enhanced anti‐inflammatory activity. This work effectively overcomes the reliance on costly SAM for enzymatic methylation and delivers a scalable, cost‐effective biocatalytic platform for the synthesis of high‐value methylated compounds.

## 4 Experimental Section

### 4.1 Chemicals and Reagents

Caffeic acid (**1**) and ferulic acid (**1a**) were obtained from Sigma–Aldrich (St. Louis, MO, USA). Flavonoids (**2–11**), coumarins (**12–17**), and resveratrol were sourced from Yuanye Biotechnology (Shanghai, China). Acetophenone derivatives (**18–32**), SAM, and SAH were purchased from Aladdin Biochemical Corporation (Shanghai, China). Crystallization reagents came from Sigma–Aldrich and Hampton Research (Laguna Niguel, CA, USA). HPLC‐grade acetonitrile and methanol were supplied by Merck KGaA (Darmstadt, Germany). DMSO‑*d_6_* was procured from Cambridge Isotope Laboratories (Tewksbury, MA, USA). Phanta Max Super‐Fidelity DNA Polymerase was obtained from Vazyme Biotechnology (Nanjing, China). The MolPure Mag Plant RNA Kit, Hifair II first Strand cDNA Synthesis Kit, DpnI, HindIII, and *Nde*I were purchased from Yeasen Biotechnology (Shanghai, China). All NMR spectra for methylated products were recorded on a Bruker Avance III‐600 NMR spectrometer (Bruker, Billerica, MA, USA). All other chemicals were acquired from Sigma–Aldrich or Aladdin Biochemical Corporation unless otherwise stated.

### 4.2 Candidate Gene Screening and Molecular Cloning

Our laboratory constructed the T2T reference genome of *S. moellendorffii* [55]. We sequenced transcriptomes from roots, stems, and leaves [55]. Candidate genes were screened by local BLAST analysis using TBtools [56]. Phylogenetic trees were generated using the Neighbor‐Joining method in MEGA X [57]. Total RNA was extracted from flash‐frozen *S. moellendorffii* seedlings using the MolPure Mag Plant RNA Kit. Seedlings were ground in liquid nitrogen with a mortar and pestle following the manufacturer′s instructions. First‐strand cDNA was synthesized from total RNA using the Hifair II First‐Strand cDNA Synthesis Kit. Target OMT genes were amplified from cDNA using Phanta Max Super‐Fidelity DNA Polymerase and primers listed in Table S4. PCR products were cloned into the pET28a vector between the NdeI and HindIII sites via homologous recombination. Sangon Biotech (Shanghai, China) sequenced all constructs.

### 4.3 Protein Expression and Purification

The recombinant plasmid pET28a‐SmOMT was transformed into *E. coli* Rosetta (DE3) for heterologous expression. Transformants were selected on LB agar plates containing 50 µg/mL kanamycin. A single colony was inoculated into 80 mL LB medium with 50 µg/mL kanamycin and incubated overnight at 37°C with shaking at 220 rpm. This culture was transferred to 8 L LB medium containing 50 µg/mL kanamycin. Cells were grown at 37°C with shaking at 220 rpm until the OD_600_ reached 0.6–0.8. Protein expression was induced by adding 0.5 mm IPTG. Induction proceeded at 18°C with shaking at 180 rpm for 20 h. Cells were harvested by centrifugation at 6000 rpm for 10 min at 4°C. The pellet was resuspended in 100 mL lysis buffer (20 mm Tris‐HCl, pH 8.0, 300 mm NaCl). Cells were disrupted using an ATS high‐pressure homogenizer (AH‐BASIC 30). The lysate was centrifuged at 12 000 rpm for 45 min at 4°C. The supernatant containing the target protein was collected and loaded onto a Ni‐NTA column (Vazyme, China) pre‐equilibrated with lysis buffer. The target protein was eluted using elution buffer (20 mm Tris‐HCl, pH 8.0, 300 mm NaCl, 300 mm imidazole). Fractions containing the target protein were pooled and concentrated to 2 mL using an Amicon Ultra‐10k centrifugal filter (Merck Millipore). The concentrate was loaded onto a HiPrep 16/60 Sephacryl S‐200HR column (GE Healthcare) equilibrated with 20 mm Tris‐HCl, pH 8.0, containing 150 mm NaCl. Purified protein was concentrated to 10 mg/mL and stored at −80°C for further use.

### 4.4 Biochemical Characterization of SmOMT

Enzymatic properties of SmOMT were characterized by determining its optimal reaction time, pH, temperature, and divalent metal ion dependence. Reaction time was evaluated over 11 time points ranging from 5 to 360 min. For pH optimization, enzymatic assays used four buffer systems: citrate‐sodium citrate (pH 3.0–6.0), Na_2_HPO_4_/NaH_2_PO_4_ (pH 6.0–8.0), Tris‐HCl (pH 6.0–8.0), and Na_2_CO_3_/NaHCO_3_ (pH 9.0–11.0). Temperature dependence was tested between 4°C and 60°C. Metal ion effects were examined using gradient concentrations of Ba^2+^, Fe^2+^, Ca^2+^, Mg^2+^, EDTA, and a blank control. All reactions contained SAM as the methyl donor and caffeic acid (**1**) as the acceptor. Triplicate experiments were performed for each condition. Reactions were terminated by adding methanol, followed by centrifugation at 12 000 rpm for 20 min before HPLC analysis. Kinetic parameters of recombinant SmOMT toward **1**, **8**, **12**, and **31** were determined. The standard mixture contained 50 mm Tris‐HCl‐150 mm NaCl (pH 8.0), 10 µg purified SmOMT, 1.6 mm SAM, and substrates at concentrations ranging from 0.001 to 1 mm. Compound **1** was incubated at 37°C for 2 h. Compounds **8** and **12** were incubated at 37°C for 0.5 h. Compound **31** was incubated at 37°C for 10 min. Reactions were quenched by adding 100 µL methanol. After centrifugation at 12 000 rpm for 20 min, supernatants were analyzed by HPLC. All kinetic assays were performed in triplicate. Enzyme kinetic parameters were calculated using GraphPad Prism 9.0.

### 4.5 Enzyme Activity Assays and Preparative‐Scale Reactions

Substrate promiscuity of SmOMT was investigated using 34 substrates (**1**‐**34**). Enzyme activity assays used 100 µL reaction mixtures containing 0.5 mm substrate, 2 mm SAM, 50 mm Tris‐HCl (pH 8.0), and 100 µg SmOMT. Reactions were incubated at 37°C for 12 h. Termination was achieved by adding 100 µL of methanol. Centrifugation was performed at 12 000 rpm for 20 min. Supernatants were analyzed by HPLC and LC/MS. All reactions were conducted in triplicate. Preparative‐scale reactions (20 mL final volume) contained 50 mm Tris‐HCl (pH 8.0), 1 mm substrate dissolved in DMSO, 2 mm SAM, and 1 mg/mL purified SmOMT. Incubation proceeded at 37°C for 12 h. Reactions were quenched by adding 20 mL of methanol. The mixture was centrifuged at 12 000 g for 20 min. Product purification was performed on a Shimadzu LC‐20AT HPLC system equipped with a reversed‐phase COSMOSIL 5C18‐MS‐II column (250 mm × 10 mm, 5 µm). Structural characterization of purified products was carried out by MS and NMR spectroscopy.

### 4.6 Crystallization and Structure Determination

Purified SmOMT (10 mg/mL) was incubated with 5 mm SAH at 4°C for 30 min. Crystallization was performed by hanging‐drop vapor diffusion at 18°C. Drops contained 0.8 µL protein solution mixed with 0.8 µL reservoir solution, which was 0.1 m Bis‐Tris pH 5.5 with 25% (w/v) PEG 3350. SmOMT1/SAH crystals appeared within 2–3 days. To obtain ternary complexes, SmOMT1/SAH crystals were soaked in crystallization solution containing 2.5 mm ligand for exposure times of 15 min, 30 min, 1 h, or 2 h. All crystals were harvested directly from the reservoir solution. Cryoprotection was achieved by adding 20% (v/v) glycerol before flash‐freezing the crystals in liquid nitrogen. X‐ray diffraction data were collected at beamline BL19U1 of the Shanghai Synchrotron Radiation Facility (SSRF) [58]. Diffraction images were processed with XDS [59]. The SmOMT/SAH structure was solved by molecular replacement using Phenix [60]. An AlphaFold3 predicted SmOMT model served as the search model [54]. Initial model building used AutoBuild, followed by manual refinement in Coot [61, 62]. Iterative refinement and validation were performed with Phenix. Protein‐ligand interactions were analyzed using the Protein‐Ligand Interaction Profiler (PLIP) [63]. Structural visualization used PyMOL [64]. The SmOMT1/SAH/**31** complex structure was determined using the same protocol.

### 4.7 Molecular Dynamics Simulations

The crystal structure of the SmOMT/SAH/**31** complex served as the initial model. To better simulate the methyl transfer reaction, we replaced SAH with SAM as the methyl donor in our modeling system. We introduced two mutations (E152A and I306A) using PyMOL. Parameterization of wild‐type and mutant complexes utilized the Antechamber module in AmberTools [65]. Protein atoms were assigned parameters from the Amber14SB force field [66]. Ligand parameters employed the General Amber Force Field (GAFF) [67]. Each complex was solvated in a TIP3P explicit water box. Molecular dynamics simulations were executed with NAMD 2 [68]. Production runs of 100 ns were performed for each system. Trajectory analysis used the MD Analysis package [69]. Visualization was implemented with the Seaborn package [70].

### 4.8 Site‐Directed Mutagenesis

Site‐directed mutagenesis of SmOMT was performed using pET28a‐SmOMT as the template and mutagenic primers (Table S4). Purified PCR products were digested with DpnI. The digested products were transformed into *E. coli* DH5α competent cells. Mutant plasmids were confirmed by DNA sequencing. Verified plasmids were transformed into *E. coli Rosetta* (DE3) for heterologous protein expression.

### 4.9 SmOMT‐AtHMT Coupled Reaction

The pET28a‐AtHMT construct was generated as described previously. Catalytic activity of the SmOMT‐AtHMT combination was evaluated using compound **31** as substrate. Reactions were performed in 100 µL mixtures containing 50 mm Tris‐HCl‐150 mm NaCl buffer (pH 8.0), 10 µg purified SmOMT, 10 µg purified AtHMT, 0.8 mm substrate, 4 mm SAH, and 10 mm CH_3_I. After12 h incubation at 37°C, reactions were quenched by adding 100 µL methanol. Centrifugation was performed at 12 000 rpm for 20 min. Supernatants were analyzed by HPLC and LC/MS. Reaction progress was monitored by HPLC at 7 time points between 0.5 and 8 h.

### 4.10 Construction of Fusion Enzymes and Reaction System Optimization

Fusion enzymes were constructed by PCR amplification using specific primers (Table S5) with AtHMT and SmOMT as templates. Fusion plasmids were generated through multi‐fragment homologous recombination and verified by DNA sequencing. The four fusion enzymes were expressed and purified as described previously for SmOMT. Optimization of the SAM‐independent reaction system catalyzed by fusion enzymes was performed using luteolin as substrate. Four key parameters were investigated: the molar ratio of CH_3_I to SAH (tested at 5:1, 4:1, 3:1, 2:1, 1:1, 1:2), reaction time (5–420 min), pH (3.0–11.0), and temperature (4°C –60°C). Reactions were terminated prior to HPLC analysis. All optimization assays were conducted in triplicate.

### 4.11 HPLC and LC‐MS Analysis

HPLC analysis used a Thermo Fisher Ultimate 3000 system (USA). Separation was performed on an XSelect HSS T3 column (5 µm, 250 × 4.6 mm) at a flow rate of 0.8 mL/min. The mobile phase consisted of (A) acetonitrile and (B) deionized water containing 0.1% formic acid. The gradient program was: 0–20 min (10%–100% A), 20–25 min (100% A), 25–28 min (100%–10% A), 28–35 min (10% A). Methylated products were confirmed by LTQ XL Orbitrap mass spectrometry (Thermo Fisher Scientific Inc.). MS analysis employed positive ion mode with 35% normalized collision energy.

### 4.12 Anti‐Inflammatory Activity Analysis

RAW264.7 cells were incubated with substrates or methylated products (2.5–60 µm) at 37°C for 48 h. Experimental groups included LPS‐treated (1.2 µg/mL) and untreated controls. Culture supernatants were subsequently discarded. Nitric oxide (NO) accumulation in cell‐free supernatants was measured using the Griess reaction to evaluate inducible nitric oxide synthase (iNOS) activity [35].

## Author Contributions

X.X., J.L., and W.H. conceived the study and designed experiments. X.X., J.S., and S.L. conducted experimental investigations. X.X., J.L., S.L., and W.H. analyzed data and interpreted results. X.X. and J.S. wrote and prepared the manuscript with critical input from S.Y., Y.C., Y.Y., and X.L. X.L., J.L., and W.H. supervised the project, acquired funding, and finalized the manuscript.

## Conflicts of Interest

The authors declare no conflicts of interest.

## Supporting information




**Supporting File**: advs73412‐sup‐0001‐SuppMat.pdf.

## Data Availability

Coordinates have been deposited in the Protein Data Bank (www.rcsb.org) under accession numbers: 8JJN (SmOMT/SAH) and 8K5U (SmOMT/SAH/**31**), respectively. Source data are provided with this paper.
