# Supplementary material for: Structure‐Guided Engineering of a Promiscuous O‐Methyltransferase for a SAM Regeneration Biocatalysis Platform of Methylated Pharmaceuticals
Source: Adv Sci (Weinh). 2025 Dec 19;13(13):e17794. doi: 10.1002/advs.202517794 (PMC12955871; doi:10.1002/advs.202517794)
Supplement: Supplementary file 1 — Supporting File: advs73412‐sup‐0001‐SuppMat.pdf. [file ADVS-13-e17794-s001.pdf]

Supplementary Information for

Structure-Guided Engineering of a Promiscuous *O*-Methyltransferase for a SAM Regeneration Biocatalysis Platform of Methylated Pharmaceuticals

Xiran Xiong[1#], Jun Song[1.2#], Shihan Li[1#], Lu Jin[1.2], Qi He[1], Baohui Zhang[1.2], Yan Cao[1.2], Shanyong Yi[3], Yanfang Yang[1.2], Xiang Li[4], Juan Li*[1.2], Wei Huang *[1.2]*

1. School of Pharmacy, Hubei University of Chinese Medicine, Wuhan 430065, China

2. Hubei Shizhen Laboratory, Hubei University of Chinese Medicine, Wuhan 430065, China

3. Traditional Chinese Medicine Institute of Anhui Dabie Mountain, West Anhui University, Luan 237012, China

4. Department of neurosurgery, Zhongnan Hospital of Wuhan University, Wuhan 430071, China

[#]These authors contributed equally: Xiran Xiong, Jun Song, Shihan Li

*Correspondence: huangwei19920910@163.com, lz198207@126.com, li.xiang@whu.edu.cn

Table of content

^{1}H and ^{13}C NMR spectral data for methylated products. 1
Table 6
Supplementary Table 1. The catalytic activity of three candidate OMTs using three structurally distinct NPs as substrates. 6
Supplementary Table 2. Data collection and refinement statistics of SmOMT/SAH and SmOMT/SAH/**31**. 7
Supplementary Table 3. Detailed information on amino acid residues forming hydrogen bond interactions with helix α1. 8
Supplementary Table 4. Primers used for candidate gene cloning and site-directed mutagenesis in this study. 9
Supplementary Table 5. Primers used for fusion enzyme construction in this study. 10
Figure 11
Supplementary Fig 1. SDS-PAGE analysis of purified proteins from three candidate genes after Ni-NTA affinity purification 11
Supplementary Fig 2. Purification of the recombinant protein SmOMT.. 11
Supplementary Fig 3. The biochemical characterizations of SmOMT. 12
Supplementary Fig 4. Determination of kinetic parameters for SmOMT. 13
Supplementary Fig 5-25. HPLC and LC-MS analysis of the products catalyzed by SmOMT using **3-34** as the substrate. 14
Supplementary Fig 26-67. NMR spectrum of methylated products 35
Supplementary Fig 68. Amino acid residues forming hydrogen bonds with helix α1.. 57
Supplementary Fig 69. Protein sequence alignment of SmOMT with other OMTs.. 57
Supplementary Fig 70. The structure superimposition between SmOMT/SAH and SmOMT/SAH/**31**. 58
Supplementary Fig 71. Proposed catalytic mechanism of SmOMT. 58
Supplementary Fig 72. The ternary structural model of SmOMT/SAM/**31.** 59
Supplementary Fig 73. KDE mapping of catalytic distance and angle distributions for 2′-*O* methylation of substrate **31**. 59
Supplementary Fig 74. Regioselectivity of SmOMT toward flavonoids**.** 60
Supplementary Fig 75. Evaluation of SAH as an inhibitor in the coupled system.. 60
Supplementary Fig 76. Kinetic analysis of substrate **31** under different reaction conditions 61
Supplementary Fig 77. Catalytic activity of the NFE and FE2 for six substrates. 61
Supplementary Fig 78. SDS-PAGE analysis of the Ni-NTA affinity purification and Size exclusion chromatography profile of four FEs. 62
Supplementary Fig 79. HPLC analysis shows the conversion of substrate **31** using the SmOMT^{M2}/AtHMT^{V140T} fusion system with five candidate methyl donors.. 61
Supplementary Fig 80. Substrate scope of methylation using MeOTs as the methyl donor. HPLC analysis confirms the conversion of substrates **2**, **12**, **24**, and **28**. 63
Supplementary Fig 81. Optimization of the SAM-Independent methylation system catalyzed by engineered FE-2.. 63

^{1}H and ^{13}C NMR spectral data for methylated products

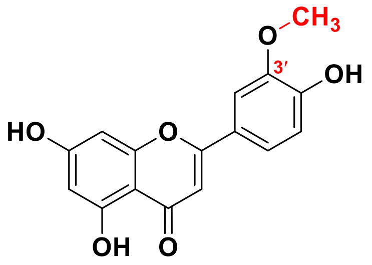

Chrysoeriol (2a):^{1}H-NMR (DMSO-d_6, 600MHz): δ 12.97 (s, 1H, 5-OH), 7.56 (m, 1H, H-6′), 7.55 (s, 1H, H-2′), 6.94 (d, J = 8.9, 1H, H-5′), 6.90 (s, 1H, H-3), 6.51 (d, J = 2.0 Hz, 1H, H-8), 6.19 (d, J = 2.1 Hz, 1H, H-6), 3.89 (s, 3H, 3′-OCH_3).

^{13}C-NMR (DMSO-d_6, 151 MHz): δ 181.8 (C-4), 164.1 (C-7), 163.7 (C-2), 161.4 (C-5), 157.3 (C-9), 148.0 (C-3′), 150.7 (C-4′), 121.5 (C-1′), 120.3 (C-6′), 115.7 (C-5′), 110.2 (C-2′), 103.2 (C-10), 103.7 (C-3), 98.8 (C-6), 94.0 (C-8), 55.9 (3′-O$\underline{C}H_3$).

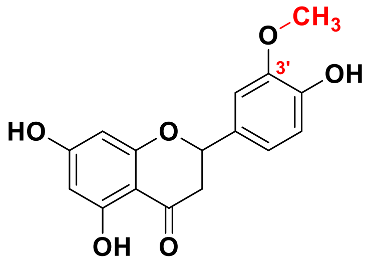

Homoeriodictyol (6a):^{1}H NMR (DMSO-d_6, 600 MHz) δ 12.15 (s, 1H, 5-OH), 7.09 (d, J = 2.0 Hz, 1H, H-2′), 6.90 (dd, J = 8.1, 2.0 Hz, 1H, H-5′), 6.79 (d, J = 8.1 Hz, 1H, H-6′), 5.89 (d, J = 2.1 Hz, 1H, H-8), 5.88 (d, J = 2.2 Hz, 1H, H-6), 5.43 (dd, J = 12.9, 2.9 Hz, 1H, H-2), 3.79 (s, 3H, 3′-OCH_3), 3.31 (d, J = 13.0 Hz, 1H, H-3), 2.68 (dd, J = 17.1, 3.0 Hz, 1H, H-3).

^{13}C NMR (DMSO-d_6, 151 MHz) δ 196.4 (C-4), 166.6 (C-7), 163.5 (C-5), 162.9 (C-9), 147.5 (C-3′), 147.0 (C-4′), 129.4 (C-1′), 119.7 (C-6′), 115.2 (C-5′), 111.2 (C-2′), 101.7 (C-10), 95.8 (C-6), 95.0 (C-8), 78.7 (C-2), 55.7 (3′-OCH_3), 42.1 (C-3).

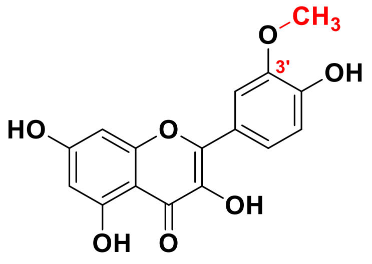

Isorhamnetin (**8a**):^{1}H NMR (DMSO-d_6, 600 MHz) δ 12.46 (s, 1H, 5-OH), 7.76 (d, J = 2.1 Hz, 1H, H-2′), 7.70 (dd, J = 8.4, 2.1 Hz, 1H, H-6′), 6.95 (d, J = 8.5 Hz, 1H, H-5′), 6.48 (d, J = 2.0 Hz, 1H, H-6), 6.19 (d, J = 2.1 Hz, 1H, H-8), 3.84 (s, 3H, 3′-OCH3).

^{13}C NMR (DMSO-d_6, 151 MHz) δ 175.9 (C-4), 163.9 (C-7), 160.7 (C-5), 156.2 (C-9), 148.8 (C-4′), 147.4 (C-3′), 146.6 (C-2), 135.8 (C-3), 122.0 (C-1′), 121.7 (C-6′), 115.5 (C-5′), 111.7 (C-2′), 103.0 (C-10), 98.2 (C-6), 93.6 (C-8), 55.8 (3′-OCH3).

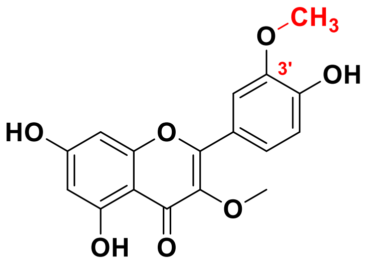

3,3′-Dimethylquercetin (**9a**):^{1}H NMR (DMSO-d_6, 600 MHz) δ 13.09 (s, 1H,5-OH), 7.57 (m, 1H, H-6′), 7.55 (s, 1H, H-5′), 6.94 (m, 1H, H-2′), 6.90 (s, 1H, H-6), 6.62 (s, 1H, H-8), 3.89 (s, 3H, 3′-OCH3), 3.75 (s, 3H, 3-OCH3).

^{13}C NMR (DMSO-d_6, 151 MHz) δ 182.2 (C-4), 163.7 (C-7), 157.2 (C-2), 152.7 (C-5), 156.2 (C-9), 150.7 (C-4′), 148.0 (C-3′), 131.3 (C-1′), 121.5 (C-6′), 120.3 (C-5′), 115.7 (C-3), 110.2 (C-2′), 104.1 (C-10), 102.7 (C-6), 94.3 (C-8), 59.9 (3-OCH3), 56.0 (3′-OCH3).

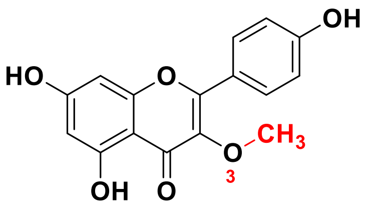

Isokaempferide (**10a**):^{1}H NMR (DMSO-d_6, 600 MHz) δ 12.68 (s, 1H, 5-OH), 7.94 (d, J = 2.0 Hz, 2H, H-2′, H-6′), 6.95 (d, J = 2.0 Hz, 2H, H-3′, H-5′), 6.43 (d, J = 2.0 Hz, 1H, H-6), 6.20 (d, J = 2.0 Hz, 1H, H-8), 3.78 (s, 3H, 3-OCH3).

^{13}C NMR (DMSO-d_6, 151 MHz) δ 177.9 (C-4), 164.1 (C-7), 161.2 (C-5), 160.2 (C-9), 156.4 (C-4′), 155.6 (C-2), 137.6 (C-3), 130.1 (C-2′), 120.6 (C-1′), 115.6 (C-3′), 104.2 (C-10), 98.6 (C-6), 93.7 (C-8), 59.7 (3-OCH3).

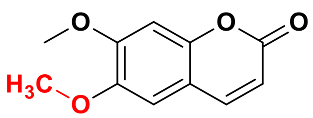

Scoparone (15a): ^{1}H NMR (DMSO-d_6, 600 MHz): δ 7.92 (s, 1H, H-4), 7.22 (s, 1H, H-5), 7.04 (s, 1H, H-8), 6.28 (d, J = 9.4 Hz, 1H, H-3), 3.85 (s, 3H, 6-OCH_3), 3.80 (s, 3H, 7-OCH_3).

^{13}C NMR (DMSO-d_6, 151 MHz): δ 160.6(C-2), 152.5 (C-7), 149.4 (C-9), 145.8 (C-6), 144.3 (C-4), 112.62 (C-3), 111.2 (C-10), 108.9 (C-5), 100.0 (C-8), 56.1 (7-OCH_3), 55.9 (6-OCH_3).

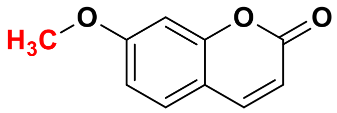

7-Methoxycoumarin (16a): ^{1}H NMR (DMSO-d_6, 600 MHz) δ 7.96 (dd, J = 9.6, 0.6 Hz, 1H, H-4), 7.60 (d, J = 8.5 Hz, 1H, H-5), 6.96 (d, J = 2.4 Hz, 1H, H-8), 6.92 (dd, J = 8.6, 2.5 Hz, 1H, H-6), 6.27 (d, J = 9.5 Hz, 1H, H-3), 3.84 (s, 3H, 7-OCH_3).

^{13}C NMR (DMSO-d_6, 151 MHz) δ 162.4 (C-2), 160.3 (C-7), 155.4 (C-9), 144.3 (C-4), 129.4 (C-5), 112.4 (C-3), 112.3 (C-10), 112.3 (C-6), 100.7 (C-8), 55.9 (7-OCH_3).

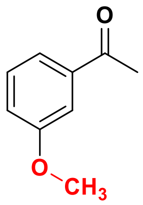

3-Methoxyacetophenone (19a): ^{1}H NMR (DMSO-d_6, 600 MHz) δ 7.55 (dt, J = 7.7, 1.1 Hz, 1H, H-6), 7.46 – 7.43 (m, 1H, H-2), 7.43 (dd, J = 2.9, 1.2 Hz, 1H, H-5), 7.21 (ddd, J = 8.2, 2.7, 1.0 Hz, 1H, H-4), 3.81 (s, 3H, 3-OCH_3), 2.57 (s, 3H, -$COCH_3$).

^{13}C NMR (DMSO-d_6, 151 MHz) δ 197.7 (-$\underline{C}OCH_3$), 159.4 (C-3′), 138.2 (C-1′), 129.8 (C-5′), 120.8 (C-6′), 119.1 (C-4′), 112.6 (C-2′), 55.3 (3-OCH_3), 26.8 (-$CO\underline{C}H_3$).

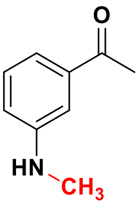

1-[3-(methylamino)phenyl]ethenone (21a): ^{1}H NMR (DMSO-d_6, 600 MHz) δ 7.22 (t, J = 7.8 Hz, 1H, H-6), 7.14 (ddd, J = 7.6, 1.7, 1.0 Hz, 1H, H-5), 7.06 (dd, J = 2.4, 1.7 Hz, 1H, H-2), 6.78 (ddd, J = 8.1, 2.5, 1.0 Hz, 1H, H-4), 5.90 (q, J = 5.0 Hz, 1H, 3-NH-), 2.70 (d, J = 5.1 Hz, 3H, 3-$NHCH_3$), 2.51 (s, 3H, -$COCH_3$).

^{13}C NMR (151 MHz, DMSO-d_6) δ 198.3 (-$\underline{C}OCH_3$), 150.0 (C-3′), 137.6 (C-1′), 129.1 (C-5′), 116.4 (C-6′), 115.8 (C-4′), 110.1 (C-2′), 29.6 (3-$NHCH_3$), 26.7 (-$CO\underline{C}H_3$).

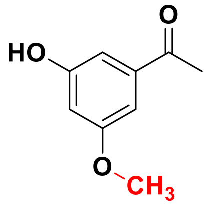

5′-Hydroxy-3′-Methoxyacetophenone (**24a**):^{1}H NMR (DMSO-d_6, 600 MHz) δ 9.96 (s, 1H, 4-OH), 7.51 (dd, J = 8.3, 2.0 Hz, 1H, H-6′), 7.44 (d, J = 2.0 Hz, 1H, H-2′), 6.86 (d, J = 8.2 Hz, 1H, H-5′), 3.82 (s, 3H, 3′-OCH_3), 2.49 (s, 3H, -$COCH_3$).

^{13}C NMR (DMSO-d_6, 151 MHz) δ 196.1 (-$\underline{C}OCH_3$), 151.7 (C-4′), 147.5(C-3′), 128.8 (C-1′), 123.4 (C-6′), 114.9 (C-5′), 111.1 (C-2′), 55.6 (3-OCH_3), 26.2 (-$CO\underline{C}H_3$).

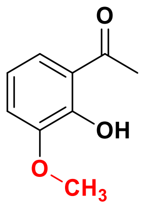

1-(2-hydroxy-3-methoxy-phenyl)ethenone (25a): ^{1}H NMR (600 MHz, DMSO-d_6) δ 12.06 (d, J = 0.5 Hz, 1H, 2-OH), 7.45 (dd, J = 8.1, 1.4 Hz, 1H, H-6), 7.22 (dd, J = 8.0, 1.4 Hz, 1H, H-5), 6.88 (t, J = 8.0 Hz, 1H, H-4), 3.80 (s, 3H, 3-OCH_3), 2.63 (s, 3H, -$COCH_3$).

^{13}C NMR (DMSO-d_6, 151 MHz) δ 205.0 (-$\underline{C}OCH_3$), 151.3 (C-2′), 148.3 (C-3′), 122.2 (C-6′), 120.2 (C-1′), 118.4 (C-5′), 117.5 (C-4′), 55.9 (3-OCH_3), 27.8 (-$CO\underline{C}H_3$).

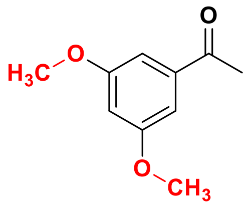

3′,5′-Dimethoxyacetophenone (28b): ^{1}H NMR (DMSO-d_6, 600 MHz) δ 7.06 (d, J = 2.3 Hz, 2H, H-2, H-6), 6.75 (t, J = 2.3 Hz, 1H, H-4), 3.80 (s, 6H, 3-OCH_3, 5-OCH_3), 2.55 (s, 3H, -$COCH_3$).

^{13}C NMR (151 MHz, DMSO-d_6) δ 197.6 (-$\underline{C}OCH_3$), 160.5 (C-3′, C-5′), 138.8 (C-1′), 105.9 (C-2′, C-6′), 105.0 (C-4′), 55.5 (3-OCH_3, 5-OCH_3), 26.8 (-$CO\underline{C}H_3$).

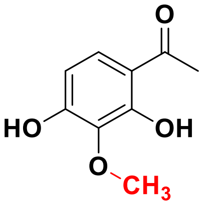

2′,4′-Dihydroxy-3′-methoxyacetophenone (**31a**):^{1}H NMR (DMSO-d_6, 600 MHz) δ 12.25 (d, J = 0.8 Hz, 1H, 2′-OH), 8.64 (s, 1H, 4′-OH), 7.44 (dd, J = 9.0, 1.0 Hz, 1H, C-6′), 6.64 (d, J = 8.9 Hz, 1H, C-5′), 3.85 (s, 3H, 3′-OCH_3), 2.57 (s, 3H, -$COCH_3$).

^{13}C NMR (DMSO-d_6, 151 MHz) δ 203.9 (-$\underline{C}OCH_3$), 153.3 (C-2′), 150.9 (C-4′), 133.6 (C-3′), 122.7 (C-6′), 114.6 (C-1′), 103.4 (C-5′), 55.9 (3-OCH_3), 26.7 (-$CO\underline{C}H_3$).

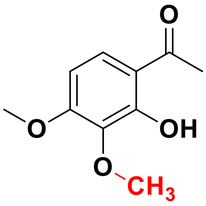

2′-Dihydroxy-3′,4′-methoxyacetophenone (**32a**):^{1}H NMR (DMSO-d_6, 600 MHz) δ 12.38 (s, 1H, 2′-OH), 7.71 (d, J = 9.0 Hz, 1H, H-6′), 6.69 (d, J = 9.0 Hz, 1H, H-5′), 3.88 (s, 3H, 4′-OCH_3), 3.69 (s, 3H, 3′-OCH_3), 2.57 (s, 3H, -$COCH_3$).

^{13}C NMR (DMSO-d_6, 151 MHz) δ 203.8 (-$\underline{C}OCH_3$), 158.3 (C-4′), 155.8 (C-2′), 135.6 (C-3′), 127.8 (C-6′), 115.0 (C-1′), 103.7 (C-5′), 59.9 (3′-OCH_3), 56.1 (4′-OCH_3), 26.8 (-$CO\underline{C}H_3$).

Table

Supplementary Table 1. The catalytic activity of three candidate OMTs using three structurally distinct NPs as substrates.

	SmoeChr8G00150560.1	SmoeChr1G00007300.1	SmoeChr7G00139050.1
Caffeic acid (**1**)	+	-	+
Luteolin (**2**)	+	-	-
Xanthotoxol (**12**)	+	+	-

"+" detected product; "-" no product

Supplementary Table 2. Data collection and refinement statistics of SmOMT/SAH and SmOMT/SAH/**31**.

	**SmOMT/SAH**	**SmOMT/SAH/31**
**PDB Entry**	9WAM	9WAN
Space group	$P2_12_12_1$	$P2_12_12_1$
a, *b*, *c* (Å)	84.21, 95.41, 97.34	64.165 67.018 154.145
α, β, γ (°)	90, 90, 90	90, 90, 90
Resolution (Å)	31.58 - 2.23 (2.31 - 2.23)	46.35 - 2.08 (2.15 - 2.08)
Unique reflections	392924 (13712)	459166 (47115)
Redundancy	10.3 (4.0)	11.4 (12.0)
Completeness (%)	98.35 (89.29)	98.43 (97.81)
I/σ(I)	25.71 (2.76)	23.10 (6.79)
R_{merge}	0.04 (0.46)	0.11 (0.72)
$CC_{1/2}$	1 (0.822)	0.996 (0.920)
$R_{\mathrm{work}}/R_{free}$	0.19/0.23	0.19/0.24
**No. atoms**		
Protein	5409	5410
Ligands	52	76
***B*-factor (Å^2)**		
Protein	52.04	22.26
Ligands	20.67	35.81
Bond length (Å)	0.15	0.14
Bond angle (°)	2.24	2.12
Favored (%)	98.27	98.41
Allowed (%)	1.45	1.16
Outliers (%)	0.29	0.43

Supplementary Table 3. Detailed information on amino acid residues forming hydrogen bond interactions with helix α1.

A Chain	Atom	B Chain	Atom	Distance (Å)
14R	NH2	344D	OD1	3.3
14R	NH2	344D	OD2	3.4
14R	NE	344D	OD2	2.9
13D	OD1	103K	NZ	3.5
13D	OD2	100S	OG	2.9
21A	O	121N	ND2	3.0
21A	O	122Q	NE2	3.2
19E	OE1	301R	NH2	2.9
19E	OE2	301R	NE	2.9
25S	OG	305S	OG	3.0
31K	NZ	131Q	OE1	2.9

Supplementary Table 4. Primers used for candidate gene cloning and site-directed mutagenesis in this study.

Name	Prime Sequence
*Sm*OMT-F	GTGCCGCGCGGCAGCCATATGATGGGGTCTGCCGGGGGA
*Sm*OMT-R	CTCGAGTGCGGCCGCAAGCTTTCACTTGTGGAACTCCACTACTGATA
*At*HMT-F	CCGCGCGGCAGCCATATGGCGGAAGAACAGCAGAACTC
*At*HMT-R	TGCGGCCGCAAGCTTTCAGTTGATCTTTTTCCAACGACCC
T22A-F	GTGATCGTGGAGGATGCAGATCGCCTCCAGATA
T22A-R	CATTATCTGGAGGCGATTGCCATCCTCCACGATCA
N121A-F	CATCATGTCCTCATGGCACAAGACAAGGTGTTCA
N121A-R	CATGAACACCTTGTCTTGTGCCATGAGGACATGAT
E152A-F	GCGTTTGGGCAGACCGCATTTGAGCTGGGAAAG
E152A-R	CTTTCCCAGCTCAAATGCGGTCTGCCCAAACGC
F166A-F	AGGGTCAACAATCTCGCACACGCCGCCATGTCC
F166A-R	GGACATGGCGGCGTGTGCGAGATTGTTGACCCT
H167A-F	TCAACAATCTCTTCGCAGCCGCCATGTCCAATC
H167A-R	ATTGGACATGGCGGCTGCGAAGAGATTGTTGAC
M170A-F	GTCTCTCTGGCGCATGCAGTCCTCATGAATCAA
M170A-R	TTGATTCATGAGGACTGCATGCGCCAGAGAGAC
S174A-F	GCCATGTCCAATCACGCAAAGCTCTACATGAAC
S174A-R	GTTCATGTAGAGCTTTGCGTGATTGGACATGGC
D241A-F	GAGCACGTTGGCGGGGCAATGTTTGTGAGCGTC
D241A-R	GACGCTCACAAACATTGCCCCGCCAACGTGCTC
K255A-F	GCCATCTTCATGGCATGGATCCTGCACGACTGG
K255A-R	GTCGTGCAGGATCCATGCCATGAAGATGGCGTC
W256A-F	GCCATCTTCATGAAGGCAATCCTGCACGACTGG
W256A-R	CCAGTCGTGCAGGATTGCCTTCATGAAGATGGC
H259A-F	ATGAAGTGGATCCTGGCAGACTGGAGCGATGAG
H259A-R	CTCATCGCTCCAGTCTGCCAGGATCCACTTCAT
D260A-F	AAGTGGATCCTGCACGCATGGAGCGATGAGGCT
D260A-R	AGCCTCATCGCTCCATGCGTGCAGGATCCACTT
I306A-F	CGAGTGGCTCTCAGCGCAGATCTCTTGATGCTC
I306A-R	GAGCATCAAGAGATCTGCGCTGAGAGCCACTCG
Y313A-F	CATCGATCTCTTGATGCTCGTCGCAAATCCGGGTGGCA
Y313A-R	TCTCCTTGCCACCCGGATTTGCGACGAGCATCAAGAGA
N314A-F	TTGATGCTCGTCTATGCACCGGGTGGCAAGGAG
N314A-R	CTCCTTGCCACCCGGTGCATAGACGAGCATCAA
E319A-F	CTATAATCCGGGTGGCAAGGCAAGGACGTTTGAGGATTTT
E319A-R	CAAAATCCTCAAACGTCCTTGCCTTGCCACCCGGATTATA

Supplementary Table 5. Primers used for fusion enzyme construction in this study.

Name	Primer sequence
F1	GGTGCCGCGCGGCAGCCATATGATGGCGGAAGAACAGCAGAACTCTG
FE-1-R1	ACCACTACTACTACTACTACCGCTGCTGCTGCTGCTACCGTTGATCTTTTTCCAACGAC
FE-1-F2	GTAGTAGTAGTAGTGGTTCTTCTTCTTCTTCTGGGTCTGCCGGGGGAGTGATCGT
FE-2-R1	CGGGGTCGGCGTCGGCGGTGTCGGGGTCGGGTTGATCTTTTTCCAACGACCCAGTTTTT
FE-2-F2	CACCGCCGACGCCGACCCCGGGGTCTGCCGGGGGAGTGATCGTGGA
FE-3-R1	CGGCGTCGGCGGTGTCGGCGGGGTCGGGTTGATCTTTTTCCAACGACCCAGTTTTTCTT
FE-3-F2	CGCCGACACCGCCGACGCCGGGGTCTGCCGGGGGAGTGAT
FE-4-R1	CGGTTTCGGCTTCGGTTTGTTGATCTTTTTCCAACGACCCAGTT
FE-4-F2	AAACCGAAGCCGAAACCGGGGTCTGCCGGGGGAGTGATCGT
R2	CTCGAGTGCGGCCGCAAGCTTTCACTTGTGGAACTCCACTACTGAT

Figure

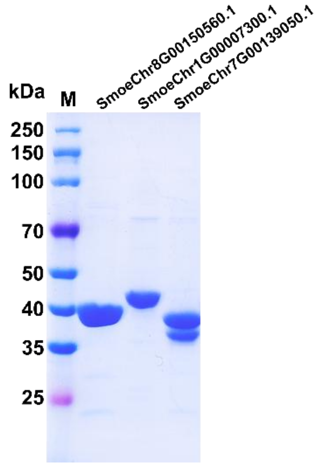

Supplementary Fig 1. SDS-PAGE analysis of purified proteins from three candidate genes after Ni-NTA affinity purification

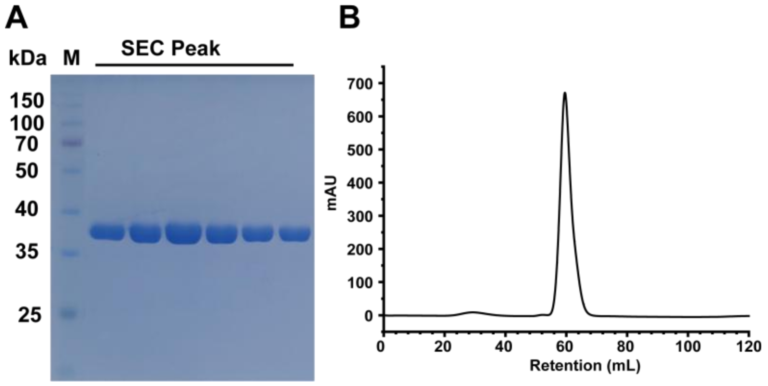

Supplementary Fig 2. Purification of the recombinant protein SmOMT. **A)** SDS-PAGE analysis of the purified SmOMT protein. **B)** Size exclusion chromatography profile of the SmOMT.

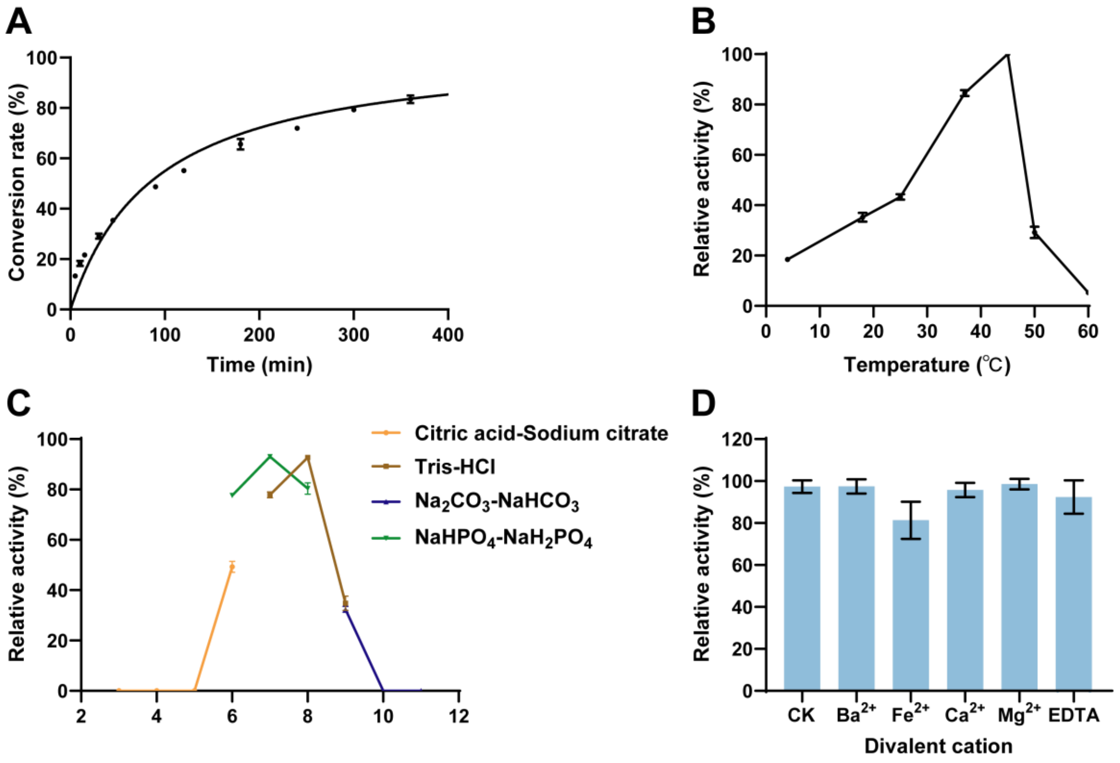

Supplementary Fig 3. The biochemical characterizations of SmOMT.

A) Time. **B)** temperature. **C)** pH. **D)** divalent metal ions. CK represents the control reaction without adding metal ions.

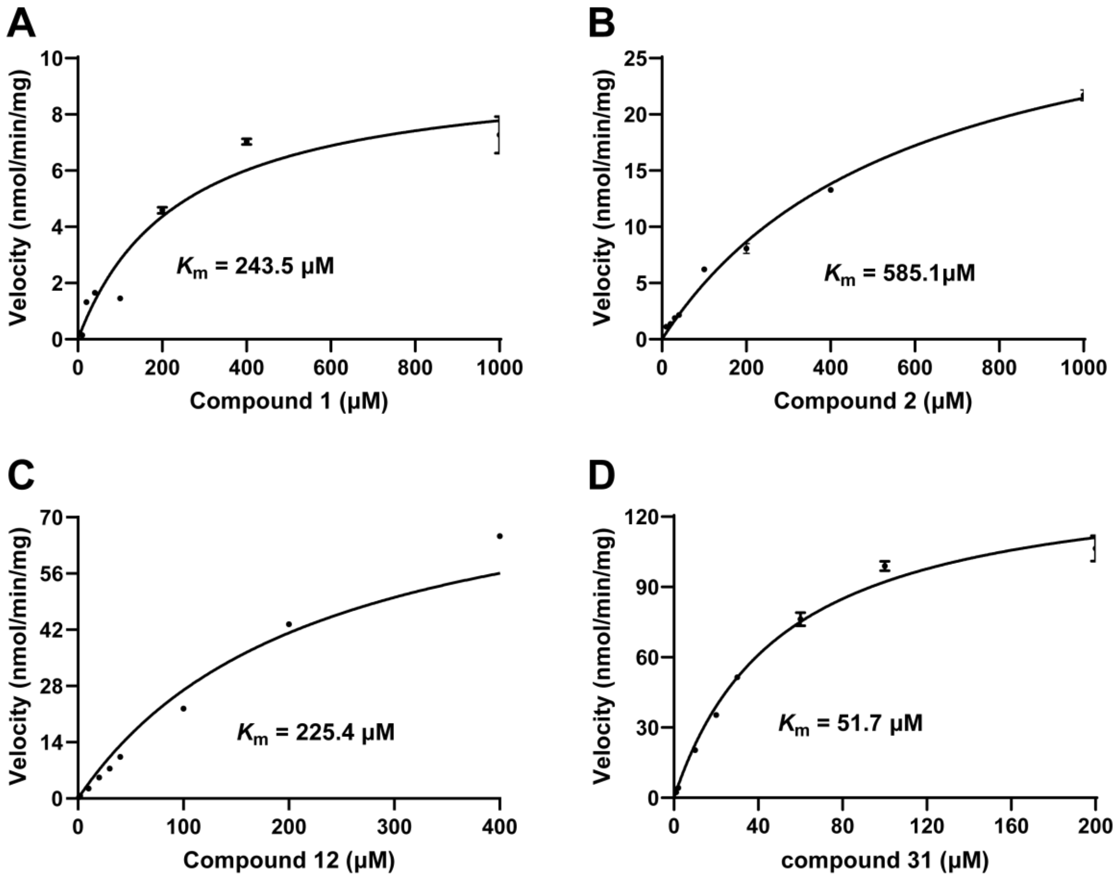

Supplementary Fig 4. Determination of kinetic parameters for SmOMT.
The experiments were performed using caffeic acid **1** (**a**), luteolin **2** (**b**), xanthotoxol **12** (**c**), and gallacetophenone **31** (d) as substrates.

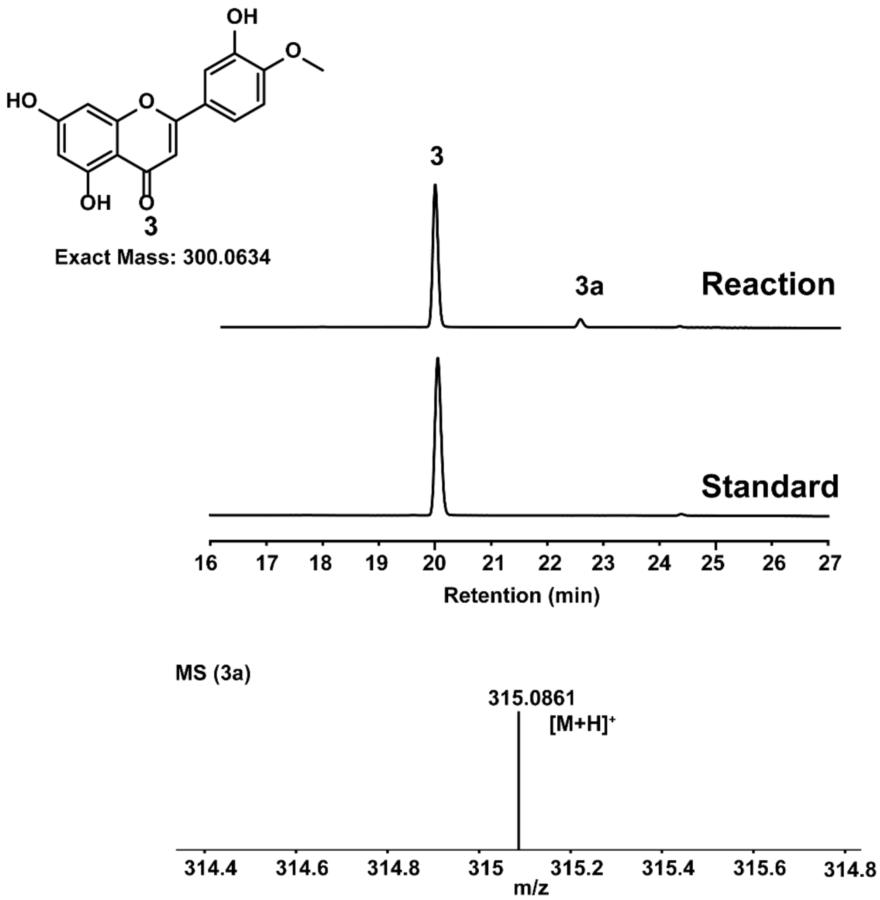

Supplementary Fig 5. HPLC and LC-MS analysis of the products catalyzed by SmOMT using **3** as the substrate.

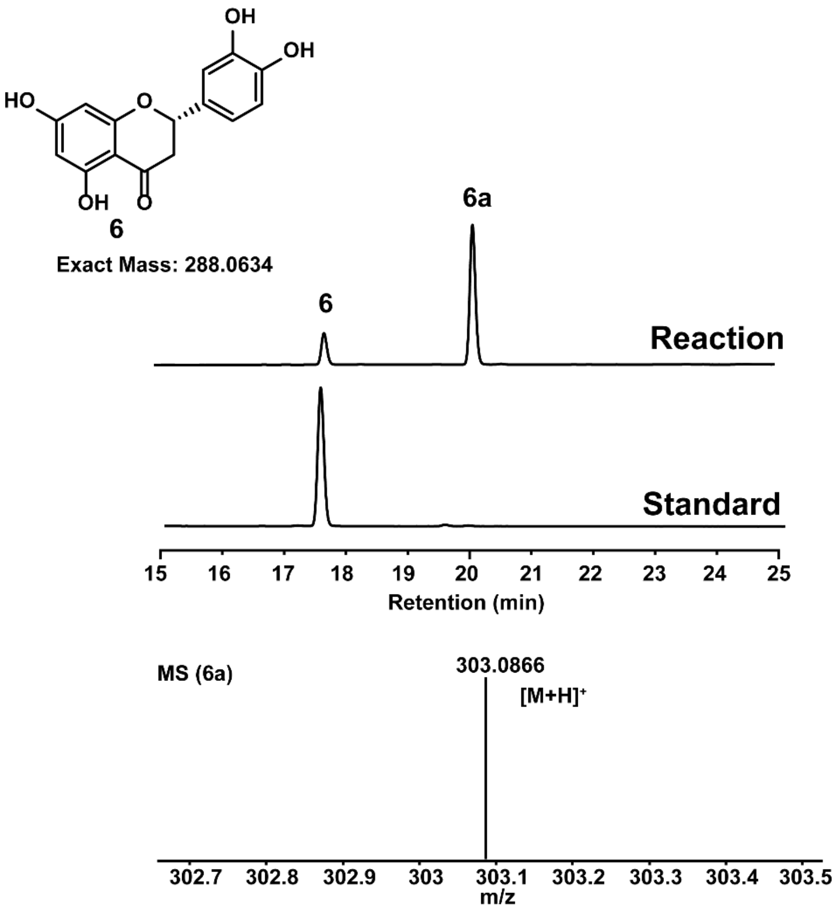

Supplementary Fig 6. HPLC and LC-MS analysis of the products catalyzed by SmOMT using **6** as the substrate.

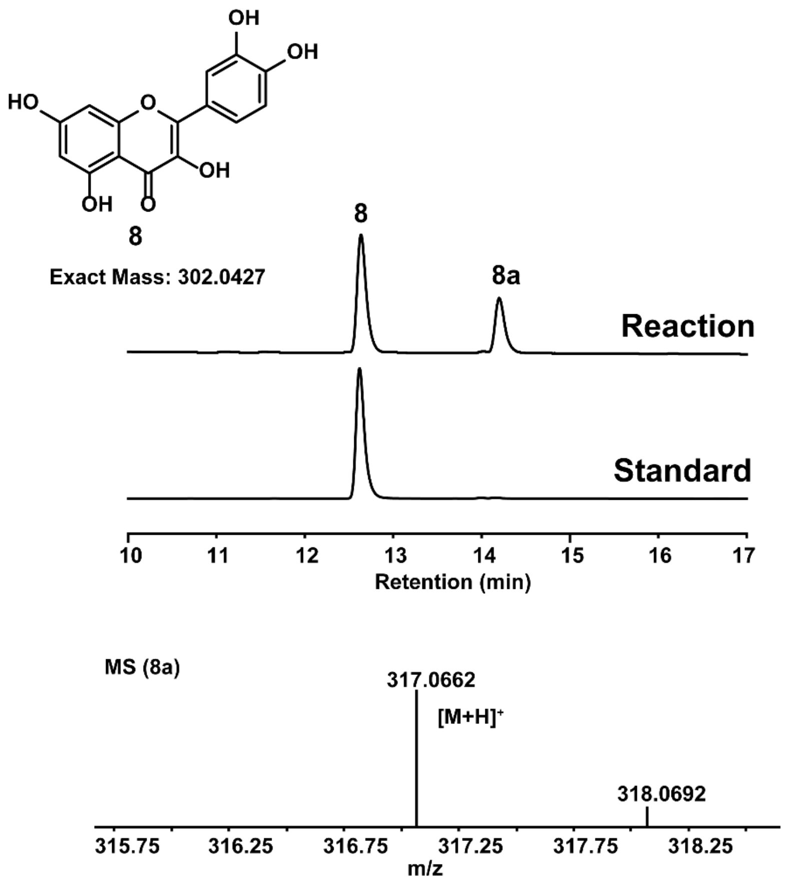

Supplementary Fig 7. HPLC and LC-MS analysis of the products catalyzed by SmOMT using **8** as the substrate.

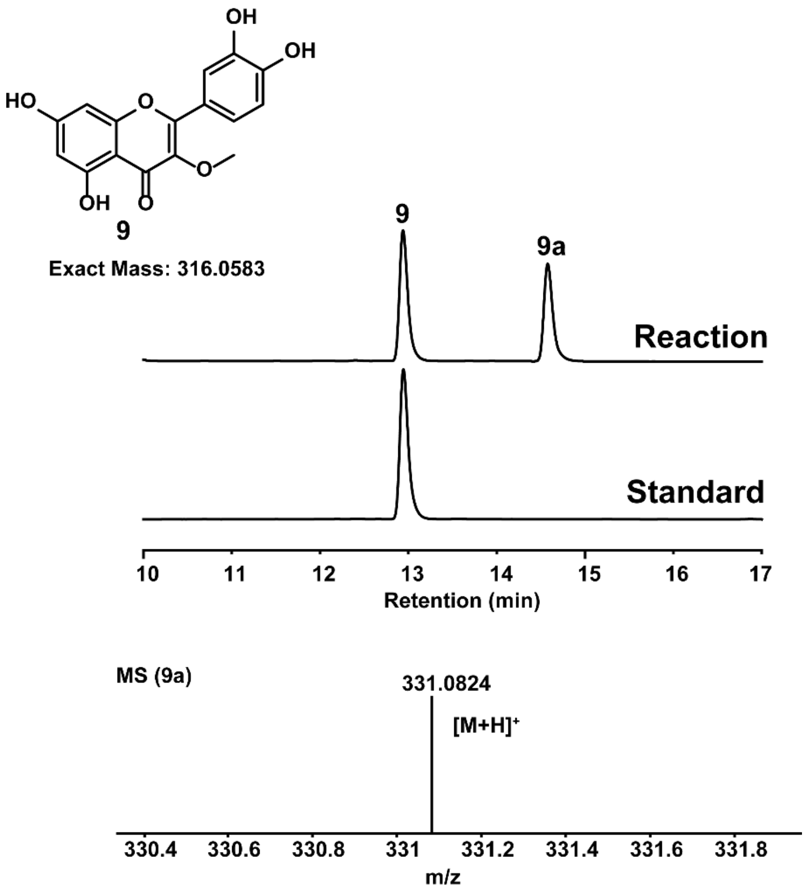

Supplementary Fig 8. HPLC and LC-MS analysis of the products catalyzed by SmOMT using **9** as the substrate.

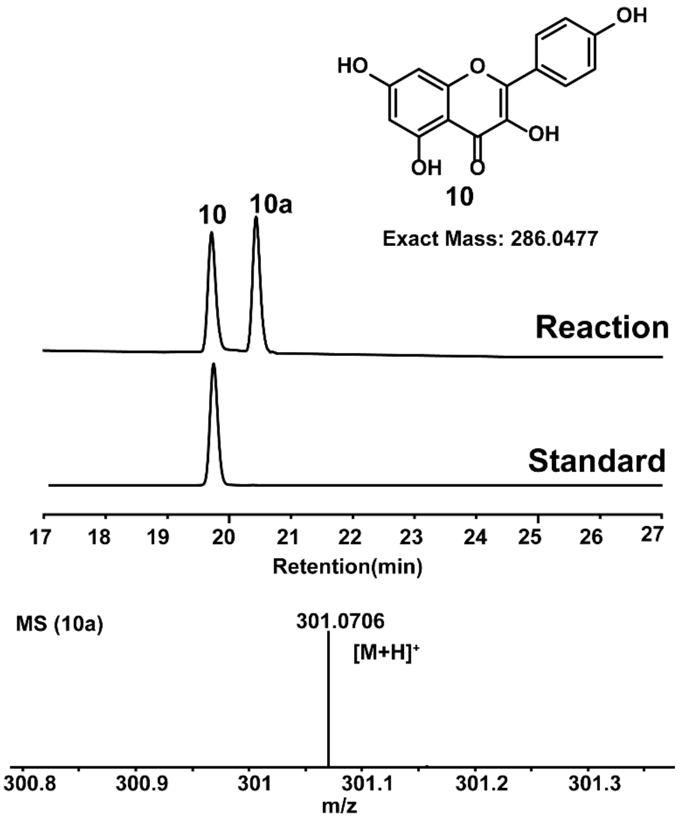

Supplementary Fig 9. HPLC and LC-MS analysis of the products catalyzed by SmOMT using **10** as the substrate.

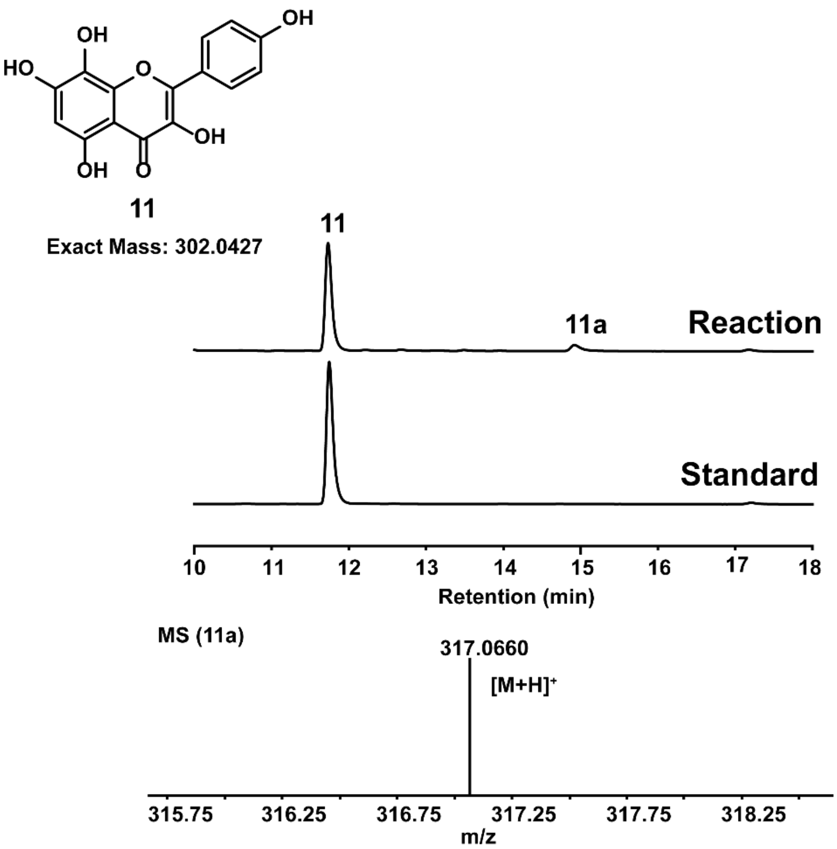

Supplementary Fig 10. HPLC and LC-MS analysis of the products catalyzed by SmOMT using **11** as the substrate.

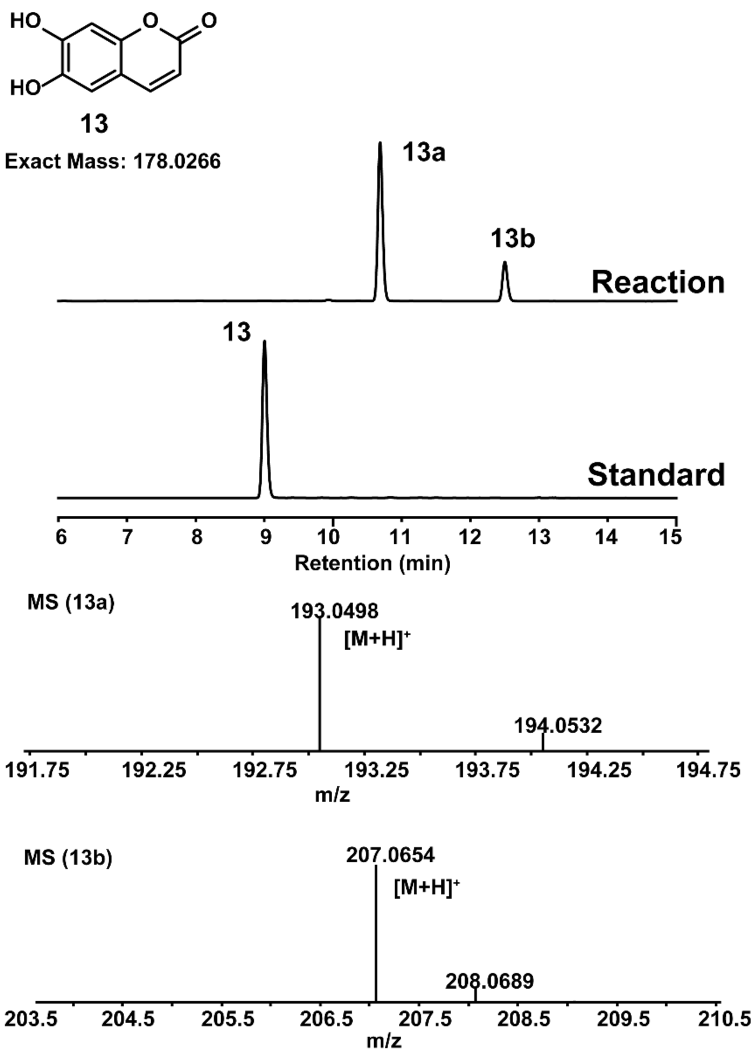

Supplementary Fig 11. HPLC and LC-MS analysis of the products catalyzed by SmOMT using **13** as the substrate.

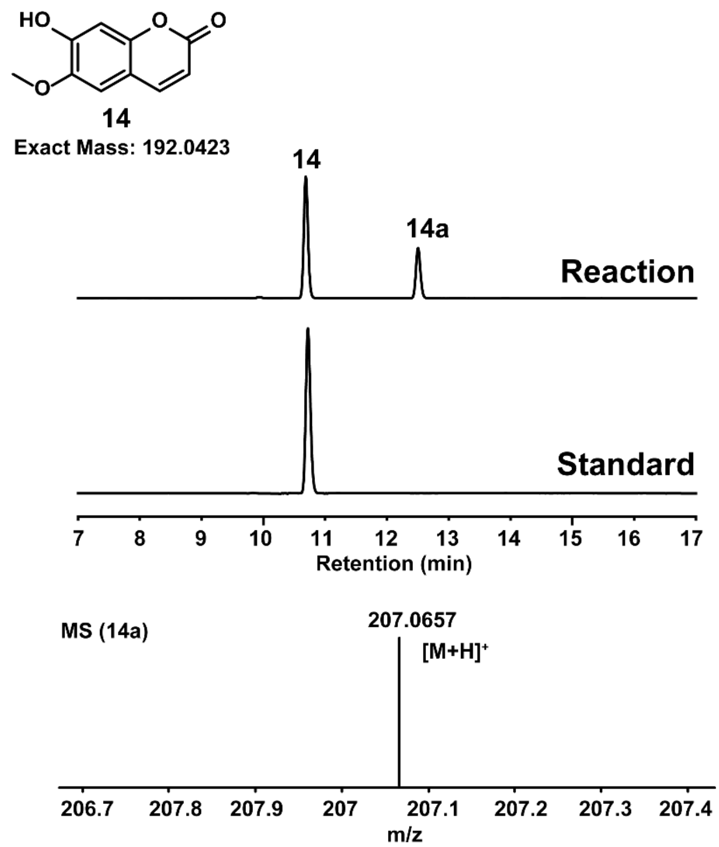

Supplementary Fig 12. HPLC and LC-MS analysis of the products catalyzed by SmOMT using **14** as the substrate.

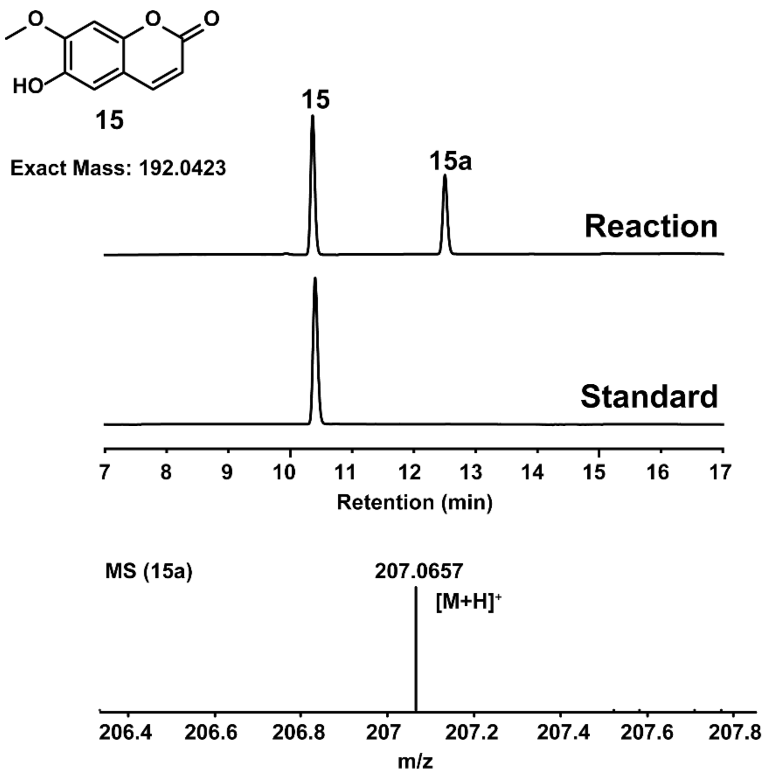

Supplementary Fig 13. HPLC and LC-MS analysis of the products catalyzed by SmOMT using **15** as the substrate.

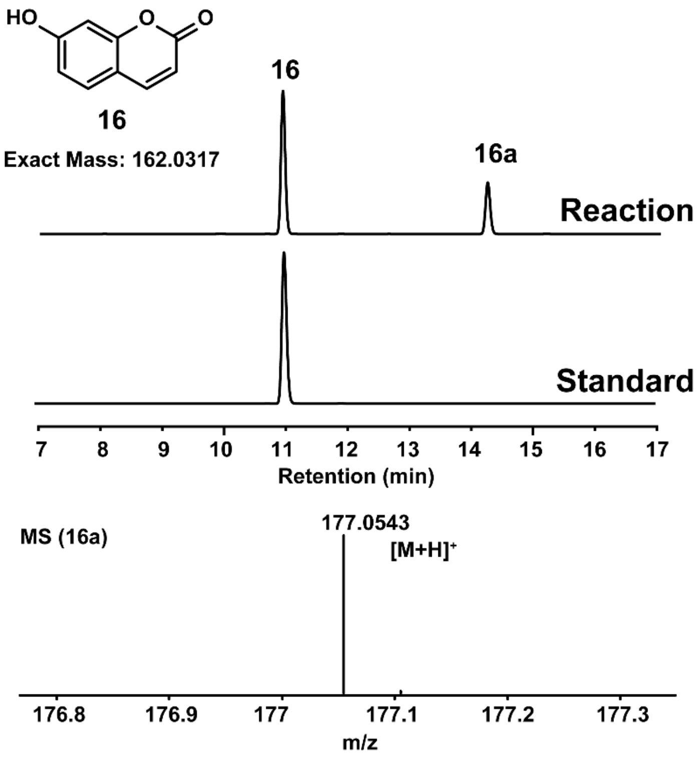

Supplementary Fig 14. HPLC and LC-MS analysis of the products catalyzed by SmOMT using **16** as the substrate.

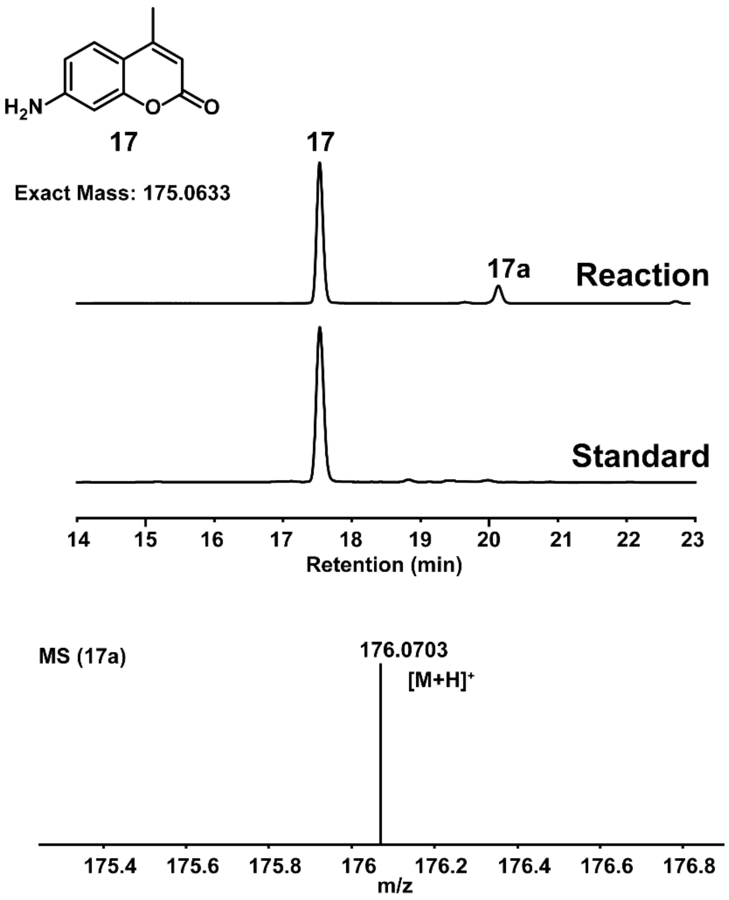

Supplementary Fig 15. HPLC and LC-MS analysis of the products catalyzed by SmOMT using **17** as the substrate.

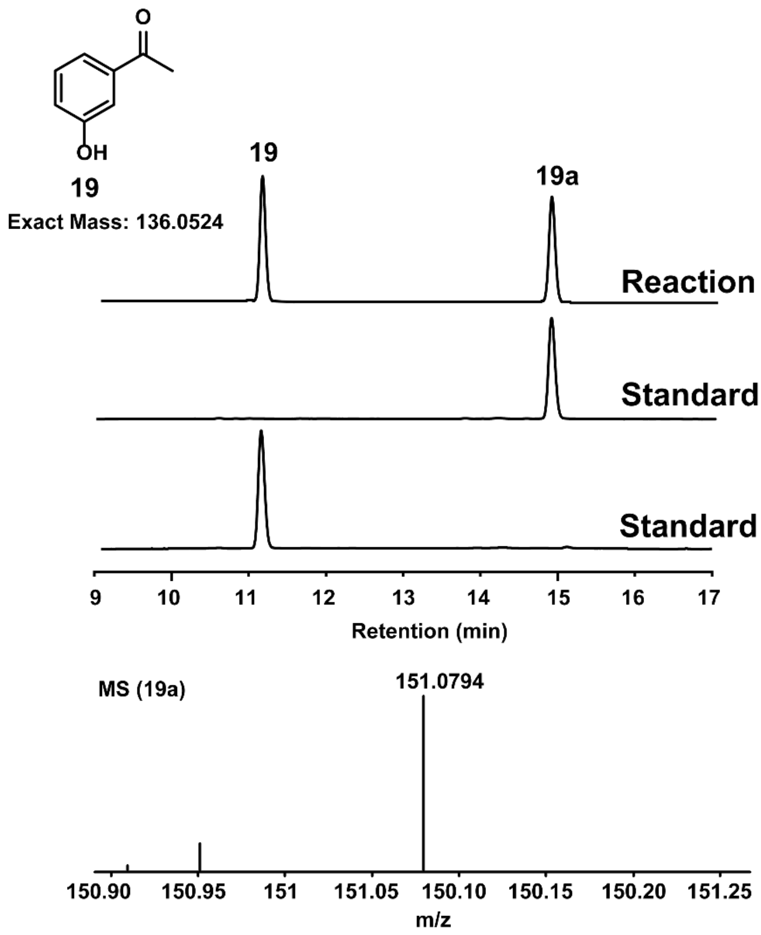

Supplementary Fig 16. HPLC and LC-MS analysis of the products catalyzed by SmOMT using **19** as the substrate.

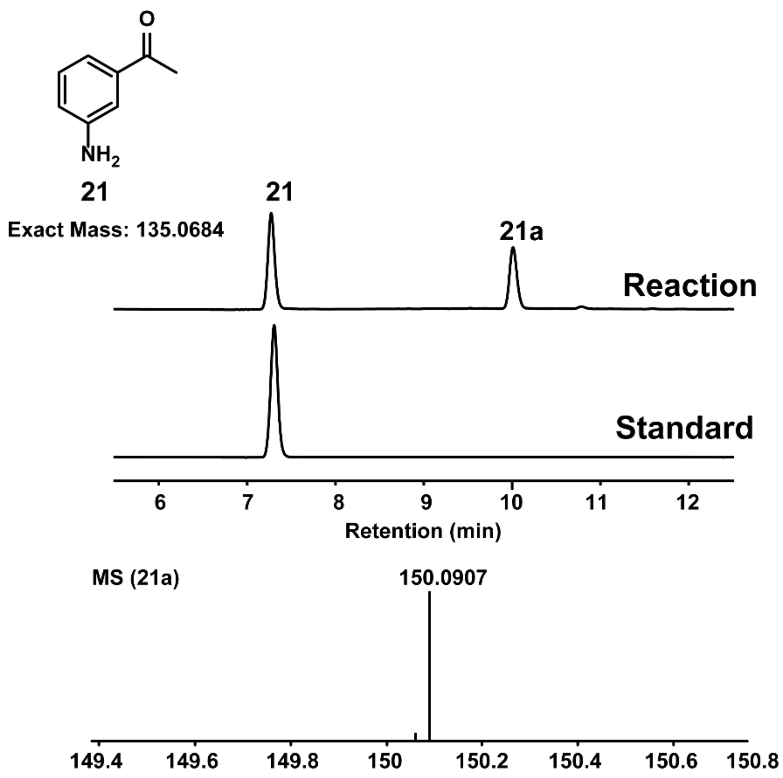

Supplementary Fig 17. HPLC and LC-MS analysis of the products catalyzed by SmOMT using **21** as the substrate.

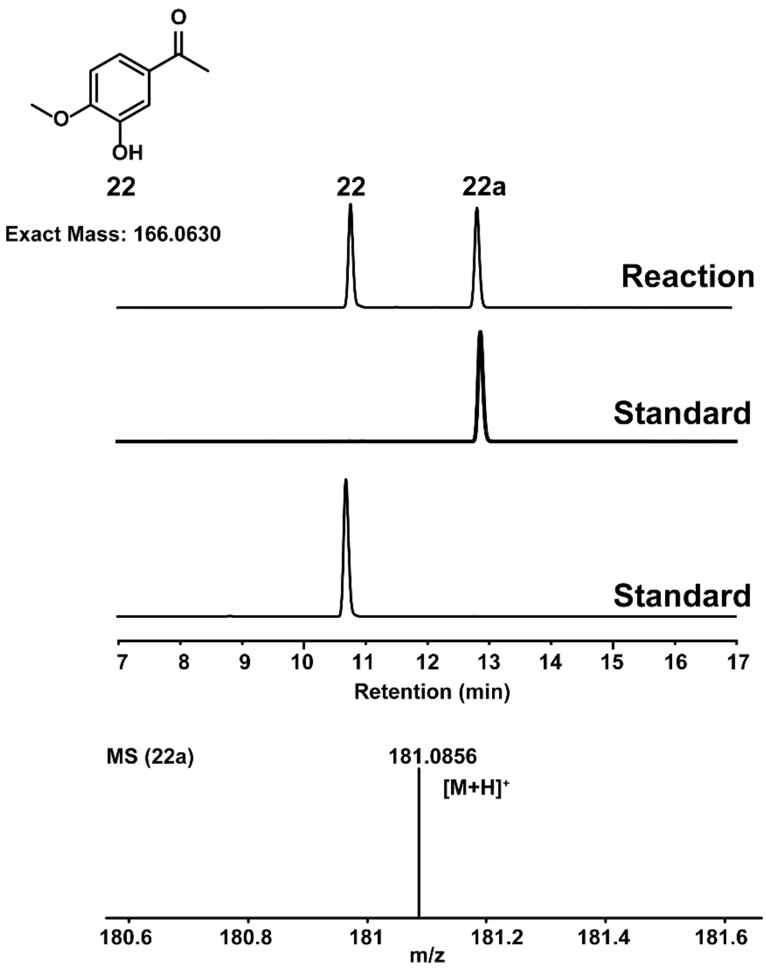

Supplementary Fig 18. HPLC and LC-MS analysis of the products catalyzed by SmOMT using **22** as the substrate.

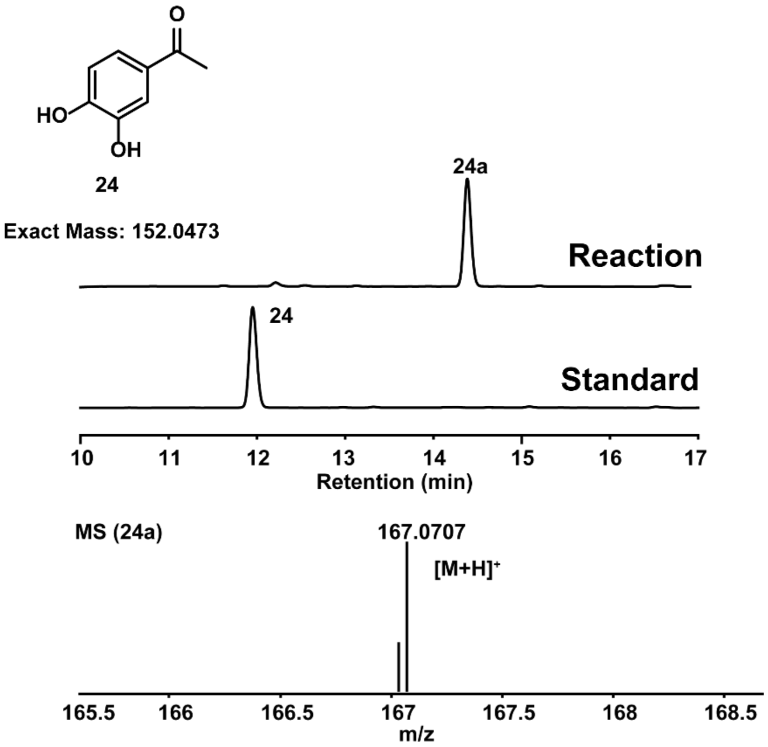

Supplementary Fig 19. HPLC and LC-MS analysis of the products catalyzed by SmOMT using **24** as the substrate.

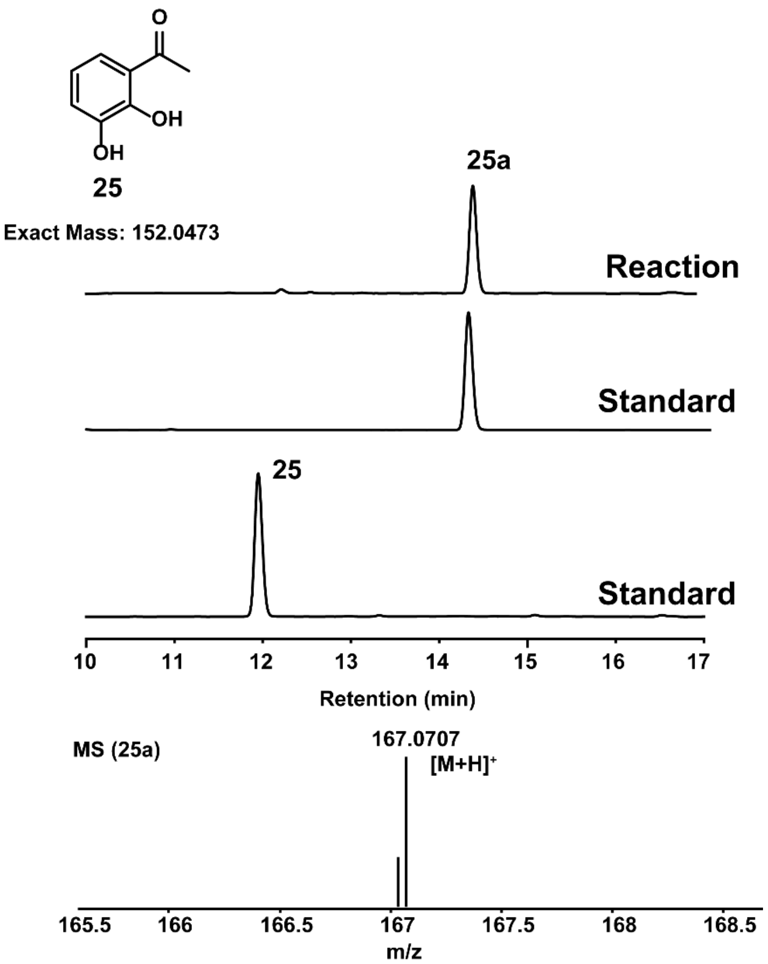

Supplementary Fig 20. HPLC and LC-MS analysis of the products catalyzed by SmOMT using **25** as the substrate.

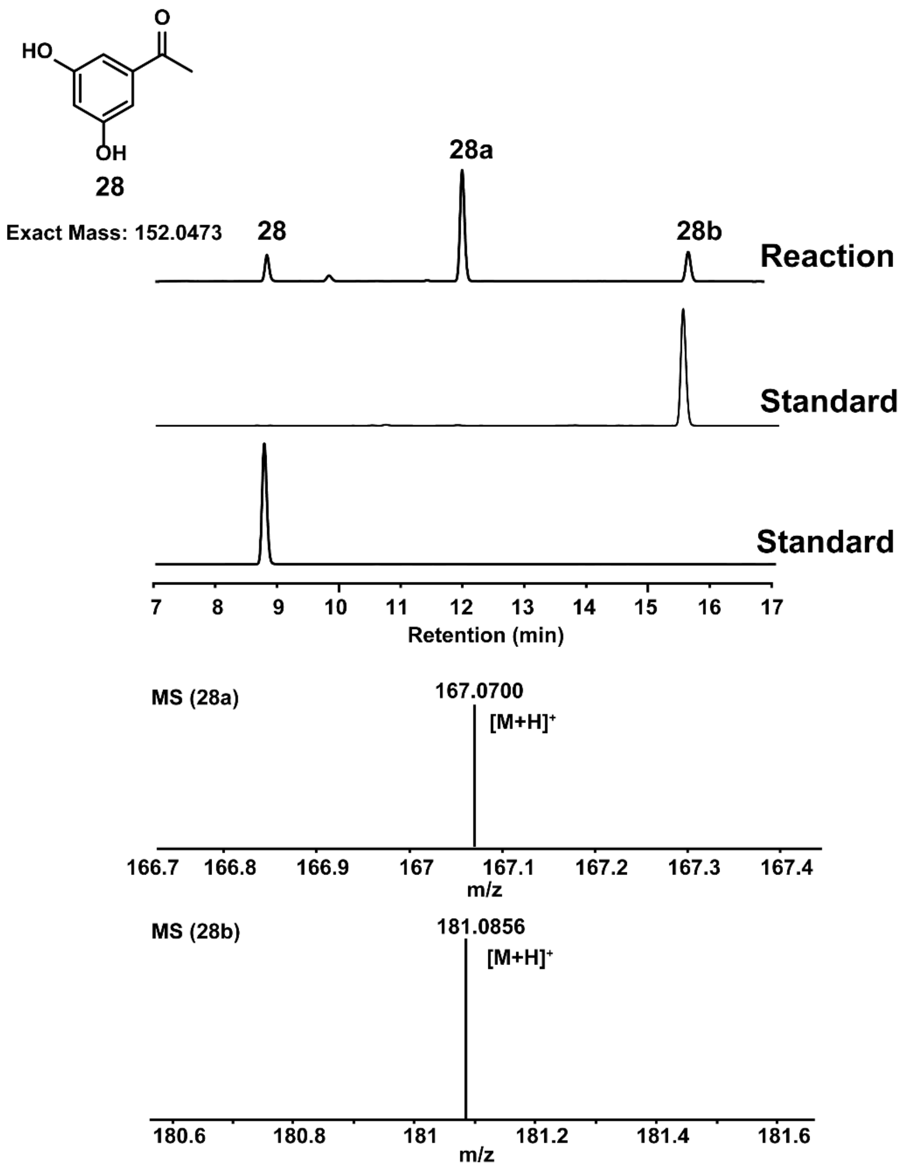

Supplementary Fig 21. HPLC and LC-MS analysis of the products catalyzed by SmOMT using **28** as the substrate.

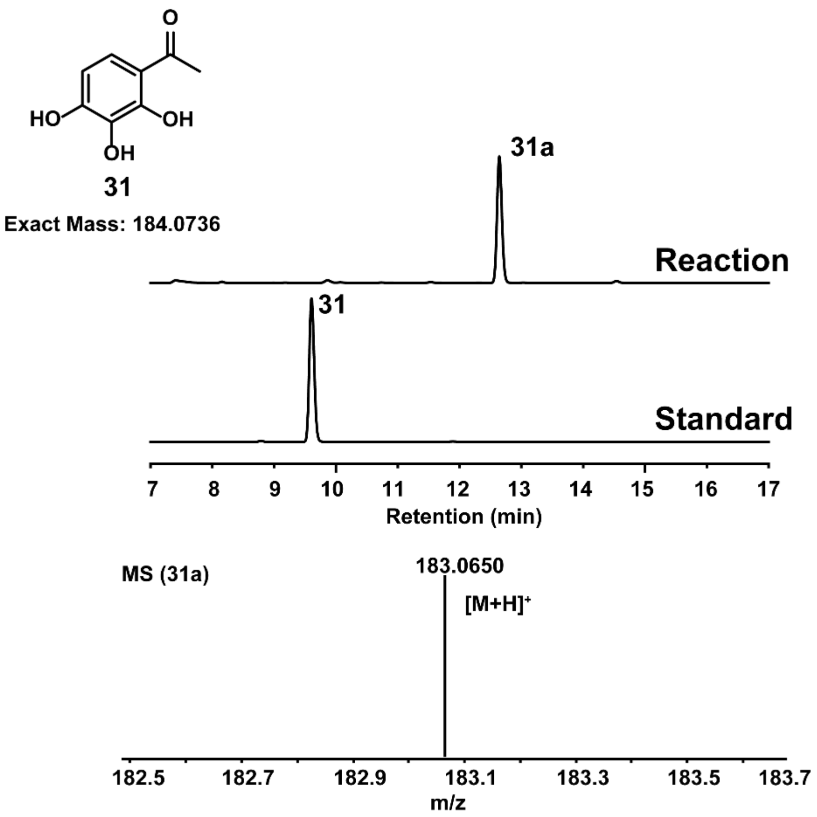

Supplementary Fig 22. HPLC and LC-MS analysis of the products catalyzed by SmOMT using **31** as the substrate.

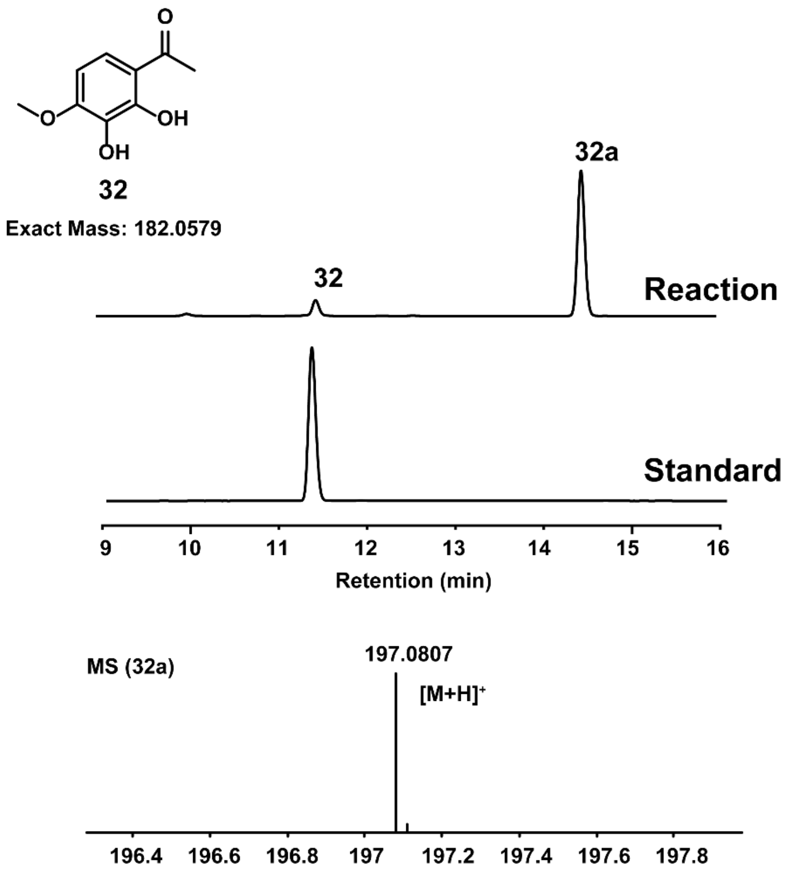

Supplementary Fig 23. HPLC and LC-MS analysis of the products catalyzed by SmOMT using **32** as the substrate.

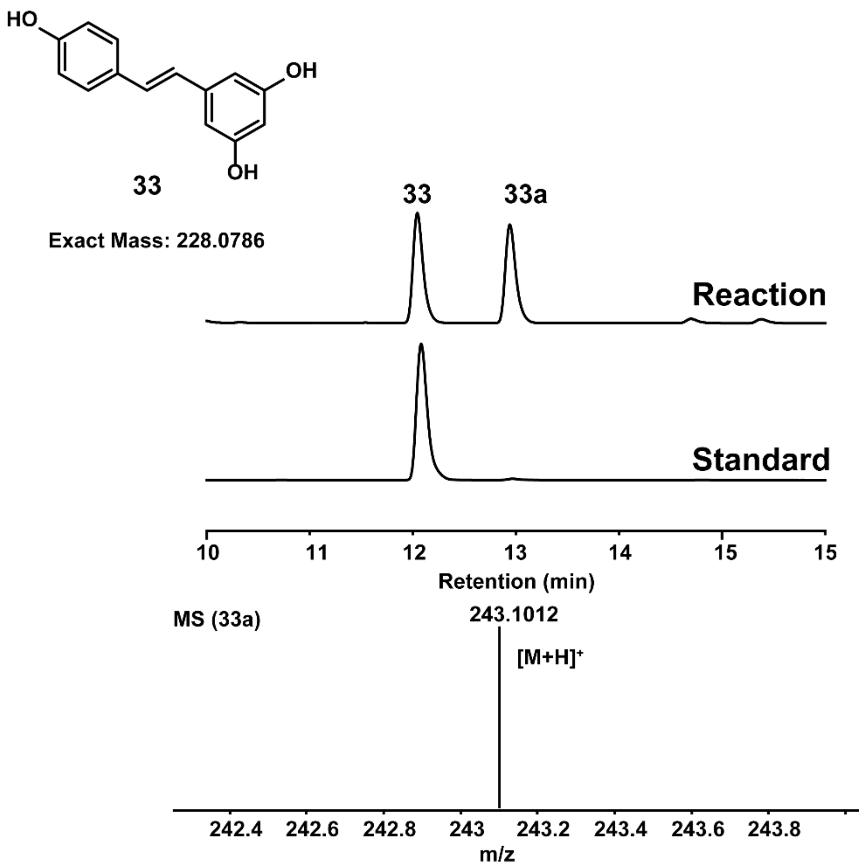

Supplementary Fig 24. HPLC and LC-MS analysis of the products catalyzed by SmOMT using **33** as the substrate.

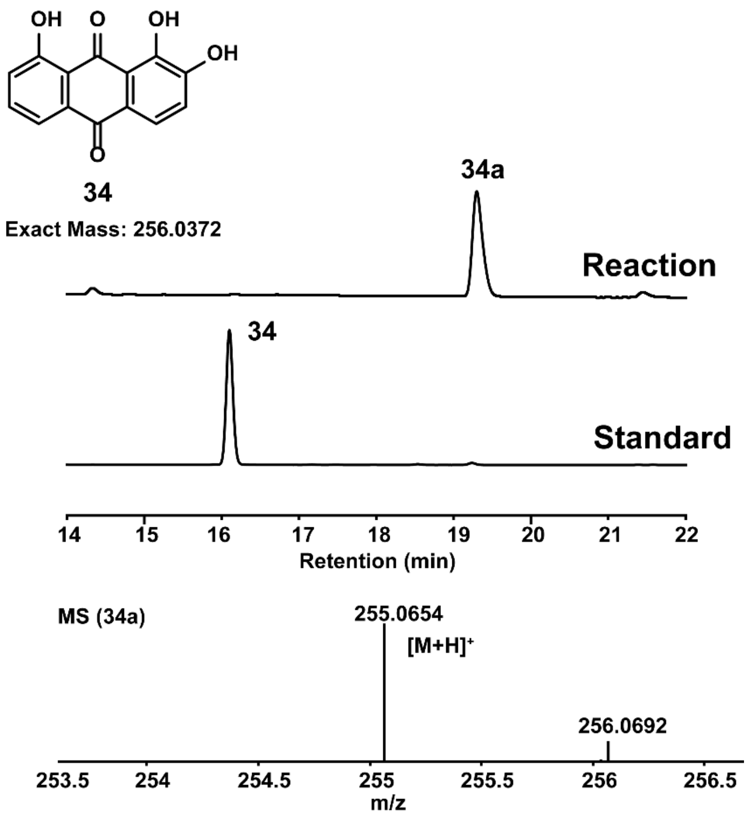

Supplementary Fig 25. HPLC and LC-MS analysis of the products catalyzed by SmOMT using **34** as the substrate.

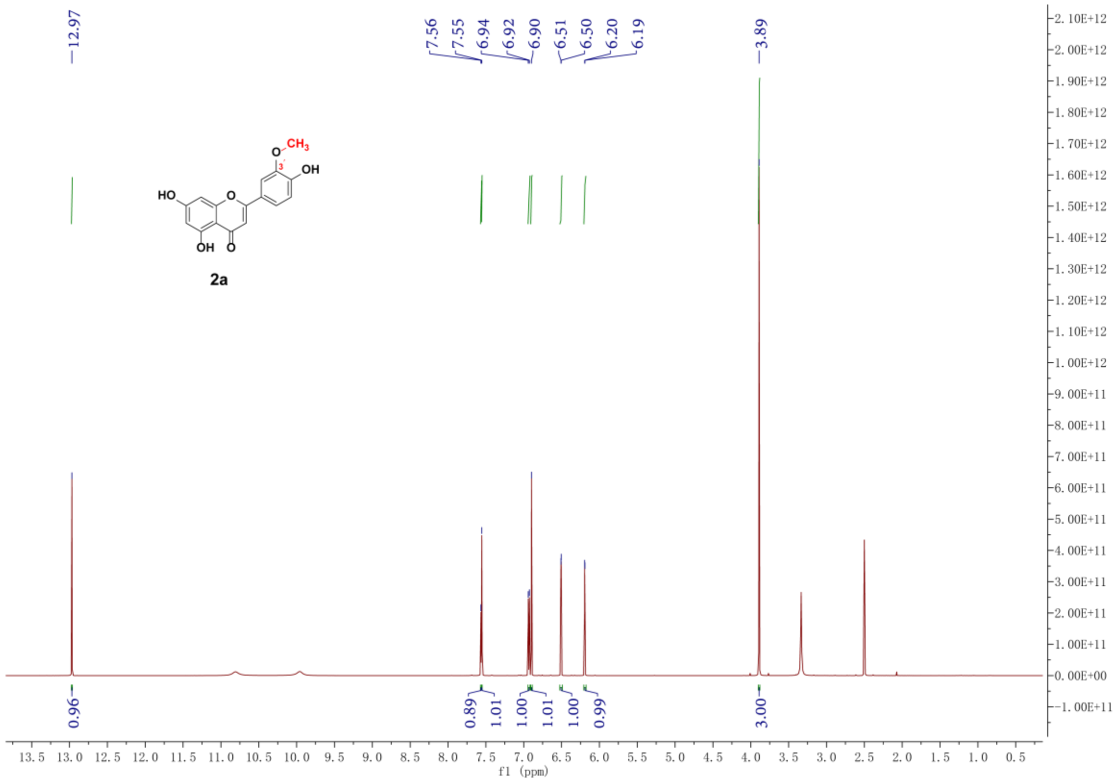

Supplementary Fig 26. ^{1}H NMR spectrum of **2a** in DMSO-d_6 (600 MHz)

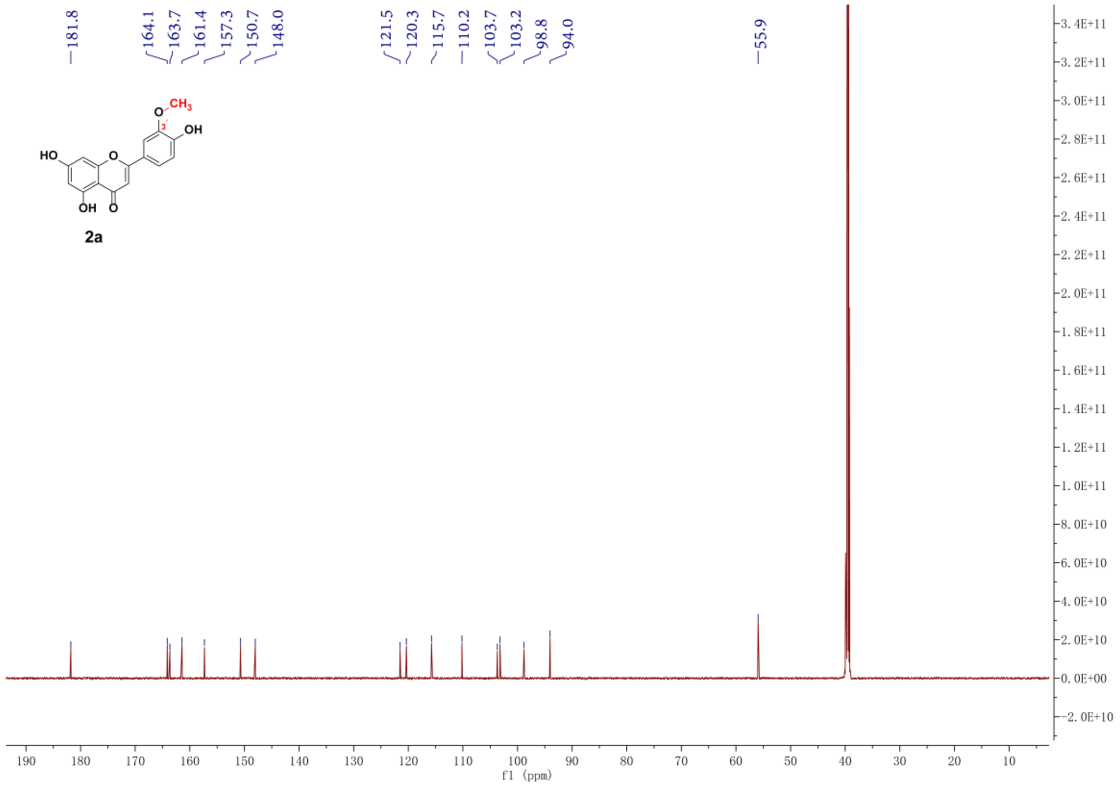

Supplementary Fig 27. ^{13}C NMR spectrum of **2a** in DMSO-d_6 (150 MH)

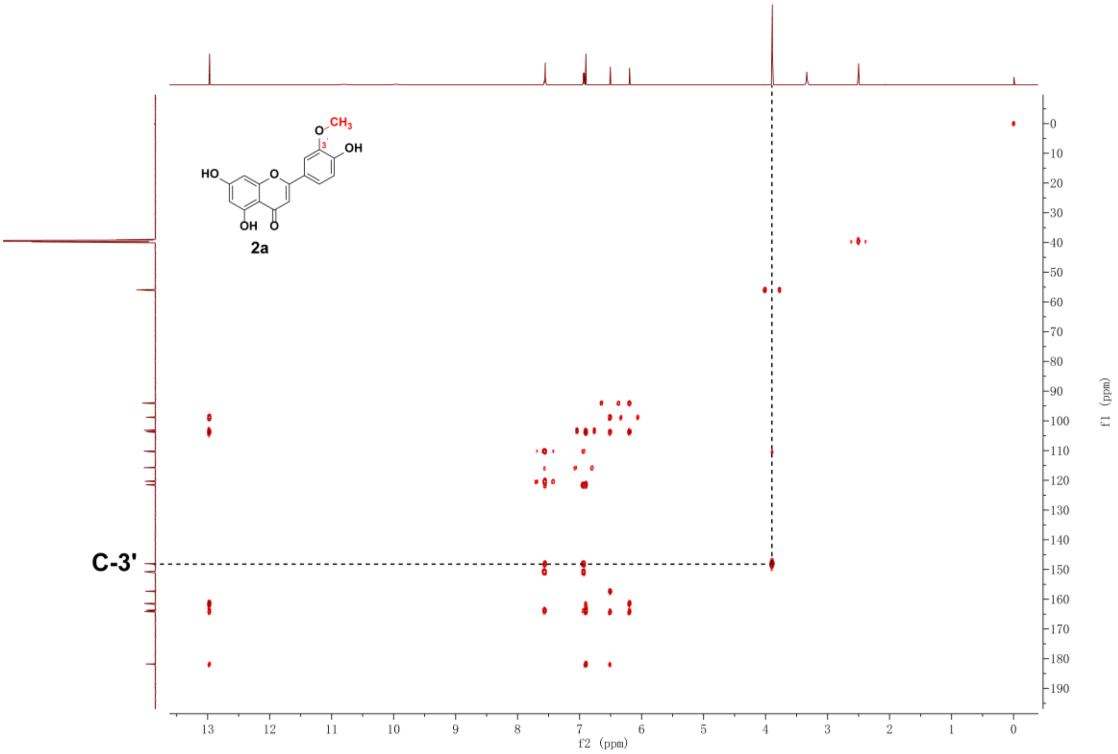

Supplementary Fig 28. HMBC spectrum of **2a** in DMSO-d_6 (150 MHz)

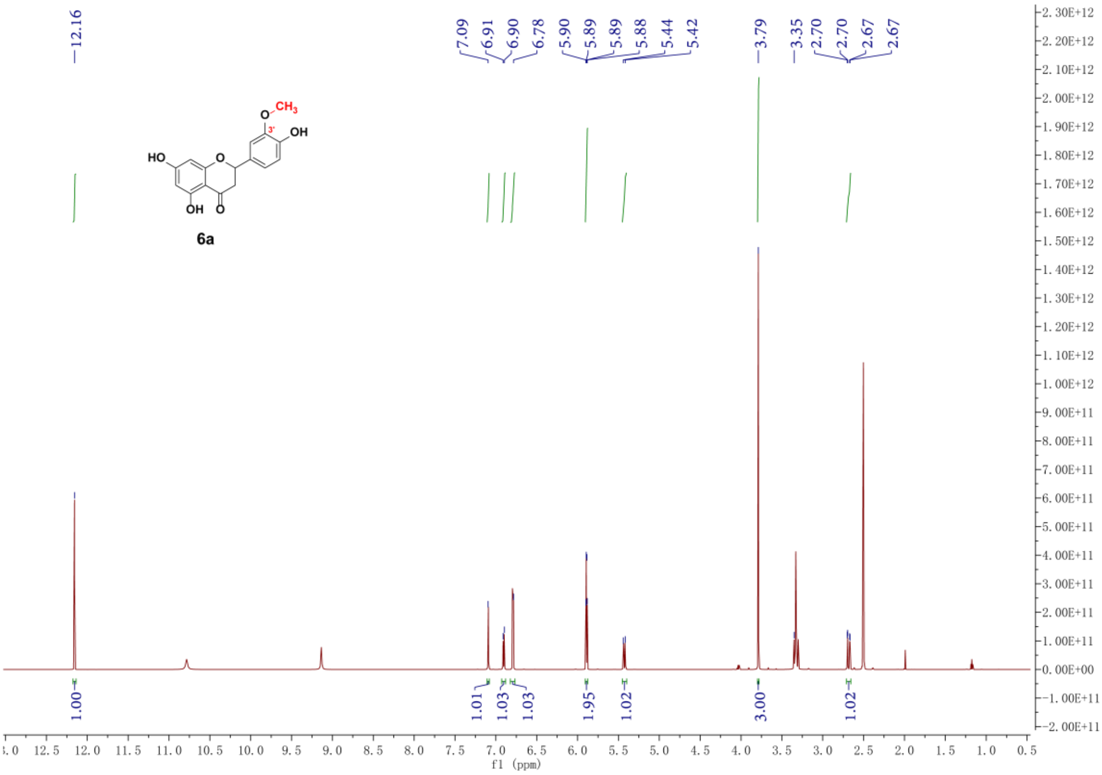

Supplementary Fig 29. ^{1}H NMR spectrum of **8a** in DMSO-d_6 (600 MHz)

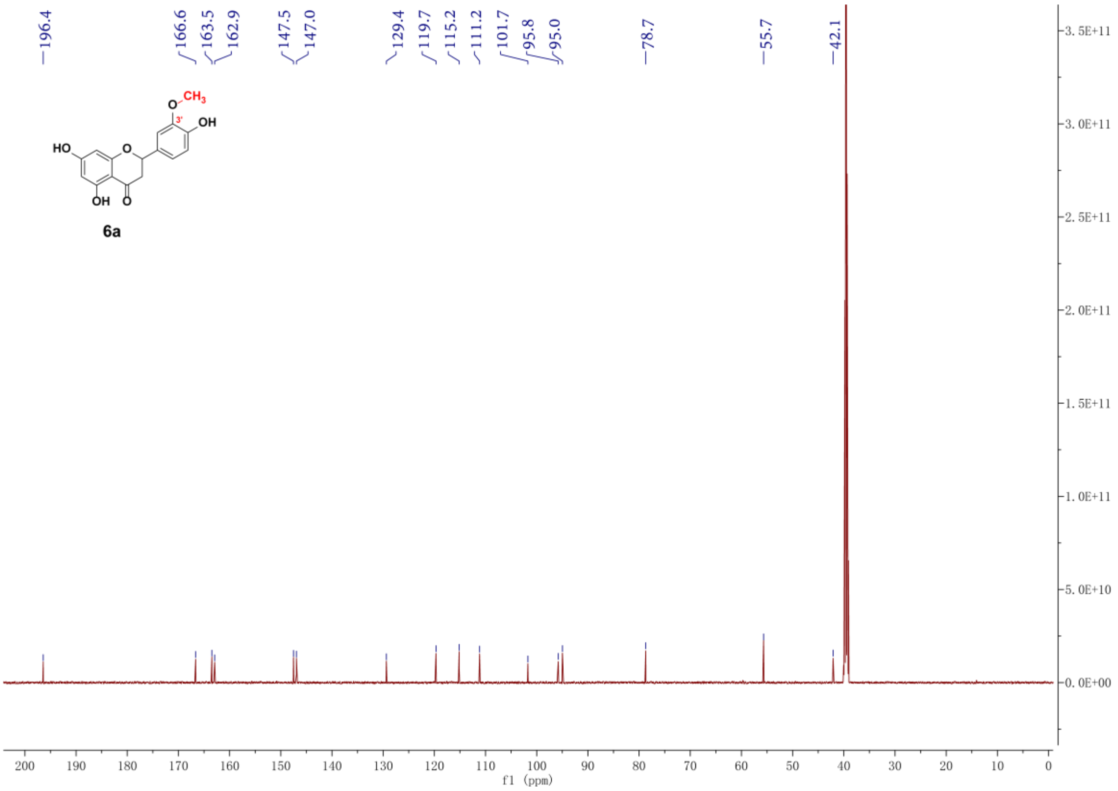

Supplementary Fig 30. ^{13}C NMR spectrum of **6a** in DMSO-d_6 (150 MH)

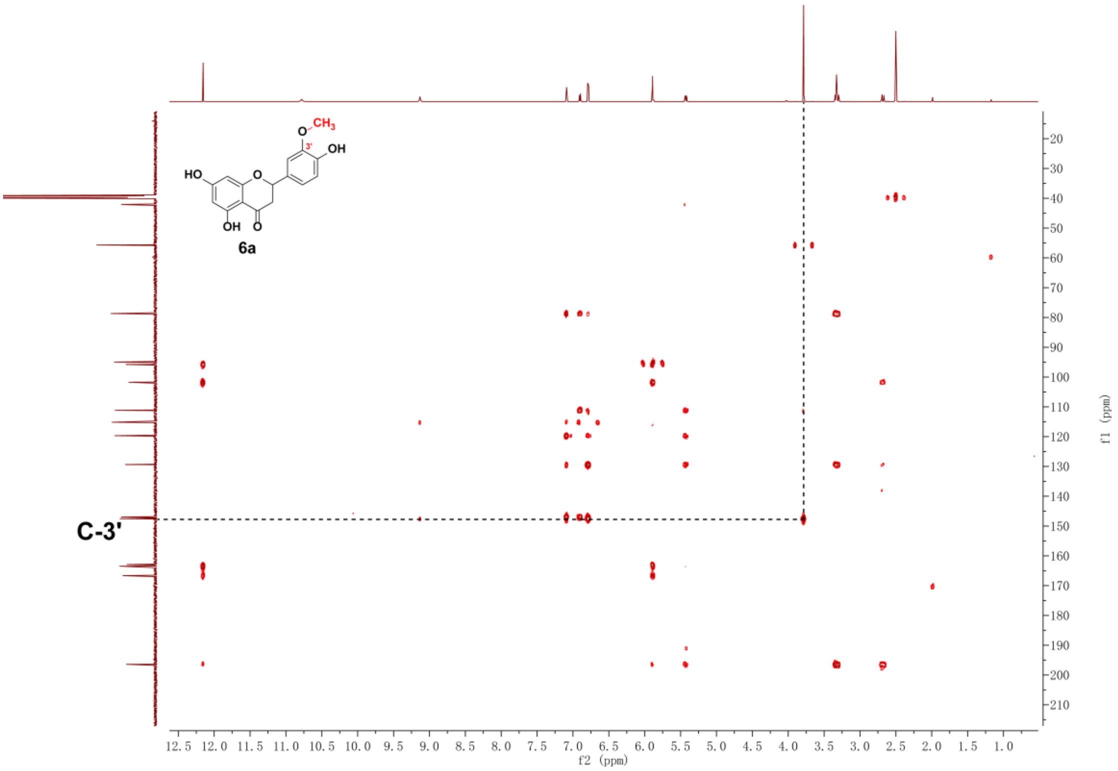

Supplementary Fig 31. HMBC spectrum of **6a** in DMSO-d_6 (150 MHz)

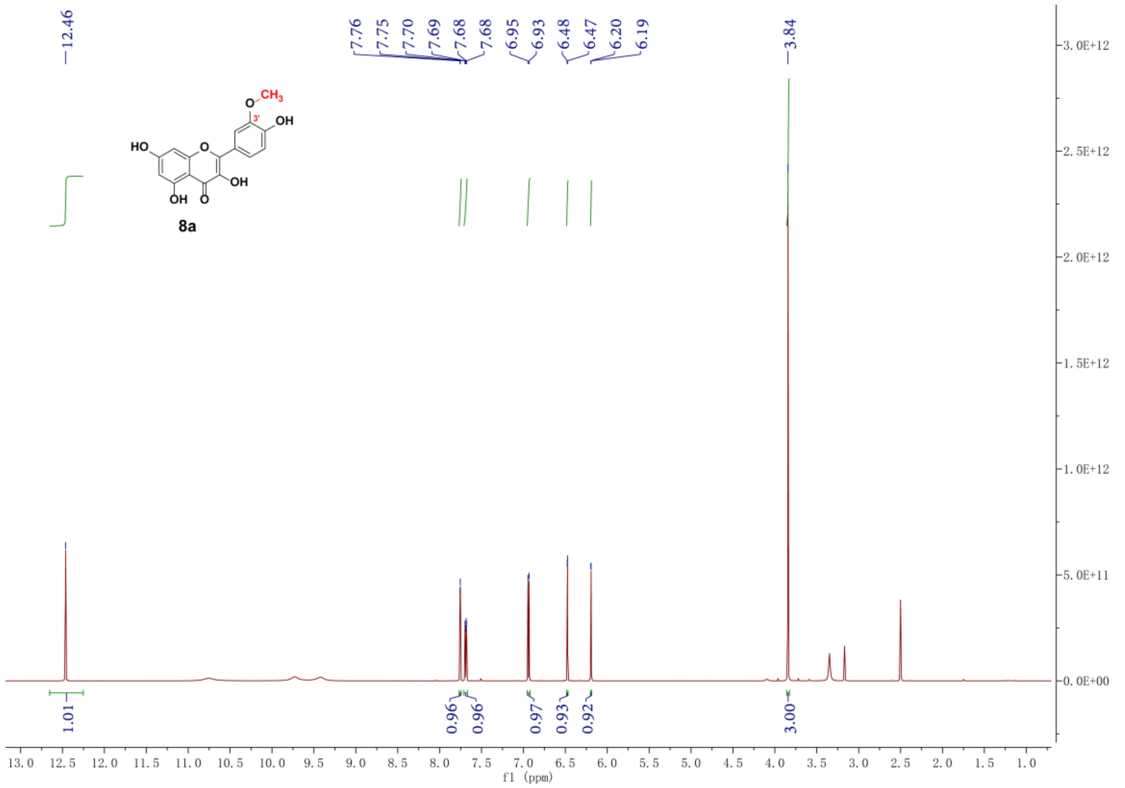

Supplementary Fig 32. ^{1}H NMR spectrum of **6a** in DMSO-d_6 (600 MHz)

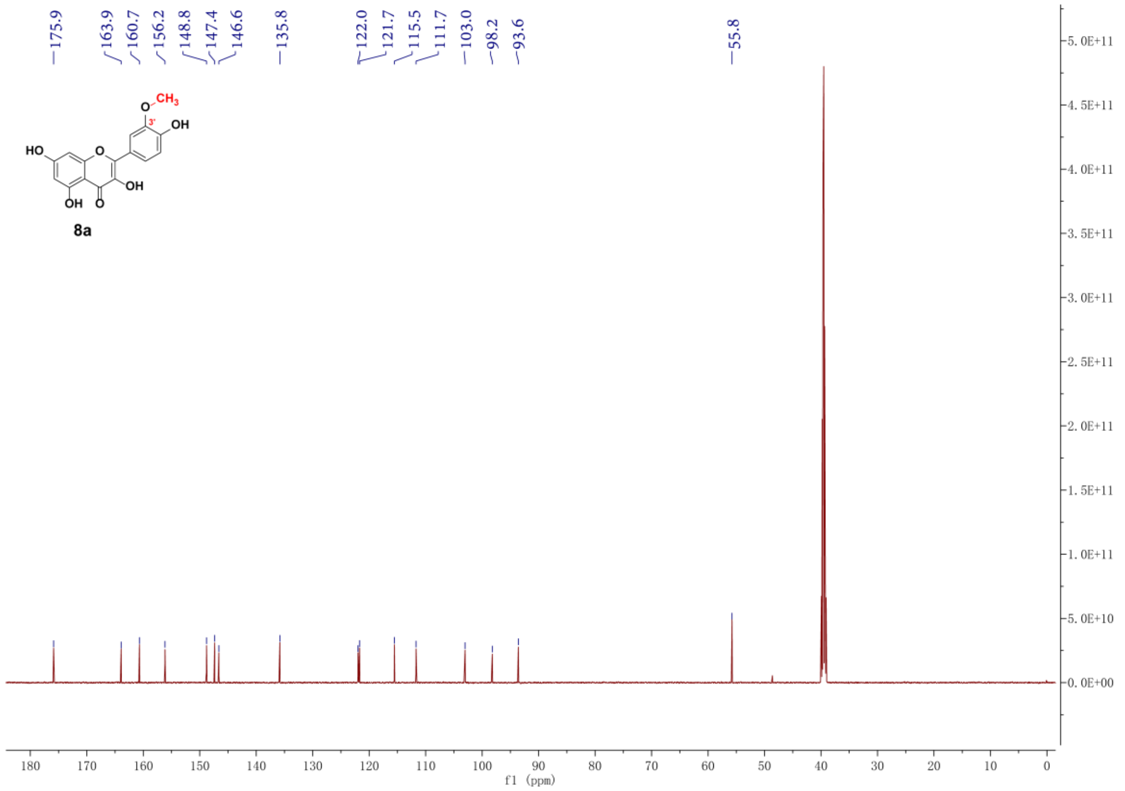

Supplementary Fig 33. ^{13}C NMR spectrum of **8a** in DMSO-d_6 (150 MH)

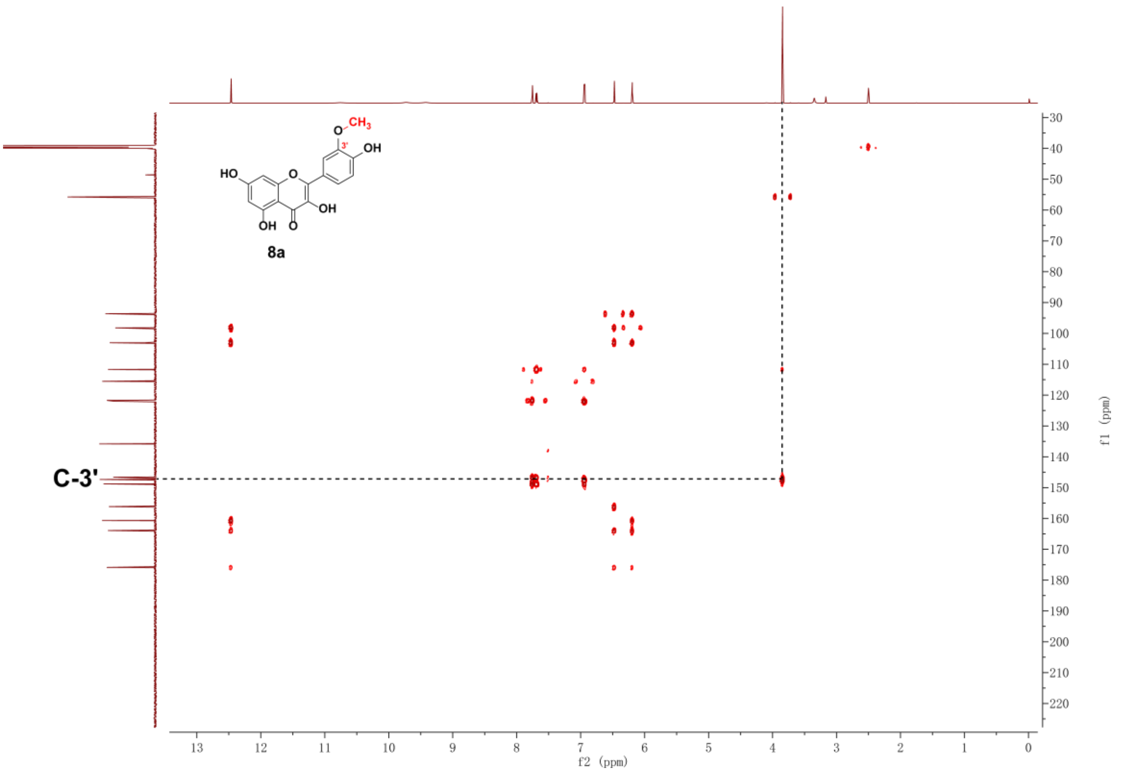

Supplementary Fig 34. HMBC spectrum of **8a** in DMSO-d_6 (150 MHz)

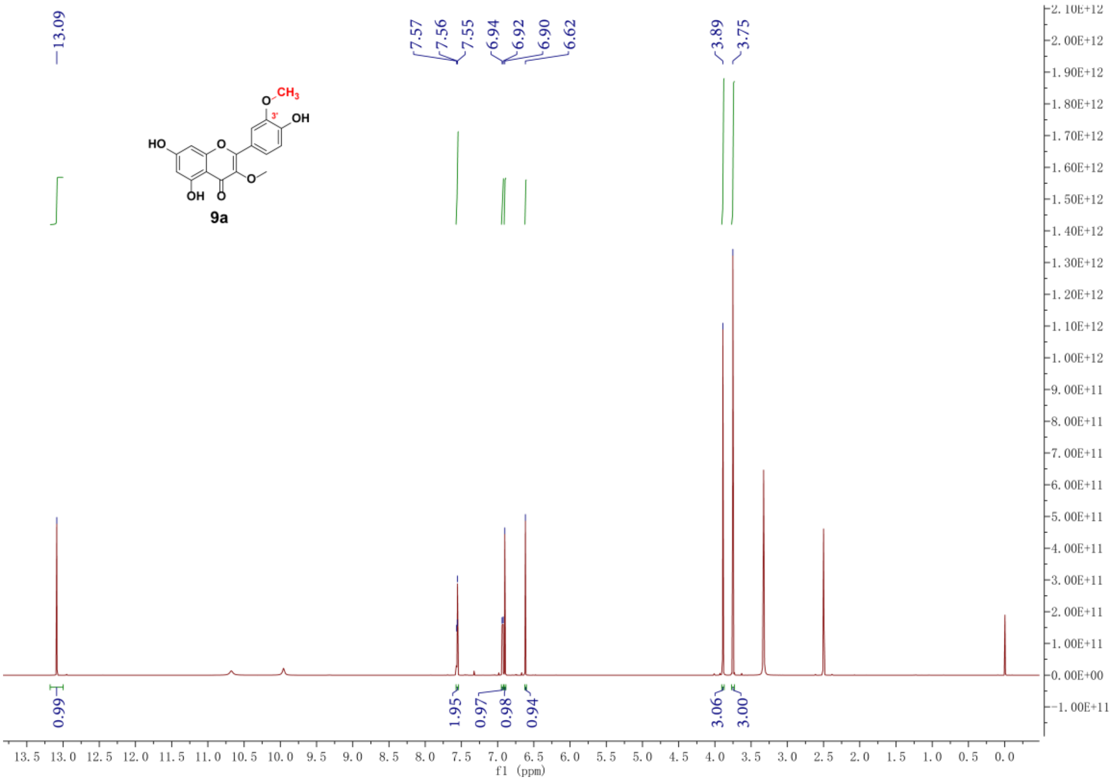

Supplementary Fig 35. ^{1}H NMR spectrum of **9a** in DMSO-d_6 (600 MHz)

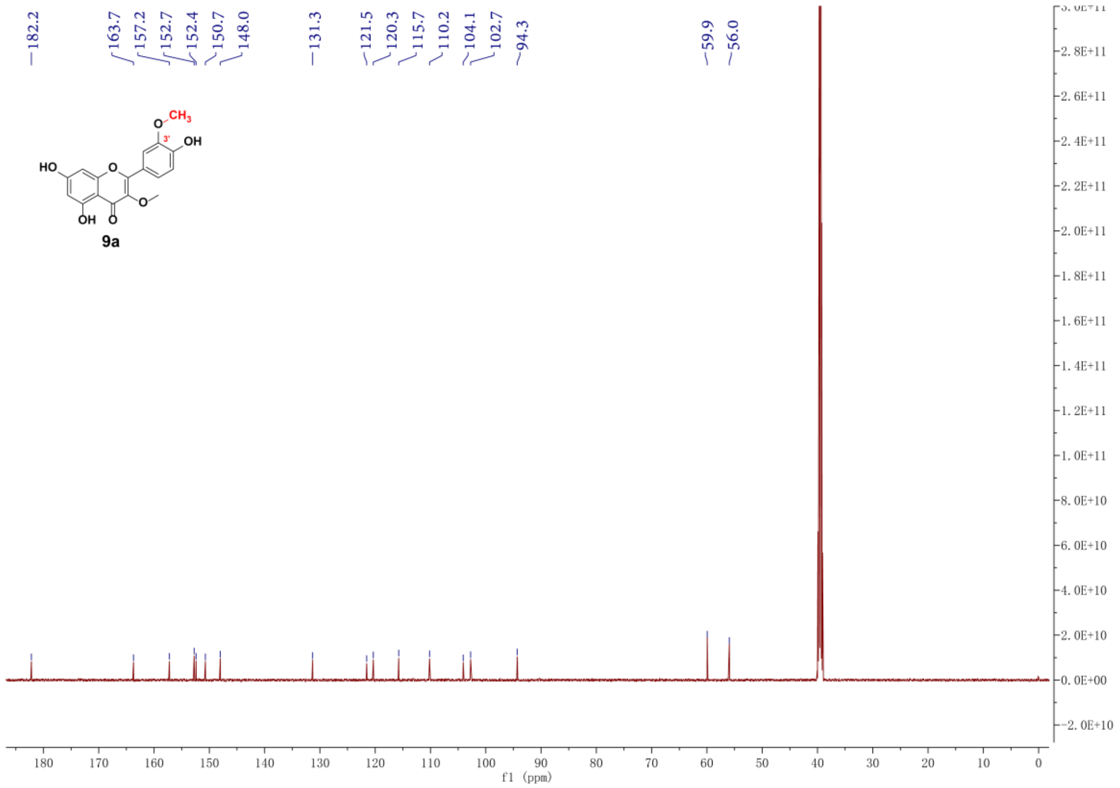

Supplementary Fig 36. ^{13}C NMR spectrum of **9a** in DMSO-d_6 (150 MH)

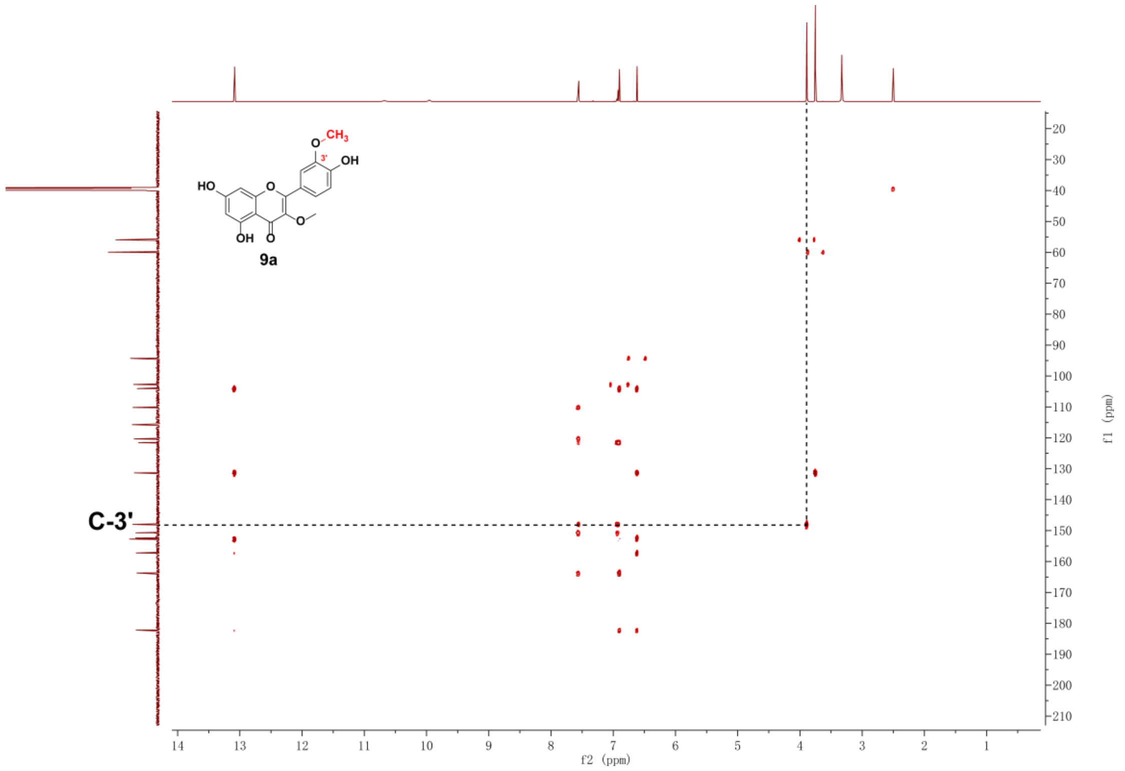

Supplementary Fig 37. HMBC spectrum of **9a** in DMSO-d_6 (150 MHz)

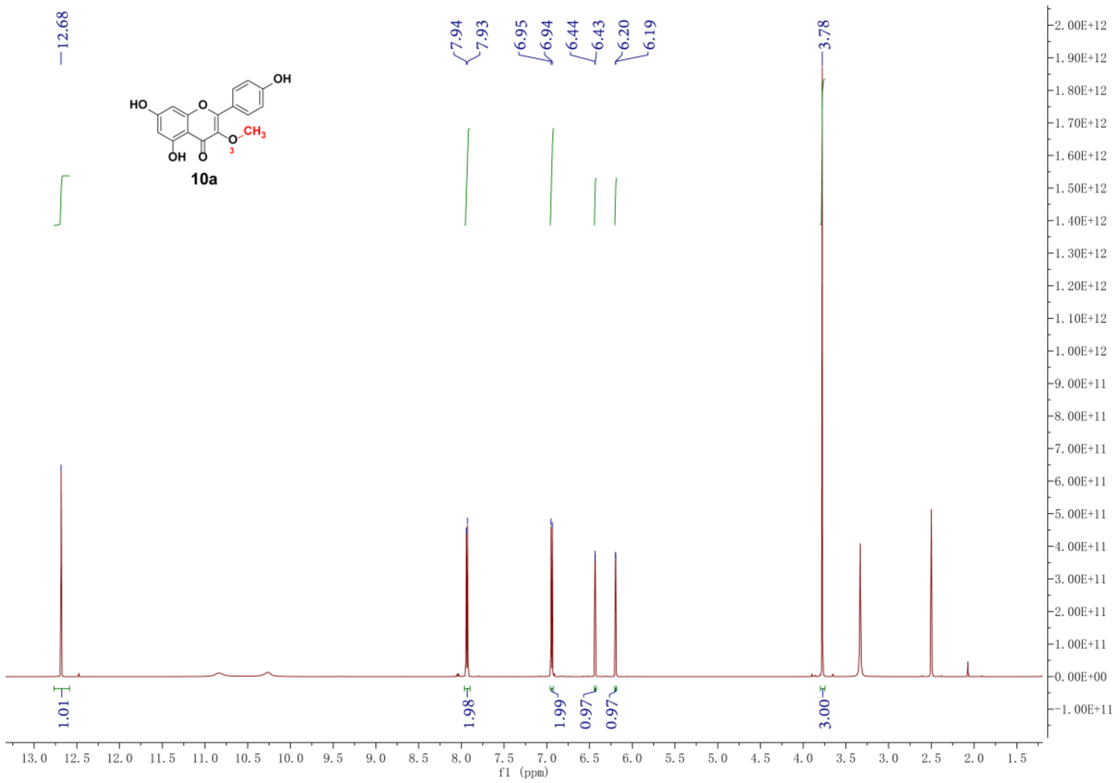

Supplementary Fig 38. ^{1}H NMR spectrum of **10a** in DMSO-d_6 (600 MHz)

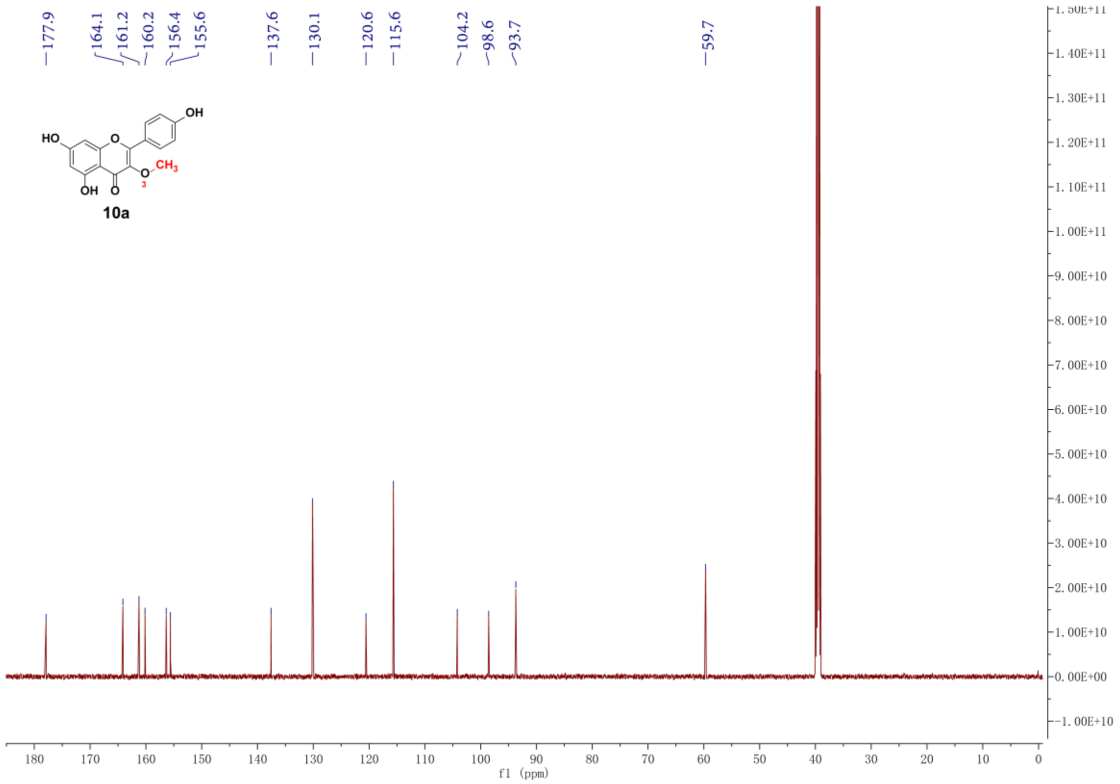

Supplementary Fig 39. ^{13}C NMR spectrum of **10a** in DMSO-d_6 (150 MH)

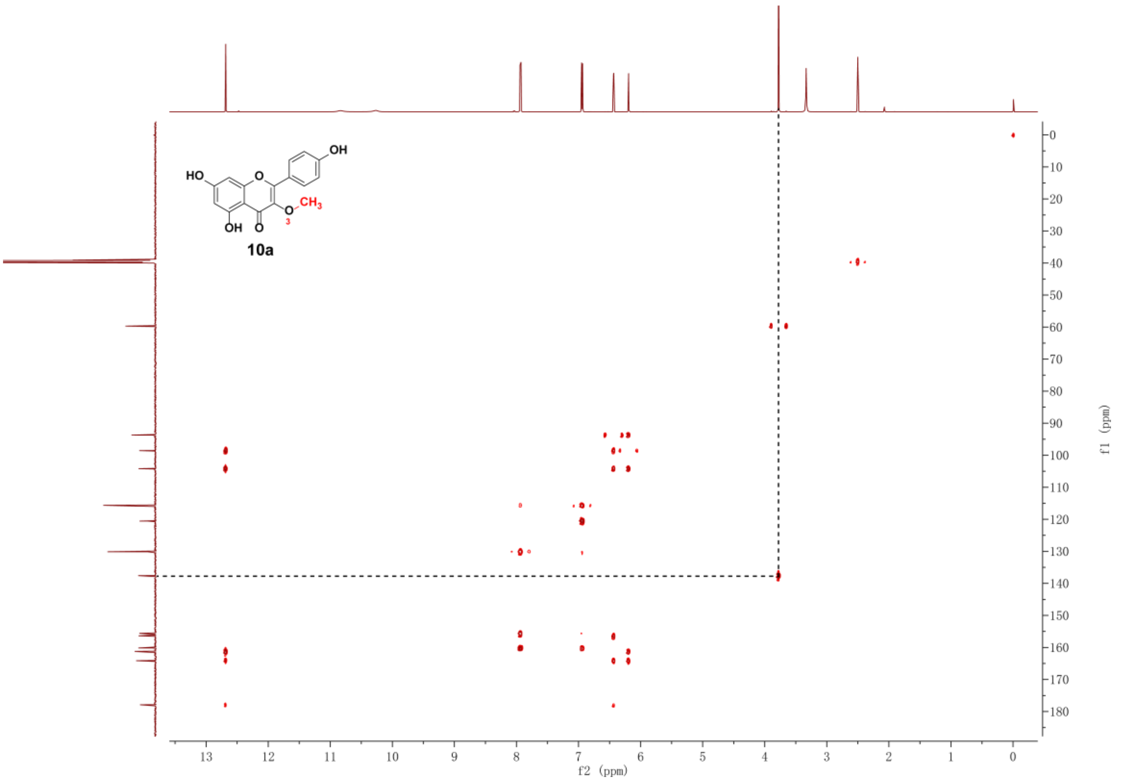

Supplementary Fig 40. HMBC spectrum of **10a** in DMSO-d_6 (150 MHz)

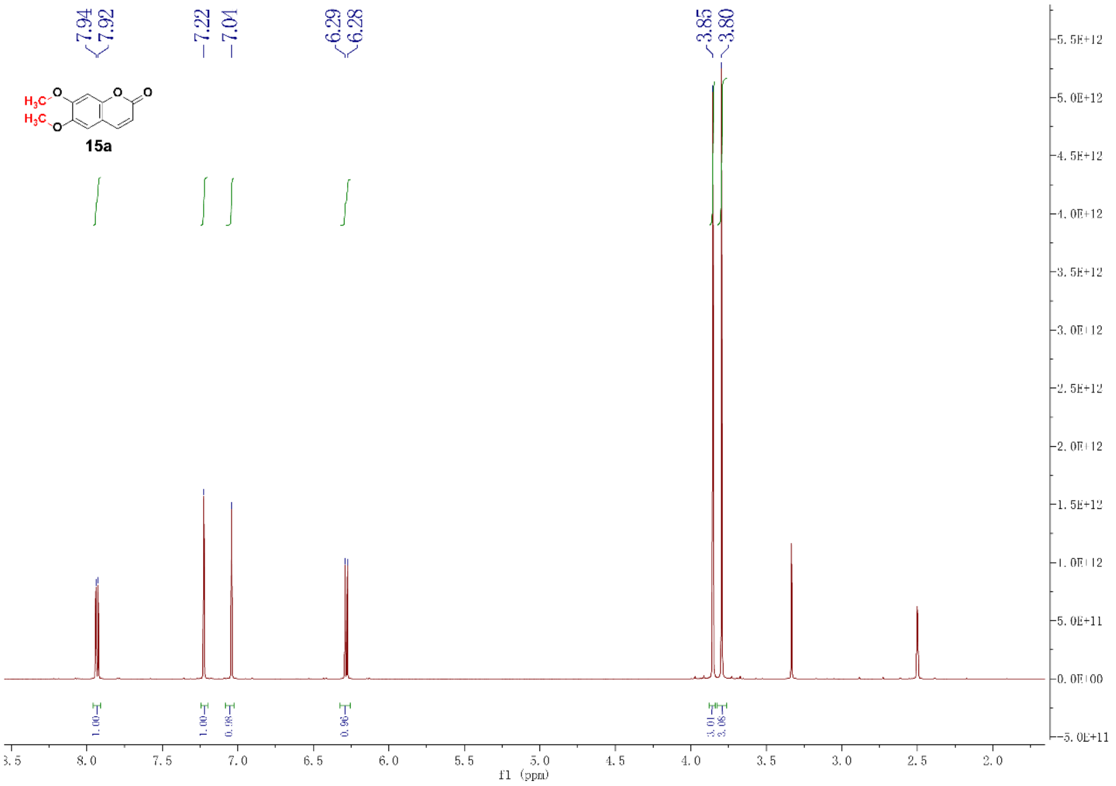

Supplementary Fig 41. ^{1}H NMR spectrum of **15a** in DMSO-d_6 (600 MHz)

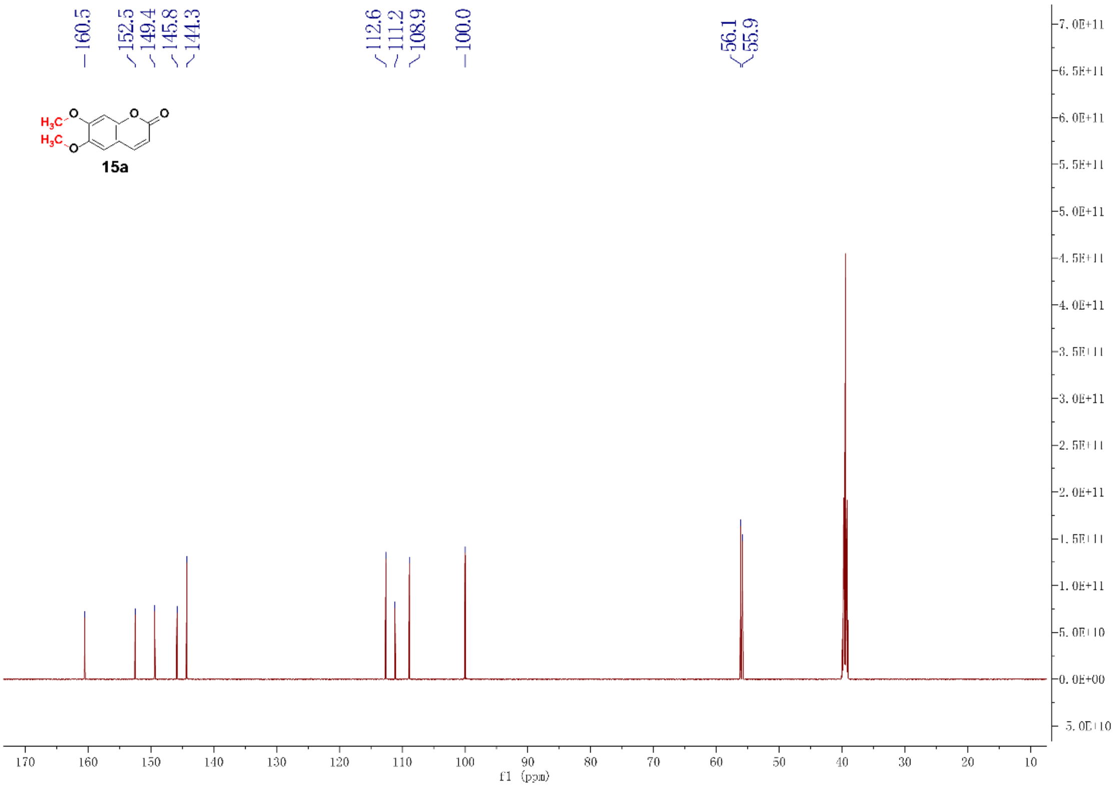

Supplementary Fig 42. ^{13}C NMR spectrum of **15a** in DMSO-d_6 (150 MH)

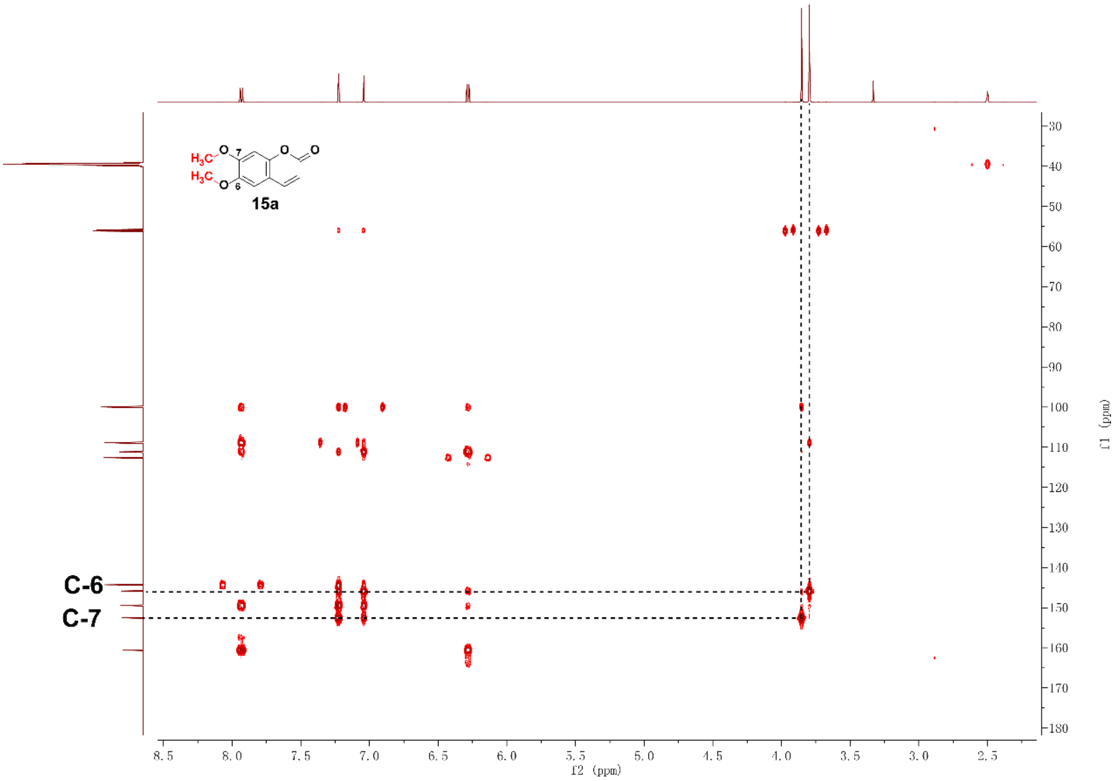

Supplementary Fig 43. HMBC spectrum of **15a** in DMSO-d_6 (150 MHz)

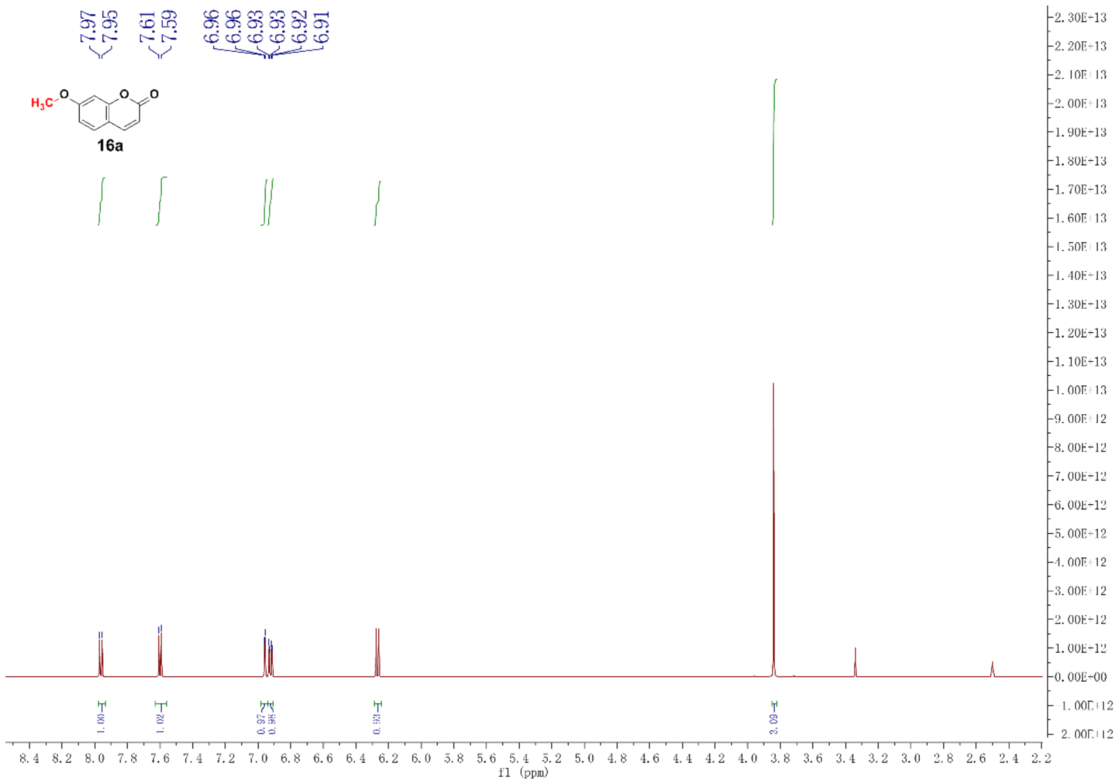

Supplementary Fig 44. ^{1}H NMR spectrum of **16a** in DMSO-d_6 (600 MHz)

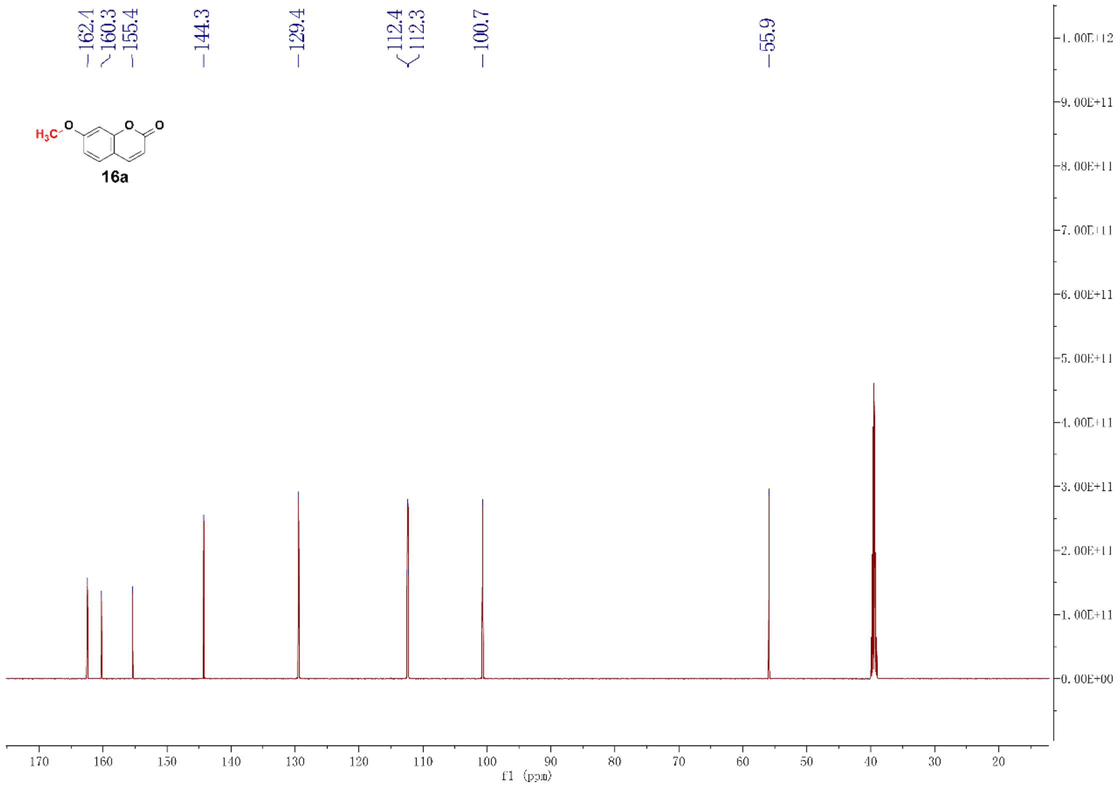

Supplementary Fig 45. ^{13}C NMR spectrum of **16a** in DMSO-d_6 (150 MH)

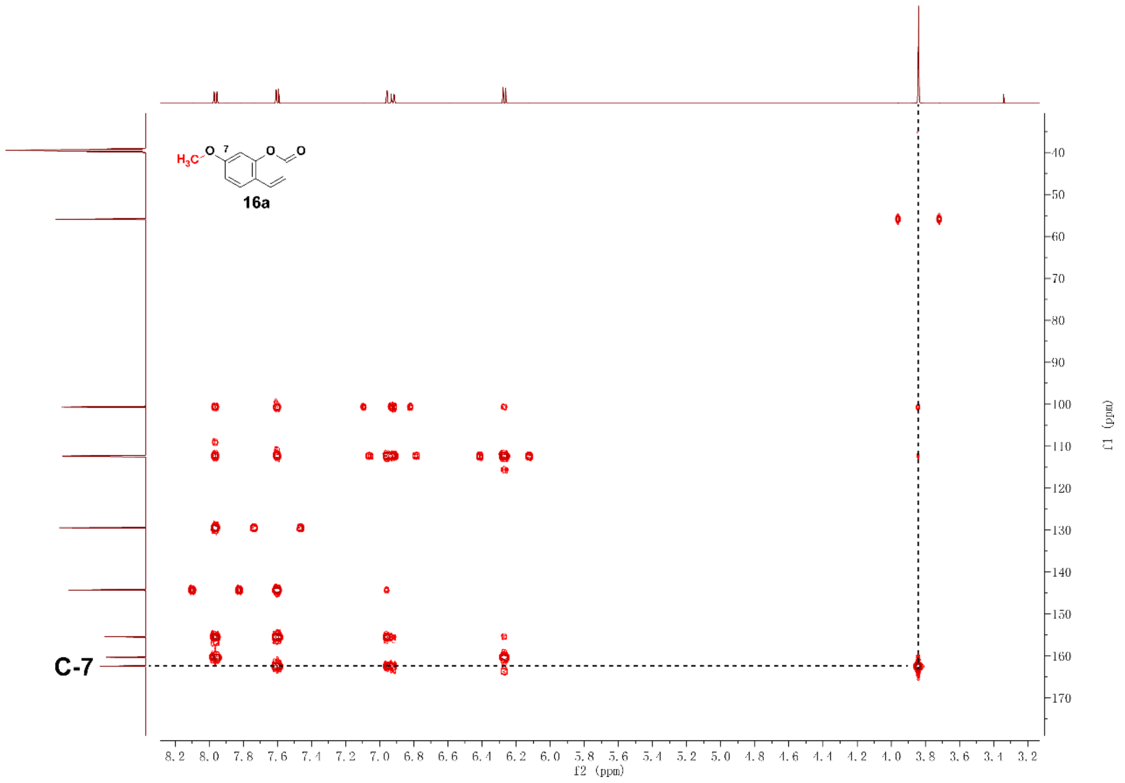

Supplementary Fig 46. HMBC spectrum of **16a** in DMSO-d_6 (150 MHz)

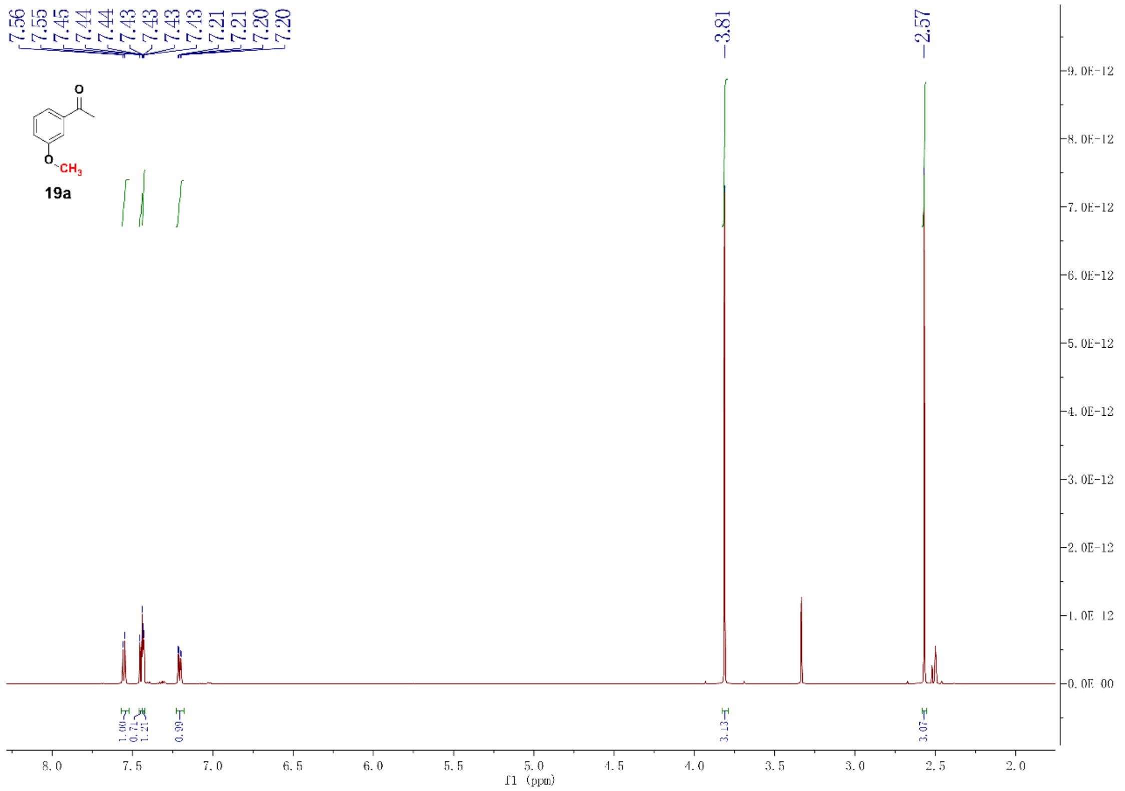

Supplementary Fig 47. ^{1}H NMR spectrum of **19a** in DMSO-d_6 (600 MHz)

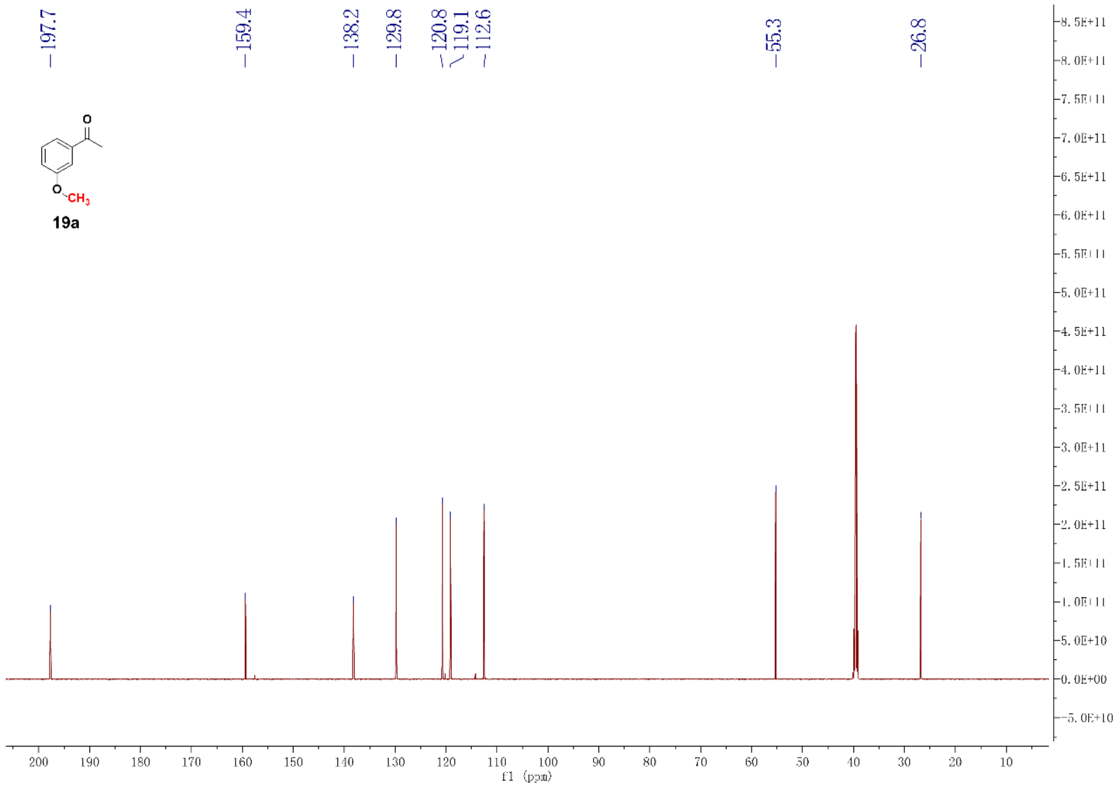

Supplementary Fig 48. ^{13}C NMR spectrum of **19a** in DMSO-d_6 (150 MH)

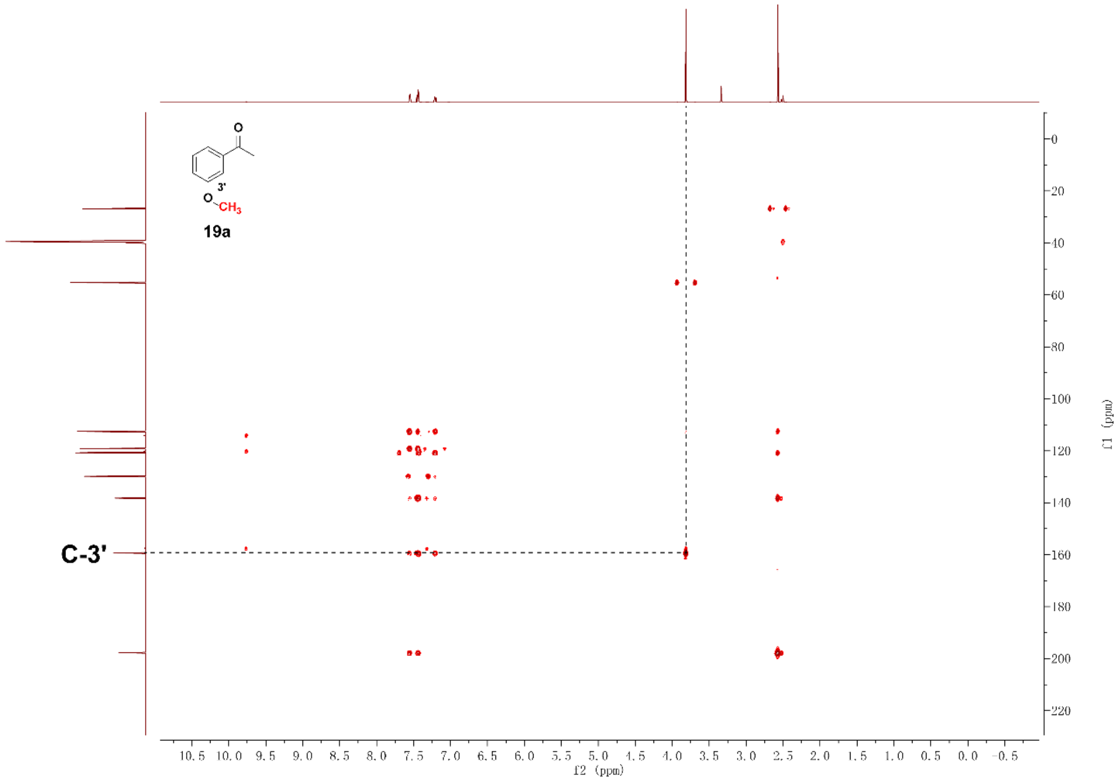

Supplementary Fig 49. HMBC spectrum of **19a** in DMSO-d_6 (150 MHz)

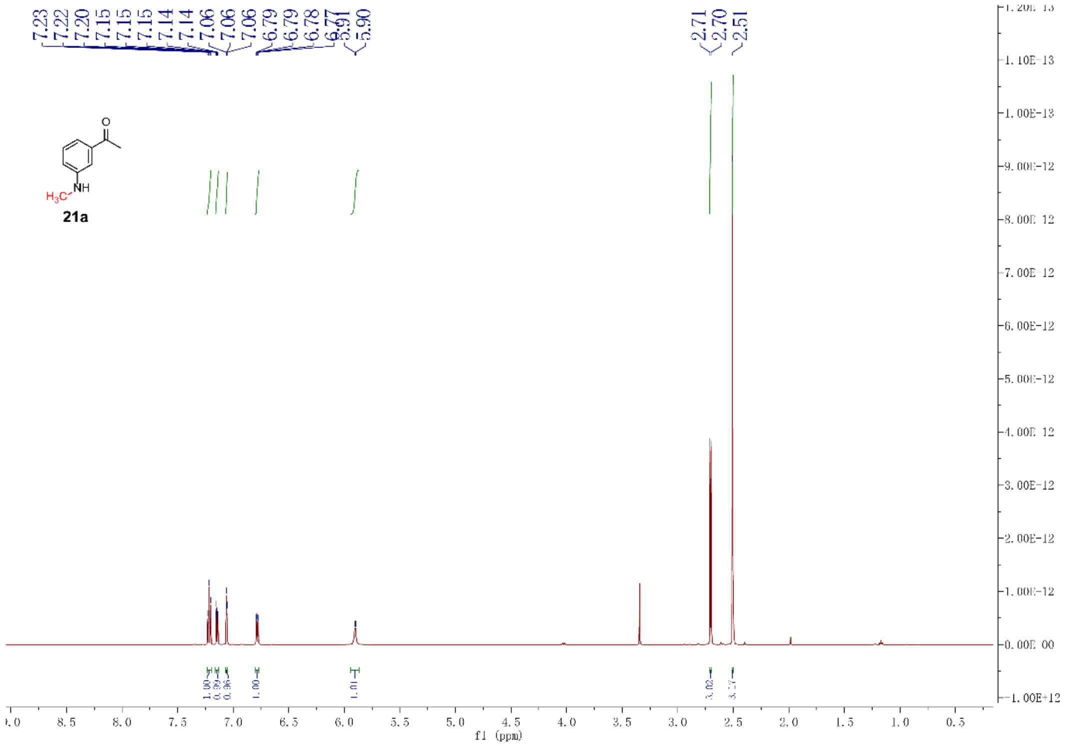

Supplementary Fig 50. ^{1}H NMR spectrum of **21a** in DMSO-d_6 (600 MHz)

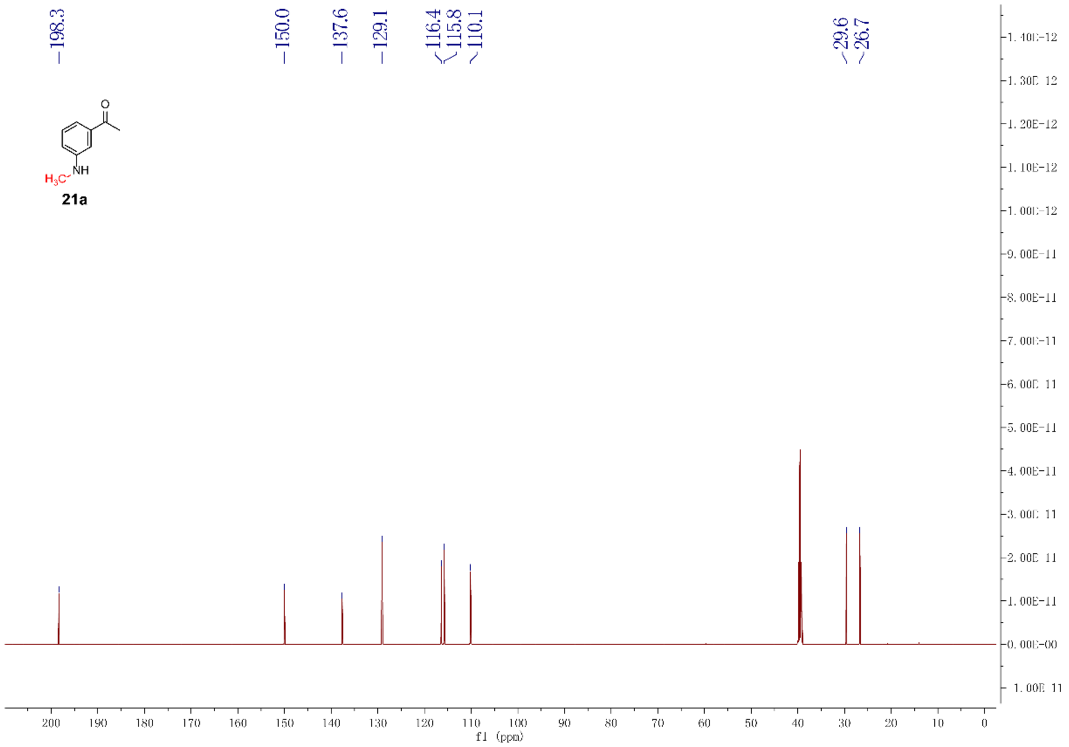

Supplementary Fig 51. ^{13}C NMR spectrum of **21a** in DMSO-d_6 (150 MH)

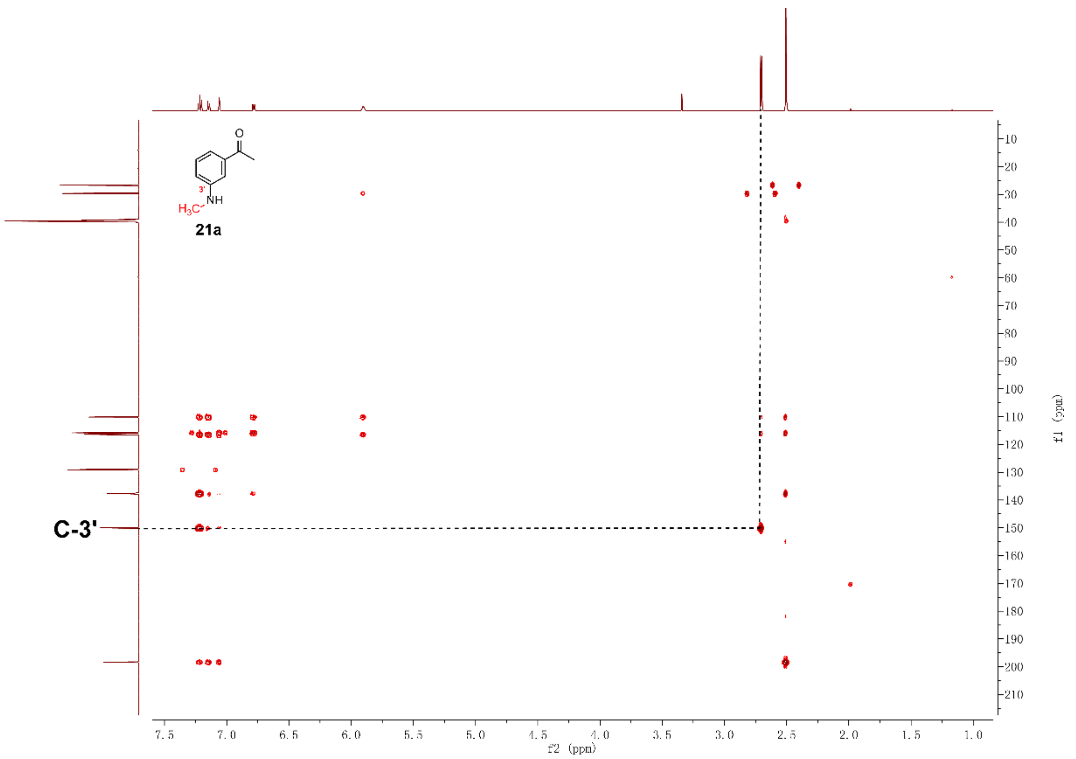

Supplementary Fig52. HMBC spectrum of **21a** in DMSO-d_6 (150 MHz)

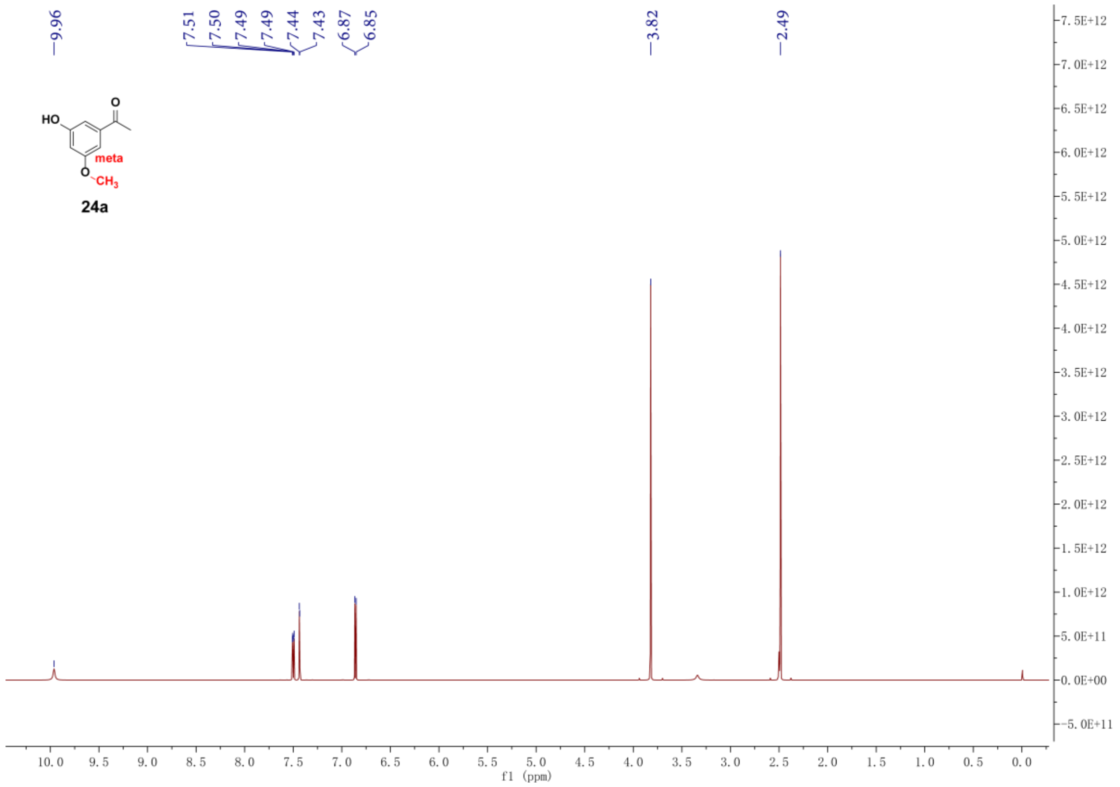

Supplementary Fig 53. ^{1}H NMR spectrum of **24a** in DMSO-d_6 (600 MHz)

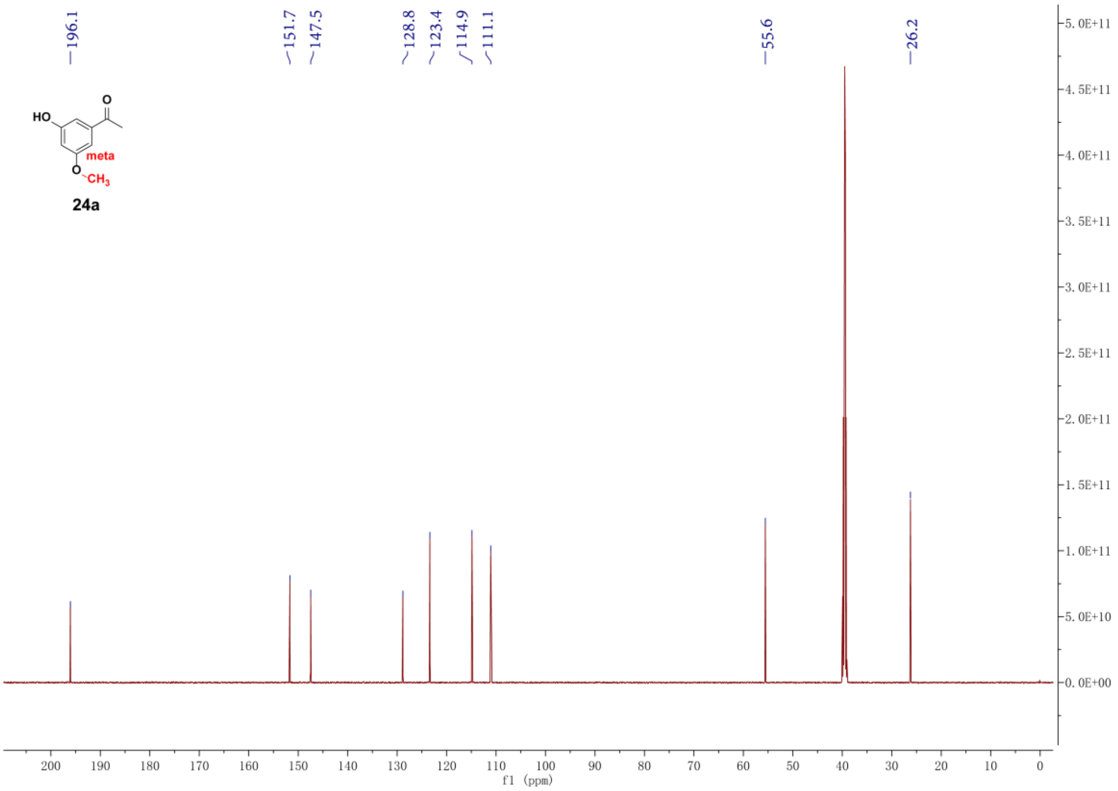

Supplementary Fig 54. ^{13}C NMR spectrum of **24a** in DMSO-d_6 (150 MH)

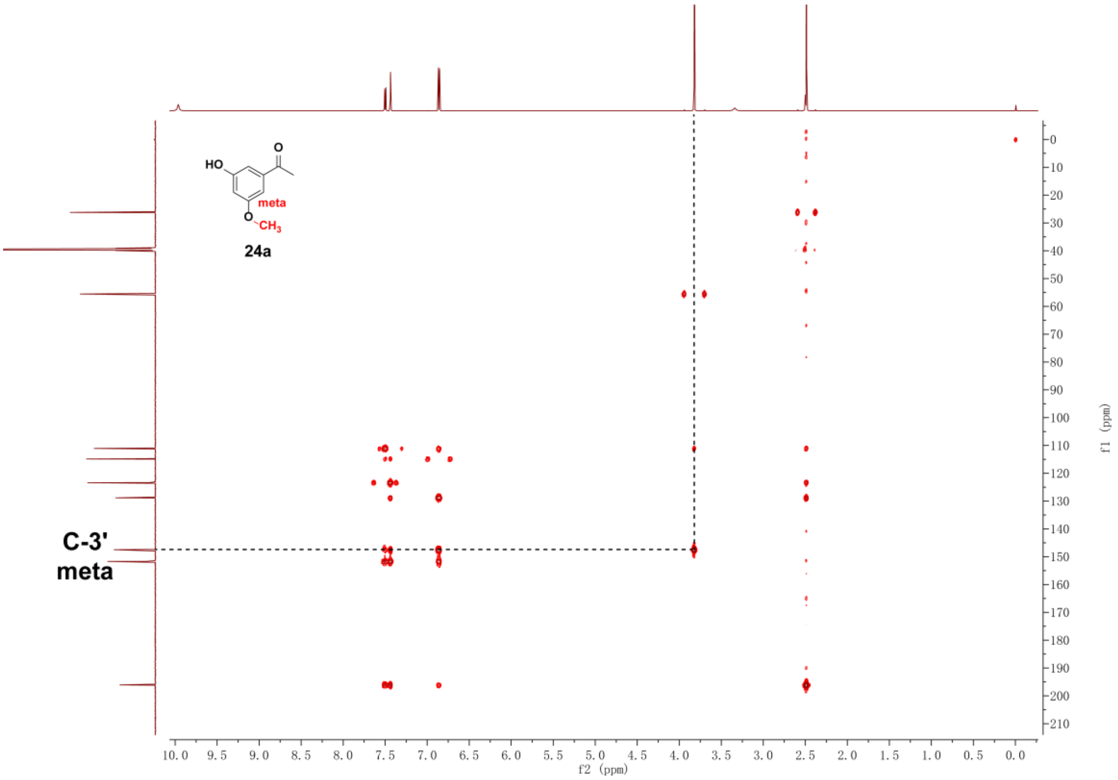

Supplementary Fig 55. HMBC spectrum of **24a** in DMSO-d_6 (150 MHz)

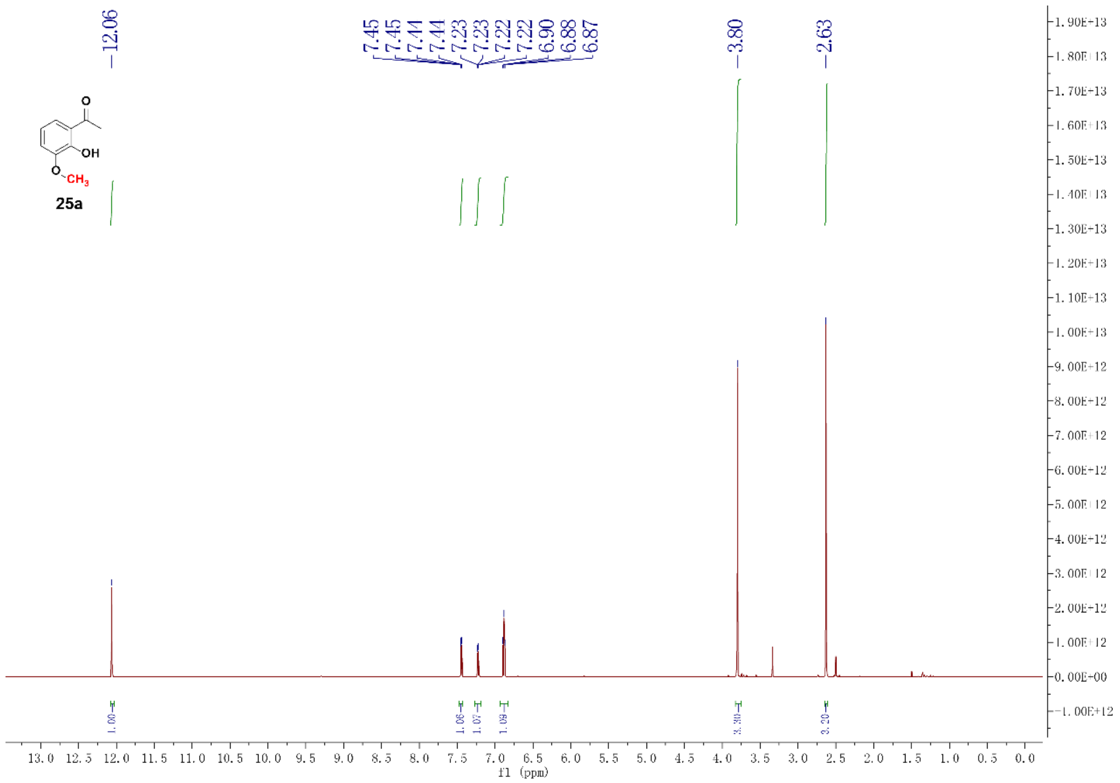

Supplementary Fig 56. ^{1}H NMR spectrum of **25a** in DMSO-d_6 (600 MHz)

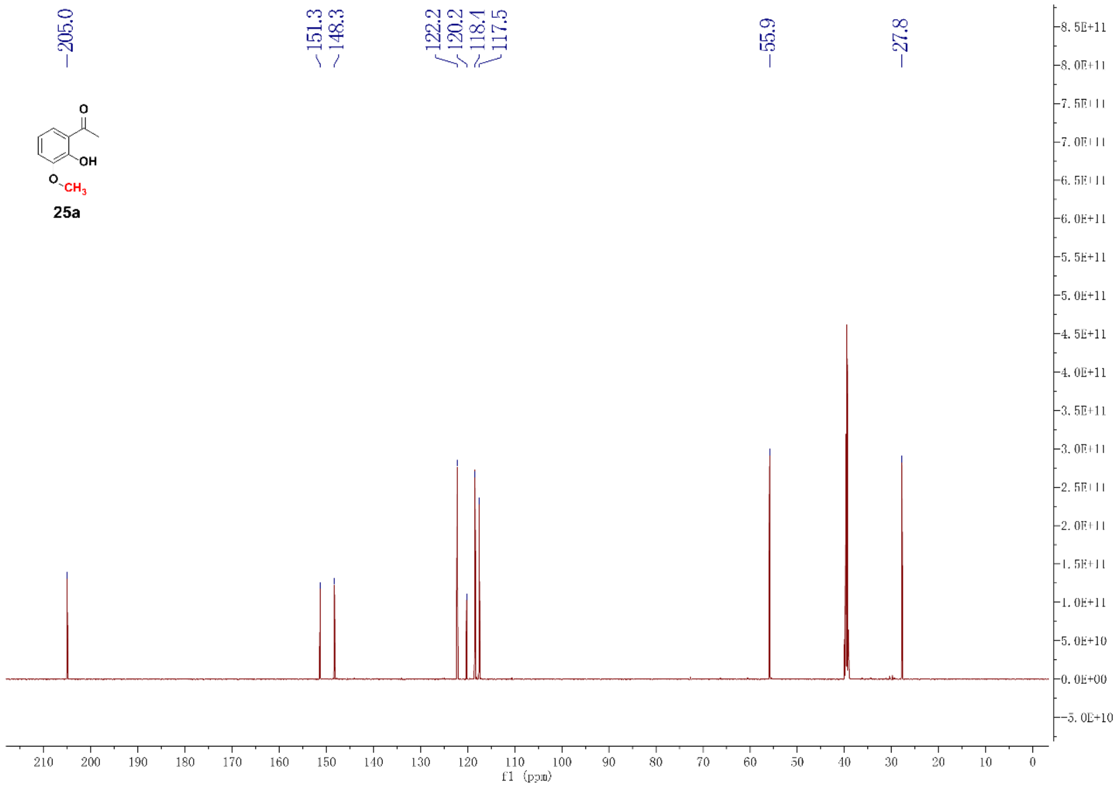

Supplementary Fig 57. ^{13}C NMR spectrum of **25a** in DMSO-d_6 (150 MH)

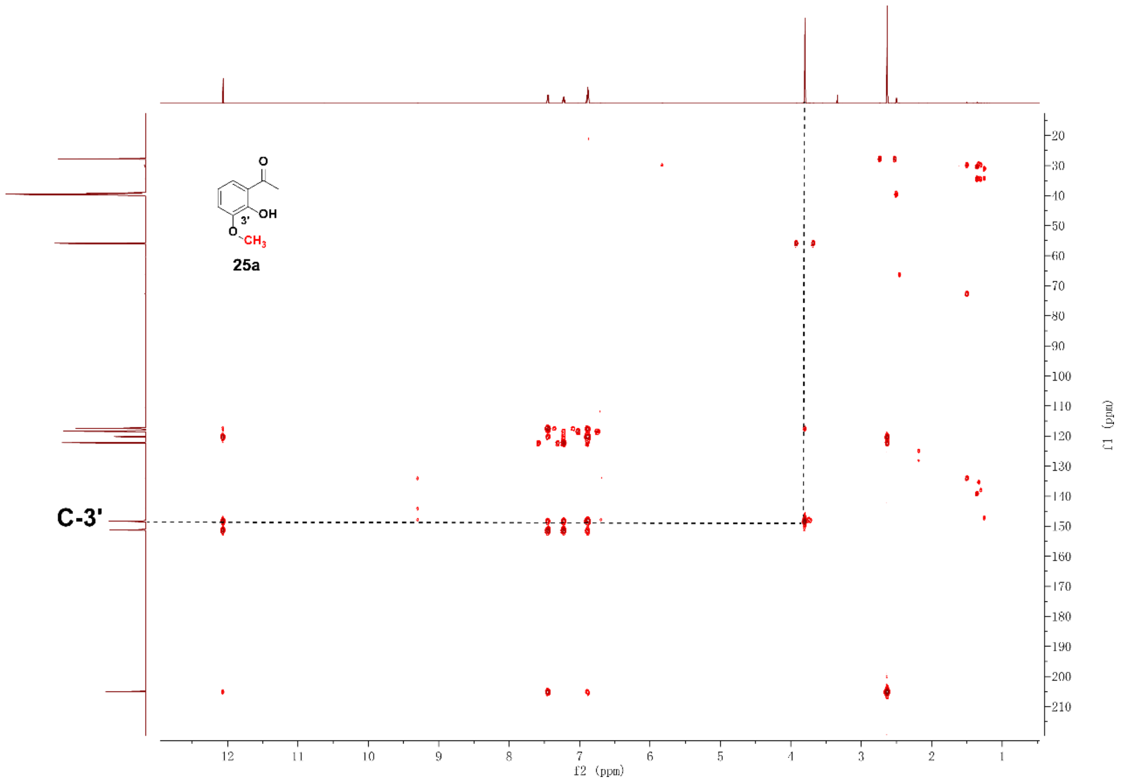

Supplementary Fig 58. HMBC spectrum of **25a** in DMSO-d_6 (150 MHz)

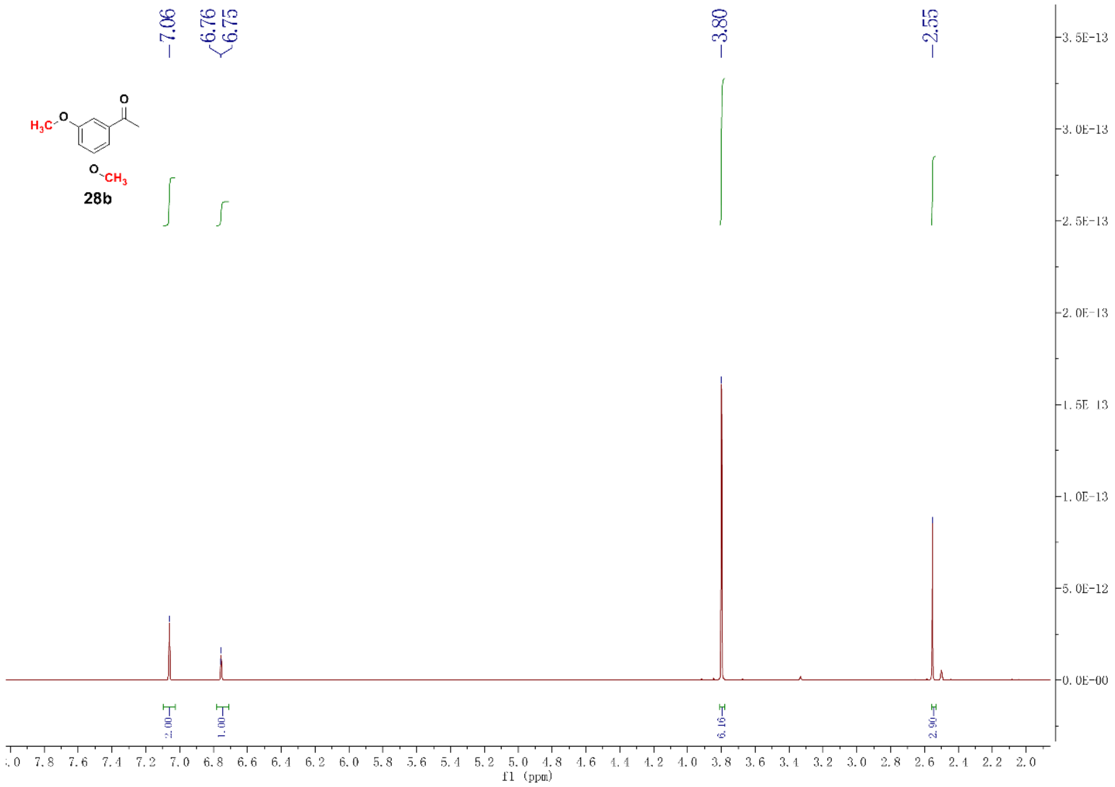

Supplementary Fig 59. ^{1}H NMR spectrum of **28b** in DMSO-d_6 (600 MHz)

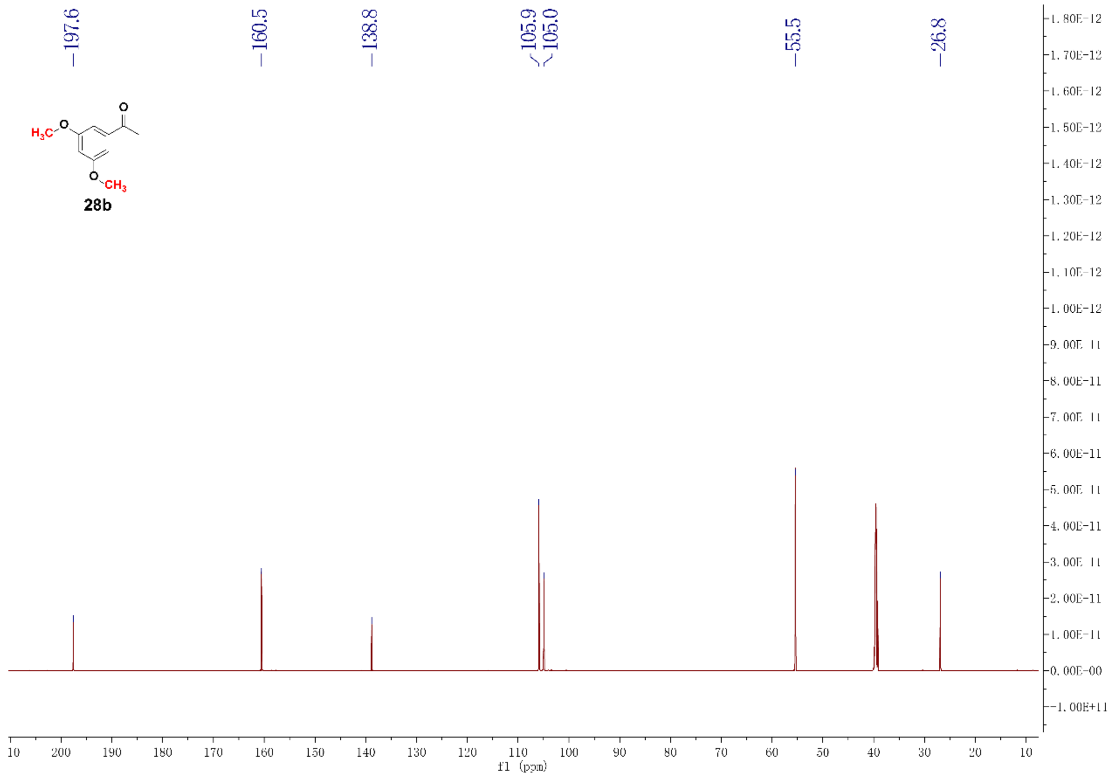

Supplementary Fig 60. ^{13}C NMR spectrum of **28b** in DMSO-d_6 (150 MH)

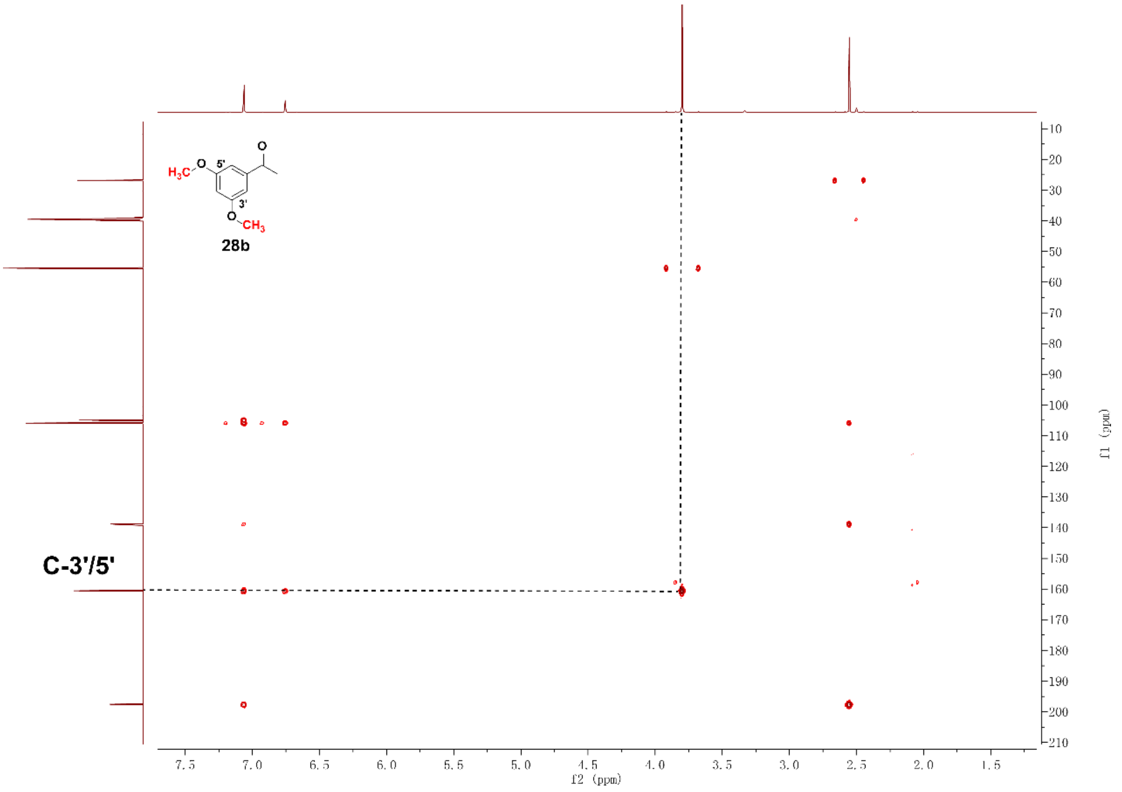

Supplementary Fig 61. HMBC spectrum of **28b** in DMSO-d_6 (150 MHz)

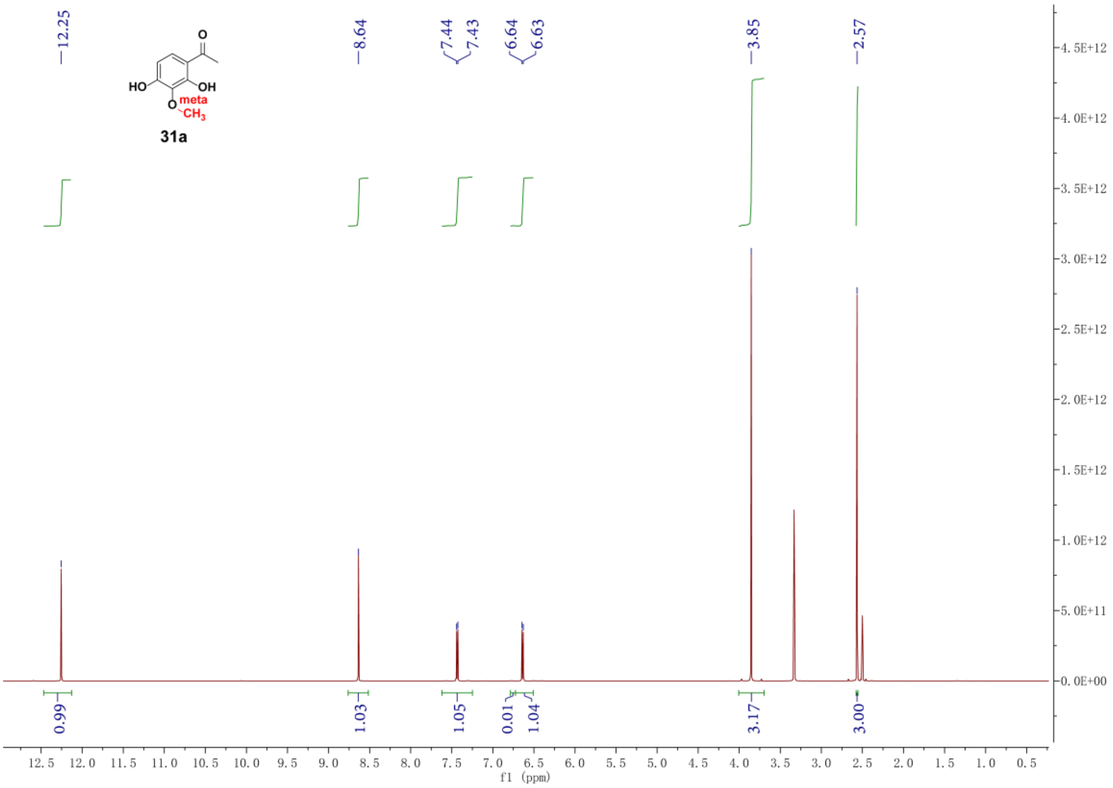

Supplementary Fig 62. ^{1}H NMR spectrum of **31a** in DMSO-d_6 (600 MHz)

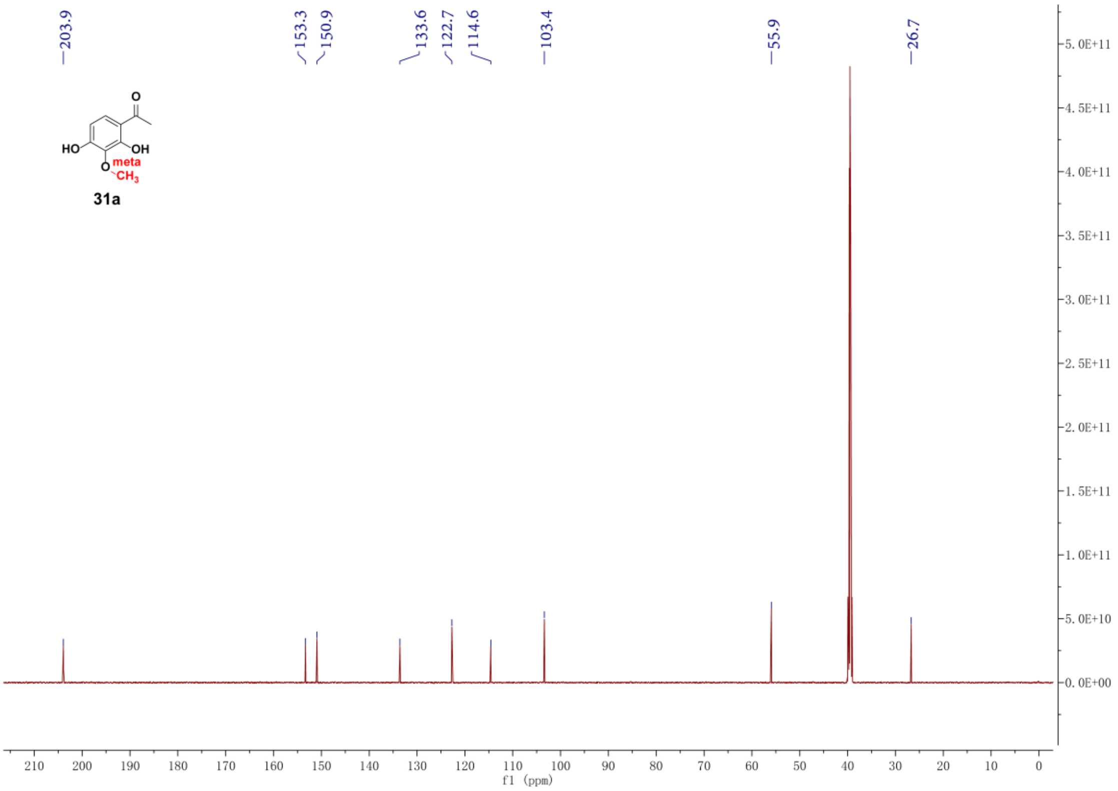

Supplementary Fig 63. ^{13}C NMR spectrum of **31a** in DMSO-d_6 (150 MH)

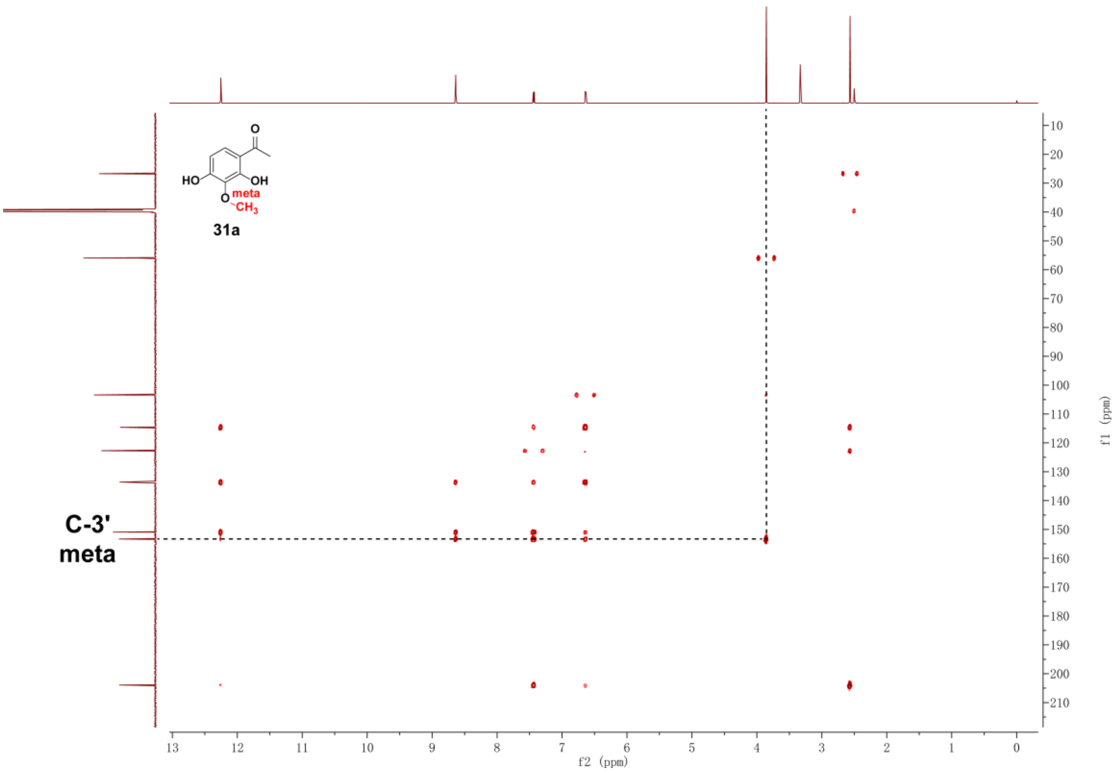

Supplementary Fig 64. HMBC spectrum of **31a** in DMSO-d_6 (150 MHz)

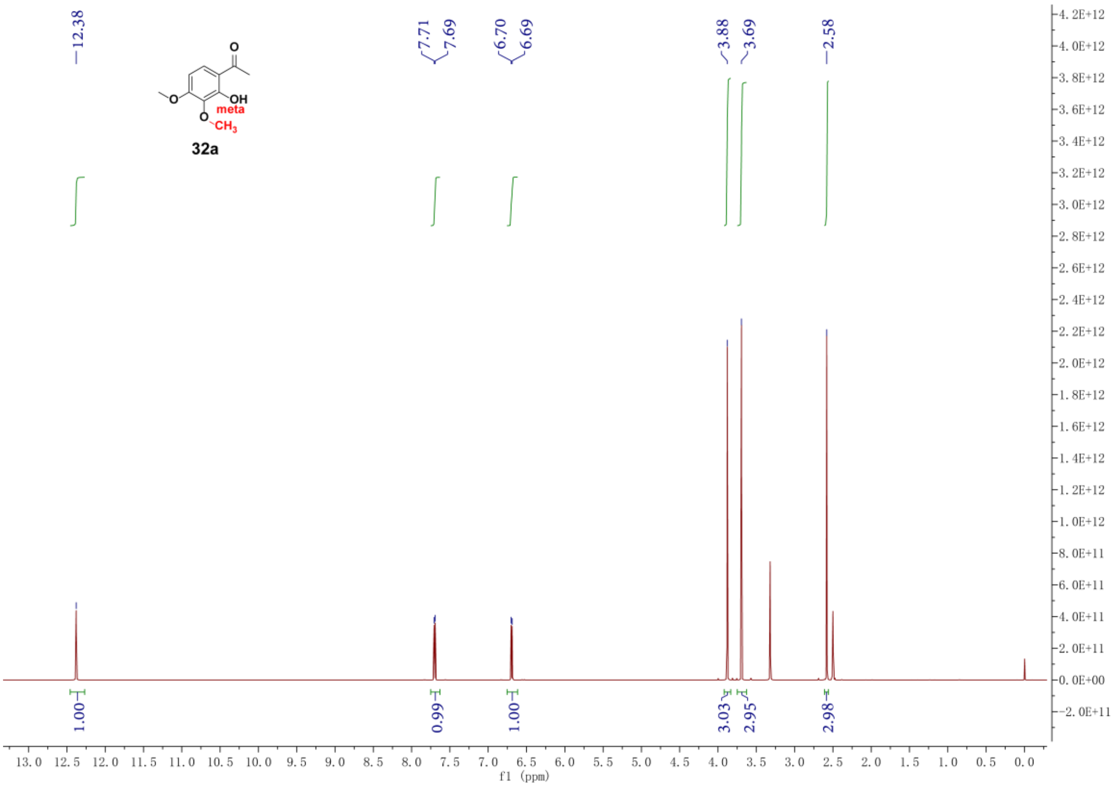

Supplementary Fig 65. ^{1}H NMR spectrum of **32a** in DMSO-d_6 (600 MHz)

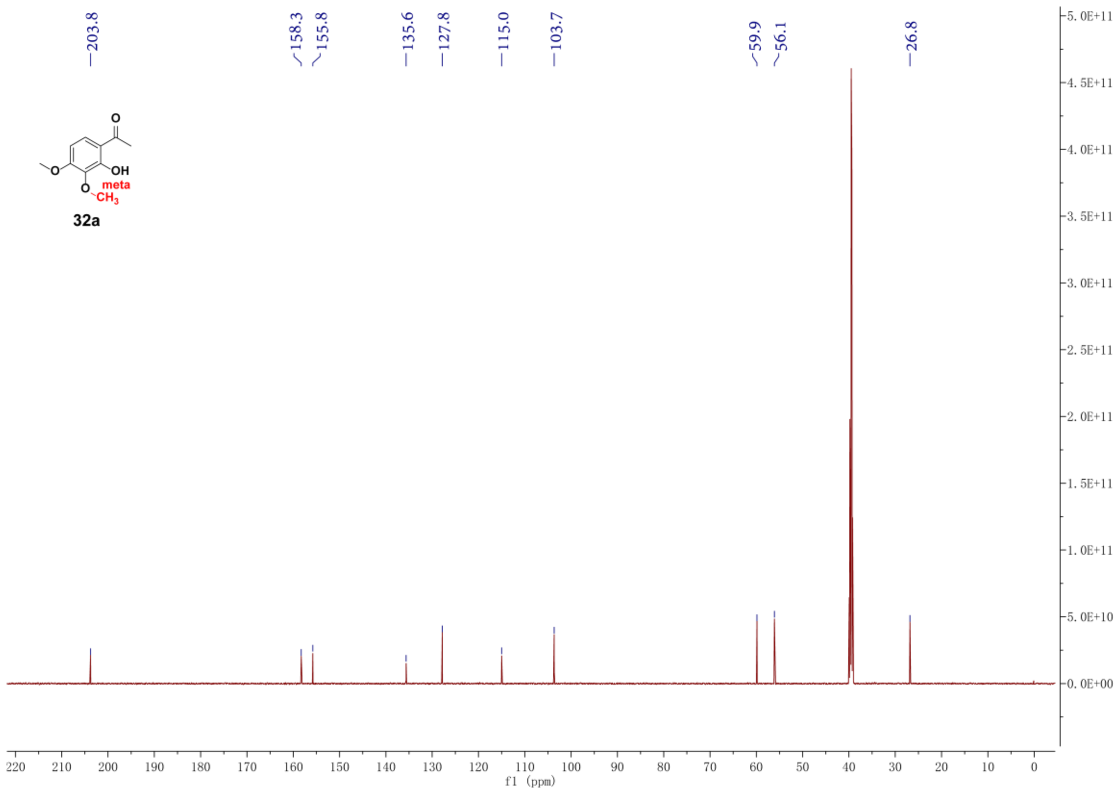

Supplementary Fig 66. ^{13}C NMR spectrum of **32a** in DMSO-d_6 (150 MH)

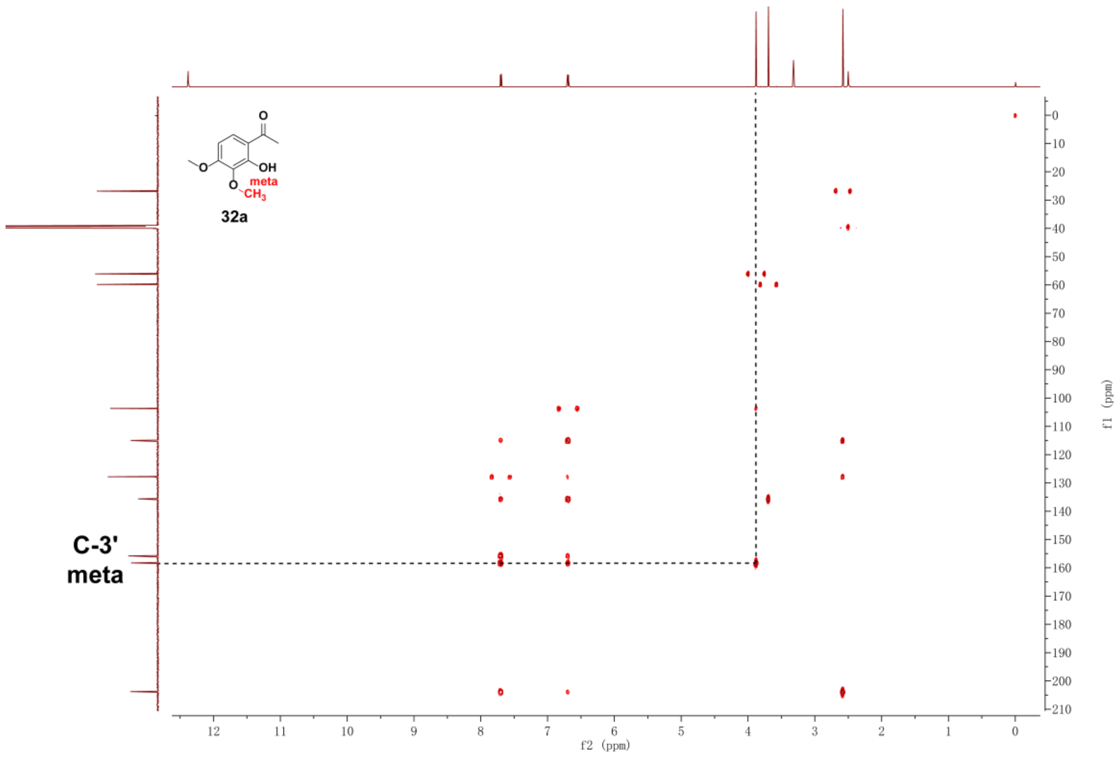

Supplementary Fig 67. HMBC spectrum of **32a** in DMSO-d_6 (150 MHz)

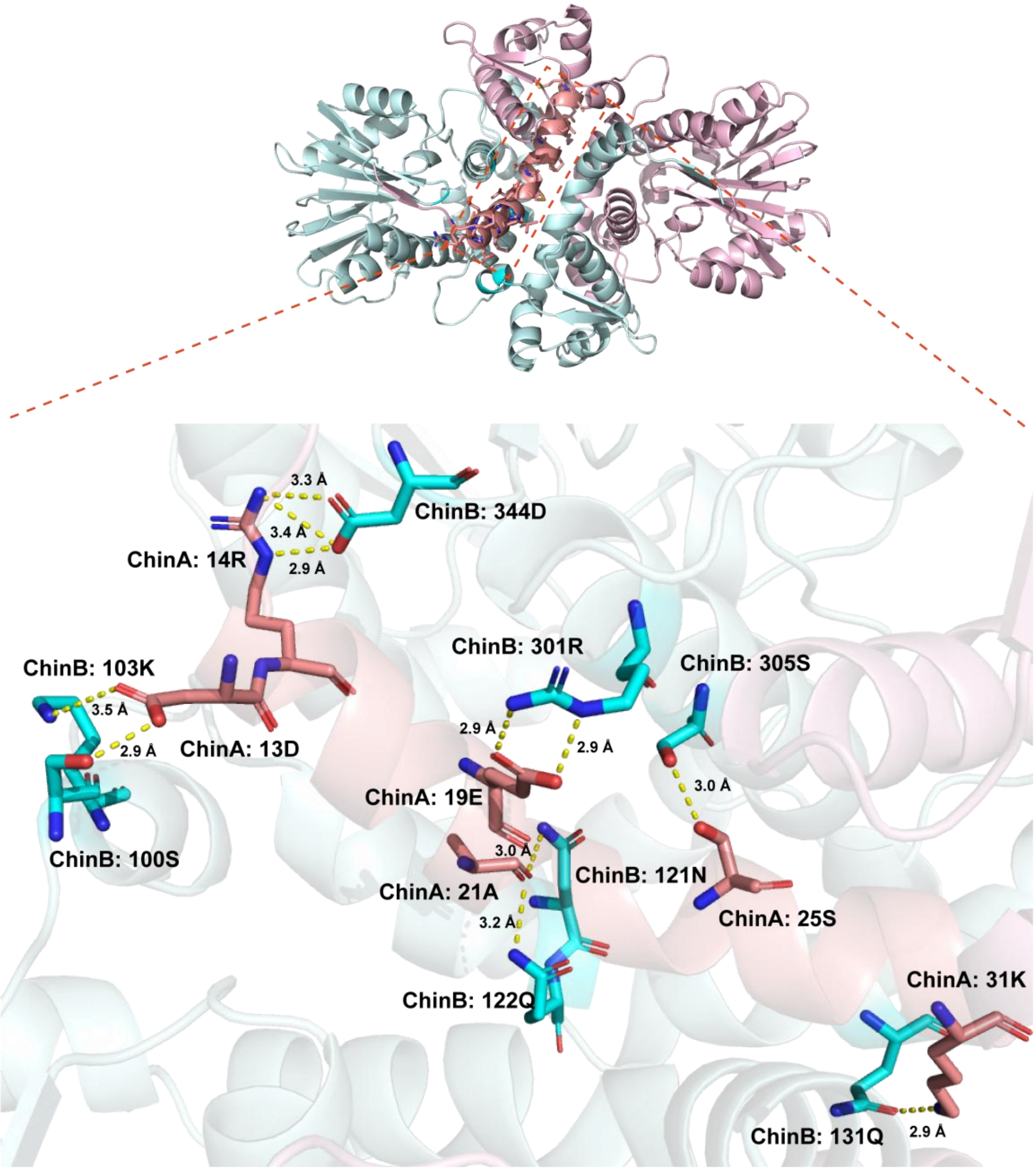

Supplementary Fig 68. Amino acid residues forming hydrogen bonds with helix α1

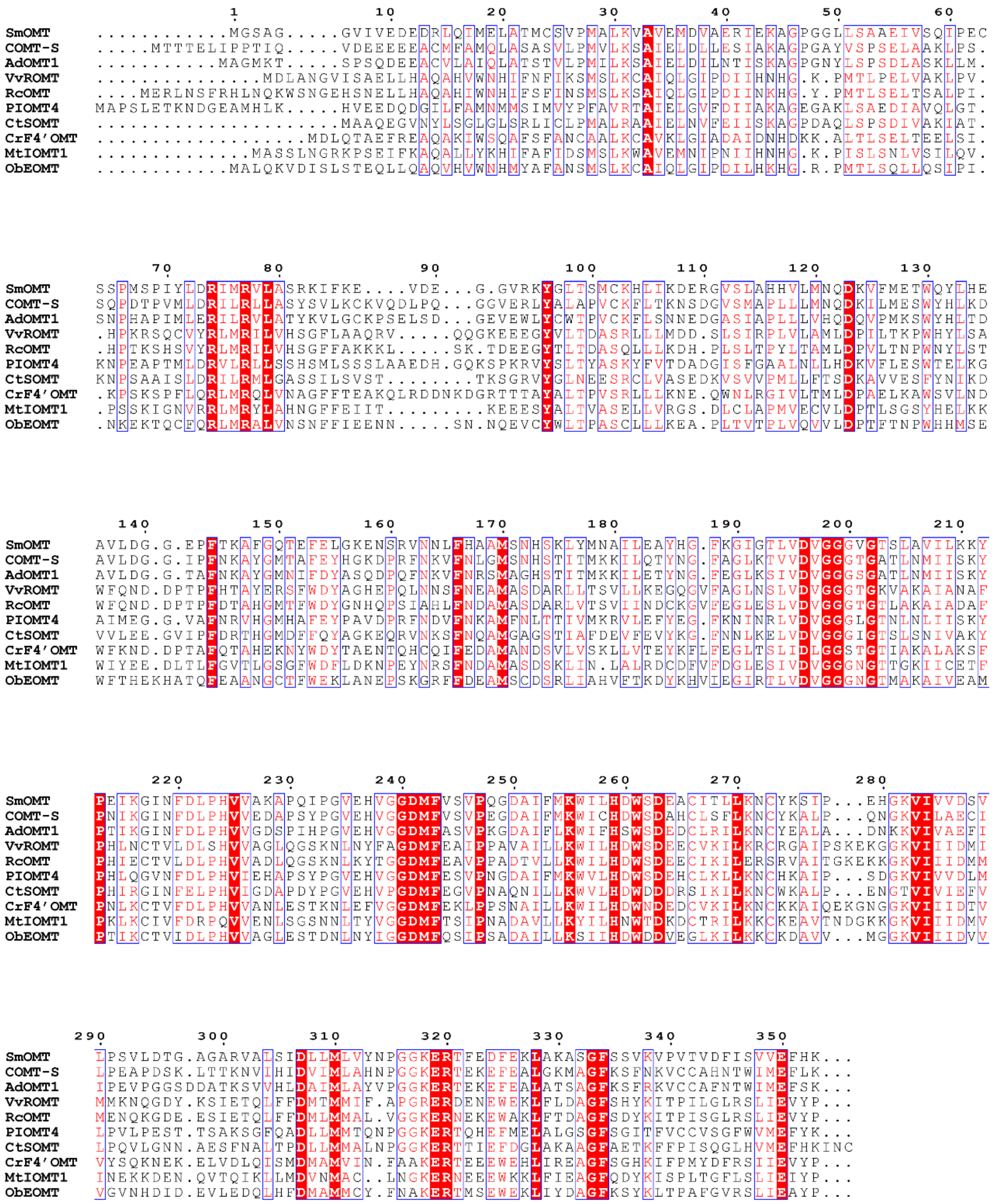

Supplementary Fig 69. Protein sequence alignment of SmOMT with other OMTs. This figure was produced using ENDscript (http://multalin.toulouse.inra.fr/multalin/).

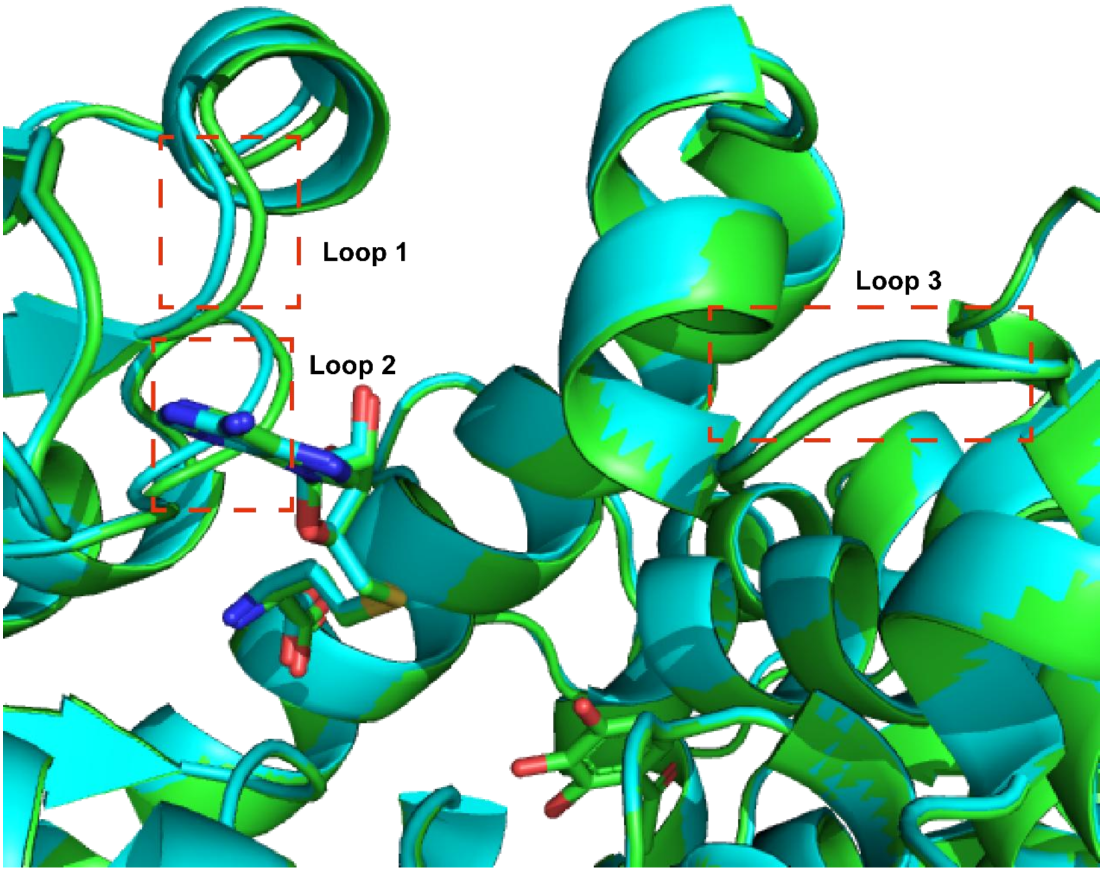

Supplementary Fig 70. The structure superimposition between SmOMT/SAH (cyan) and SmOMT/SAH/**31** (green). Loop-1, Loop-2, and Loop-3 are highlighted using the red dashed rectangles.

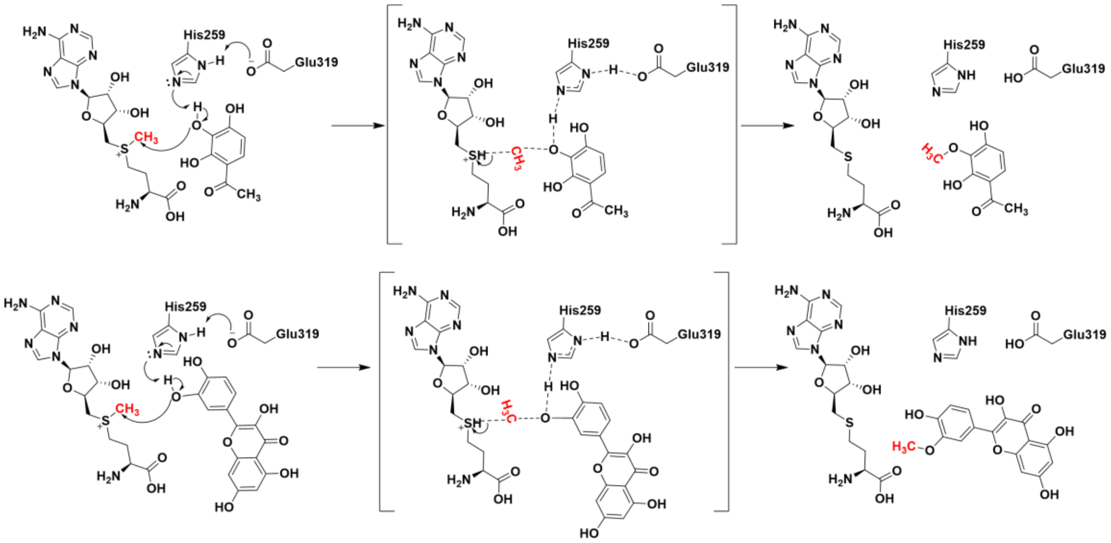

Supplementary Fig 71. Proposed catalytic mechanism of SmOMT.

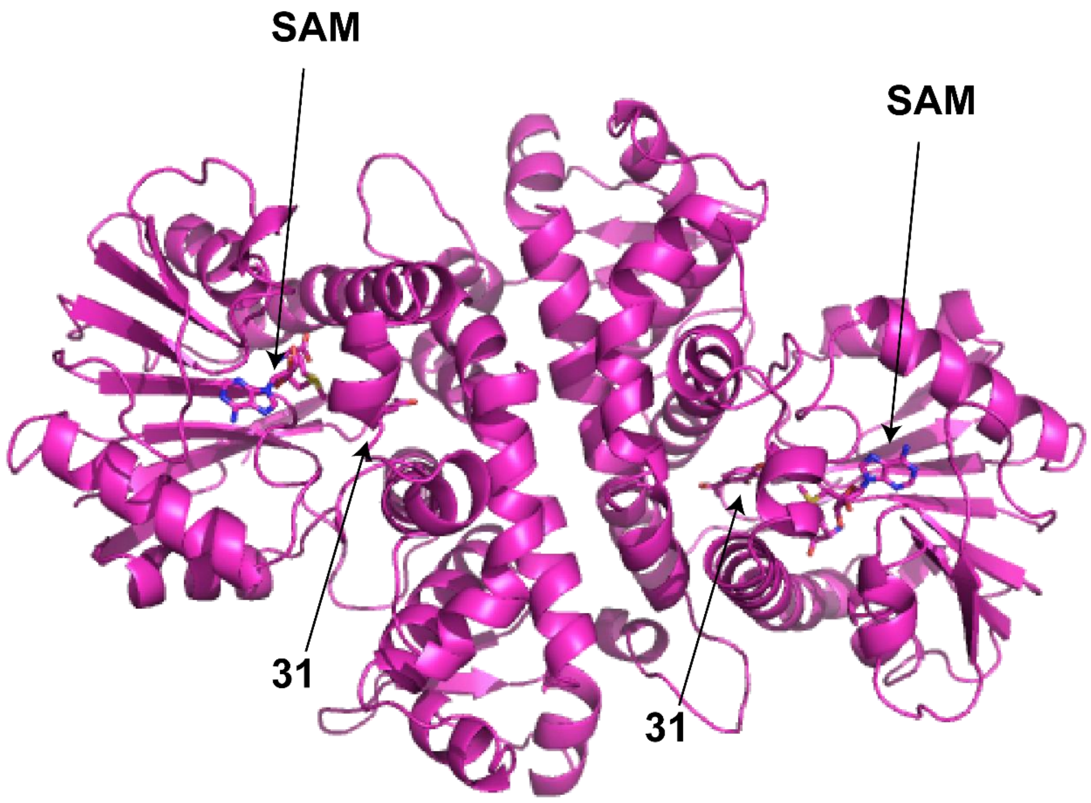

Supplementary Fig 72. The ternary structural model of SmOMT/SAM/**31.**

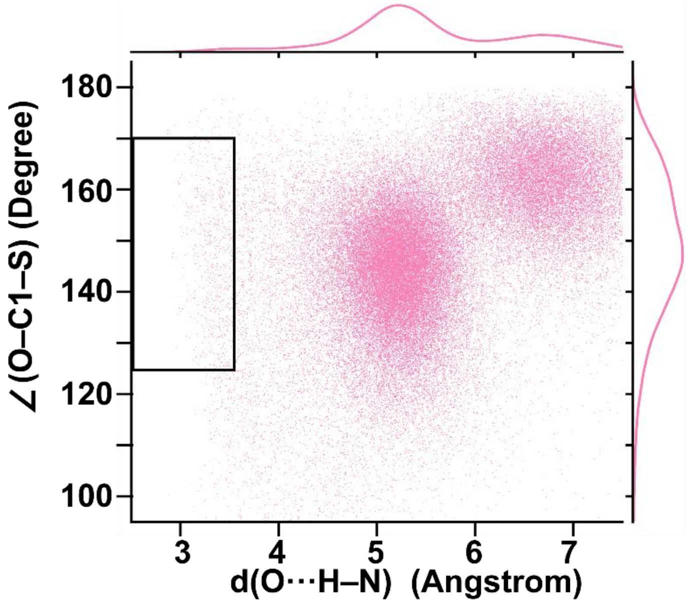

Supplementary Fig 73. KDE mapping of catalytic distance and angle distributions for 2′-*O* methylation of substrate **31**.

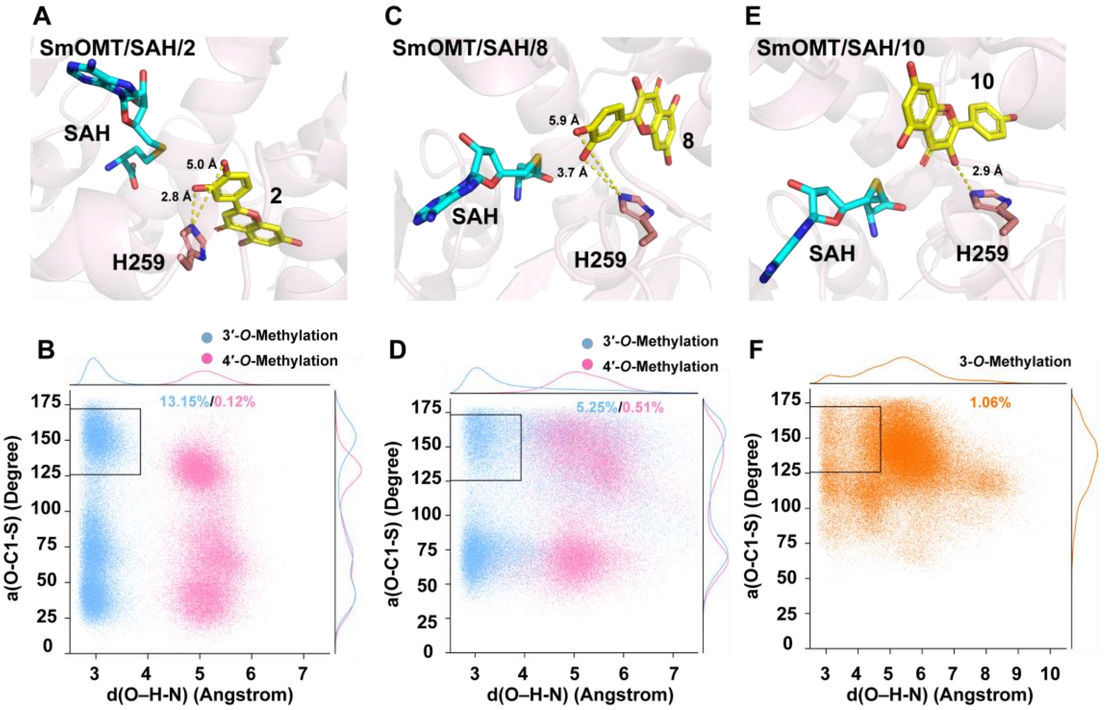

Supplementary Fig 74. Regioselectivity of SmOMT toward flavonoids. **A)** Interactions between SmOMT and **2** in the SmOMT/SAH/**2** docking model. **B)** KDE mapping of catalytic distance and angle distributions for 3′-*O* and 4′-*O* methylation of substrate **2**. **C)** Interactions between SmOMT and **8** in the SmOMT/SAH/**8** docking model. **D)** KDE mapping of catalytic distance and angle distributions for 3′-*O* and 4′-*O* methylation of substrate **8**. **E)** Interactions between SmOMT and **10** in the SmOMT/SAH/**10** docking model. **F)** KDE mapping of catalytic distance and angle distributions for 3-*O* methylation of substrate **10**.

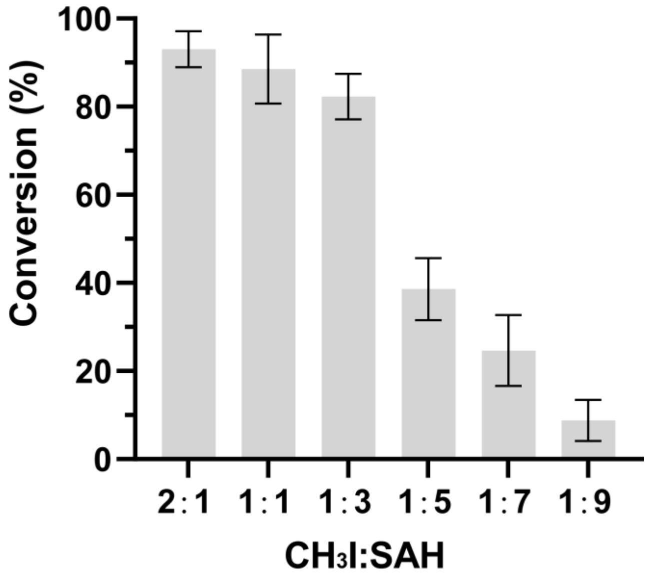

Supplementary Fig 75. Evaluation of SAH as an inhibitor in the coupled system. The conversion of substrate **31** was measured in the presence of CH_3I and SAH at varying molar ratios.

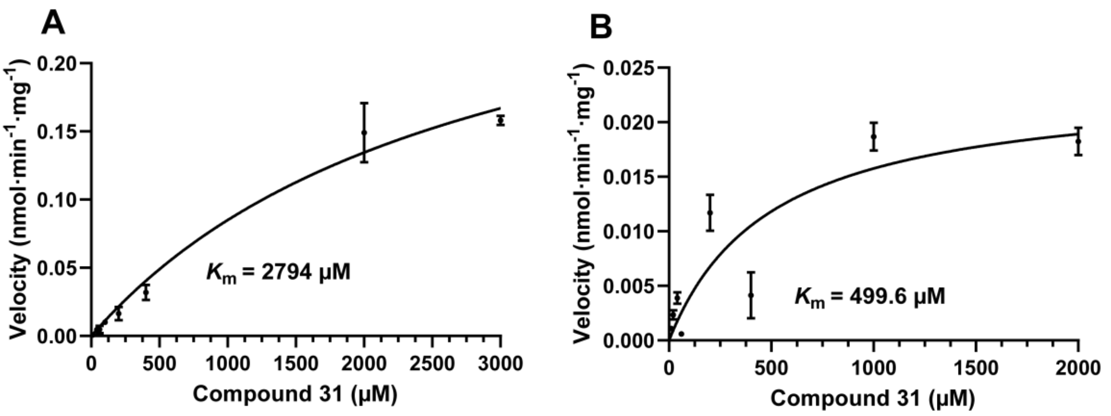

Supplementary Fig 76. Kinetic analysis of substrate 31 under different reaction conditions. A) SmOMT with SAM and 1.5 mM SAH. **B)** the $SmOMT^{M2}$/$AtHMT^{V140T}$ coupled system with 1.5 mM SAH.

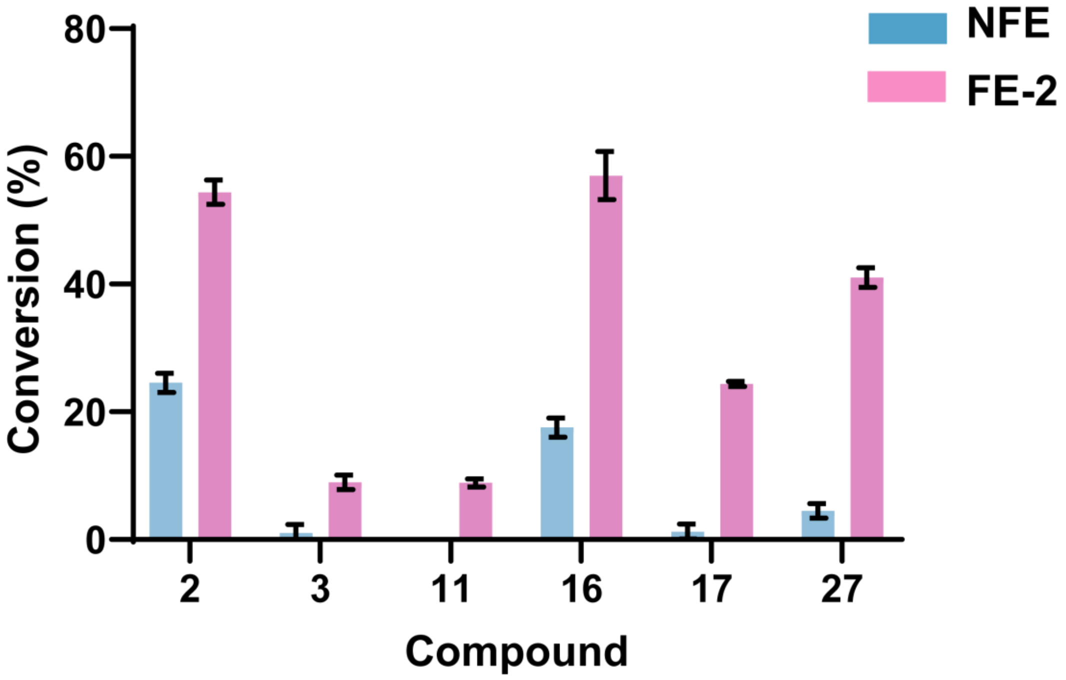

Supplementary Fig 77. Catalytic activity of the NFE and FE2 for six substrates.

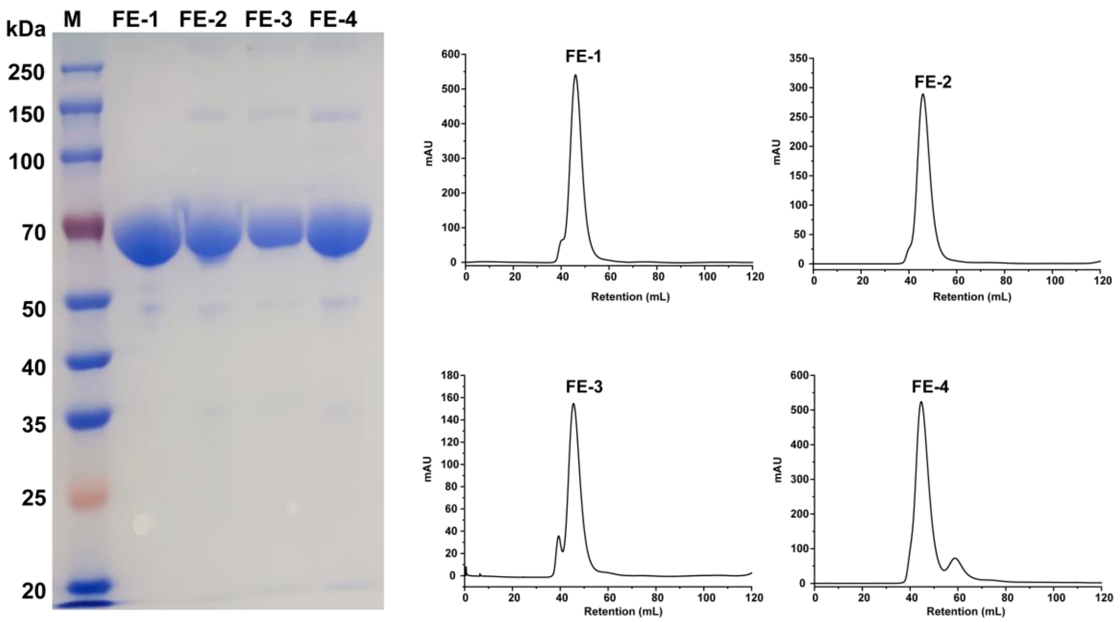

Supplementary Fig 78. SDS-PAGE analysis of the Ni-NTA affinity purification and Size exclusion chromatography profile of four FEs.

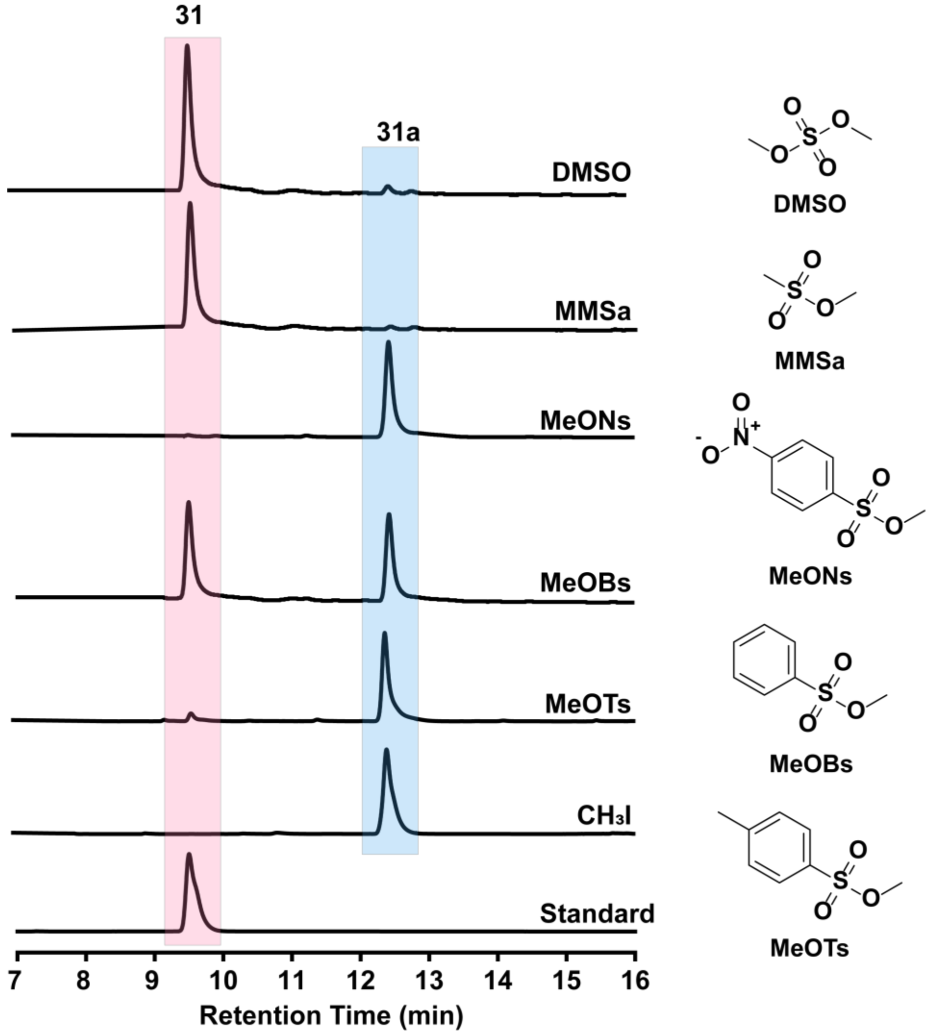

Supplementary Fig 79. HPLC analysis shows the conversion of substrate **31** using the SmOMT^{M2}/AtHMT^{V140T} fusion system with five candidate methyl donors.

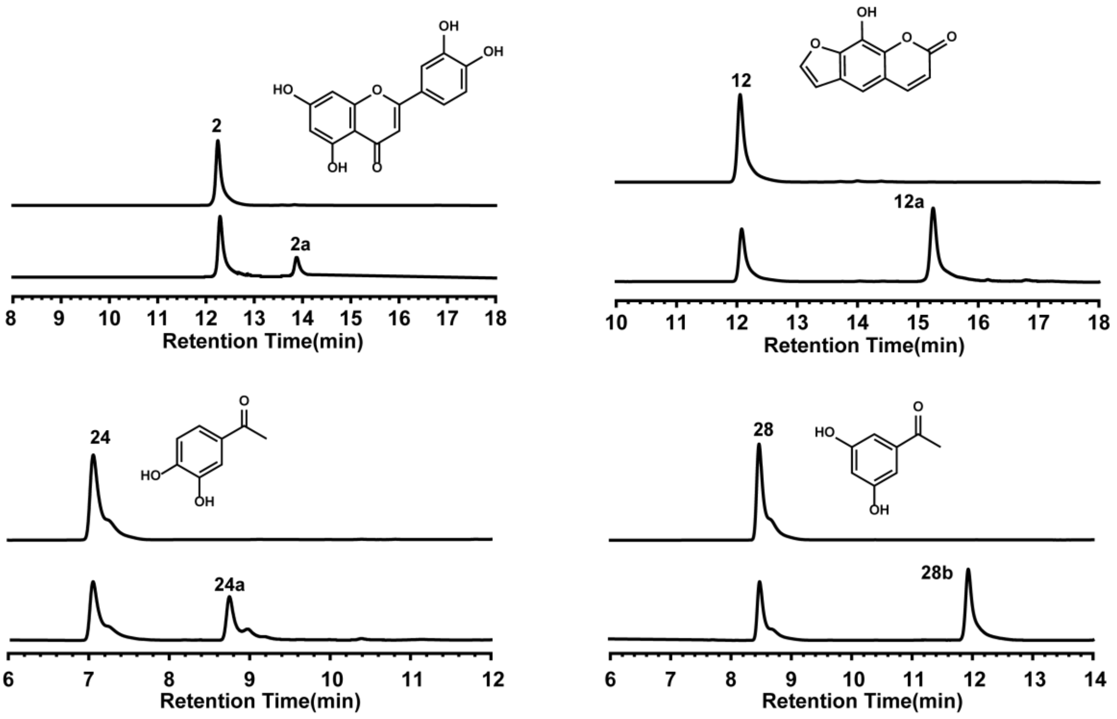

Supplementary Fig 80. Substrate scope of methylation using MeOTs as the methyl donor. HPLC analysis confirms the conversion of substrates **2**, **12**, **24**, and **28**.

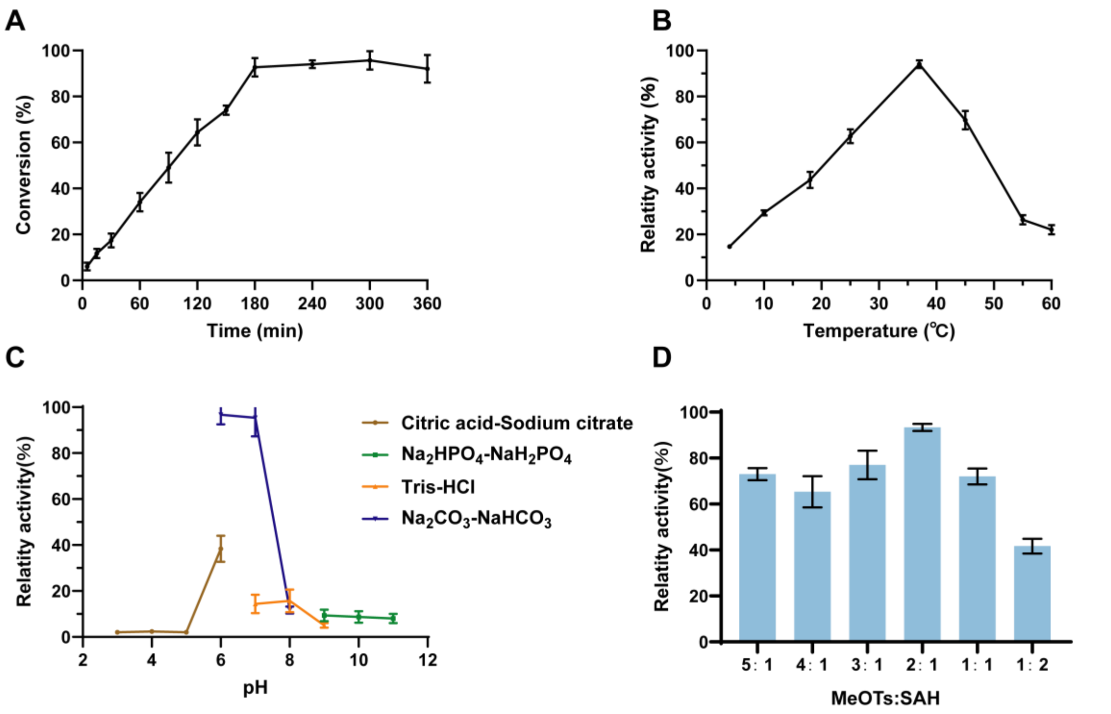

Supplementary Fig 81. Optimization of the SAM-Independent methylation system catalyzed by engineered FE-2. **A)** Time, **B)** Temperature, **C)** pH, **D)** Ratio of MeOTs/SAH.
